# The Comparative Effectiveness and Tolerability of Sphingosine‐1‐Phosphate Receptor Modulators in Patients With Multiple Sclerosis: A Network Meta‐Analysis of Randomized Controlled Trials

**DOI:** 10.1002/acn3.70122

**Published:** 2025-07-04

**Authors:** Faizan Shahzad, Taimoon Rasheed, Momina Riaz Siddiqui, Hamza Hamid, Marwah Bintay Khalid, Haroon Shabbir, Besher Shami, Abdullah, Syed Ijlal Ahmed

**Affiliations:** ^1^ Rawalpindi Medical University Rawalpindi Pakistan; ^2^ University of Aleppo Syrian Arab Republic; ^3^ PGY‐4, SSM Health Saint Louis University School of Medicine St. Louis Missouri USA

**Keywords:** multiple sclerosis, network meta‐analysis, sphingosine‐1‐phosphate receptor modulators

## Abstract

**Background:**

Sphingosine‐1‐phosphate receptor modulators (S1PRM) are used to treat relapsing multiple sclerosis (MS). Each drug has a different S1PR‐subtype selectivity. They target the G‐protein coupled S1P receptors and exert significant immunomodulatory effects, such as preventing the formation of new CNS lesions and the reactivation of pre‐existing lesions.

**Objective:**

This study aims to explore the efficacy and safety of S1PRM in treating MS.

**Methods:**

A systematic literature search of PubMed, Embase, and Cochrane databases was conducted in August 2024. Randomized Controlled Trials that evaluated the efficacy of S1PRM in patients with MS were included. Changes in Annualized Relapse Rate and incidence of adverse effects were chosen as primary outcomes. Standardized mean differences (SMD) and odds ratio (OR) were calculated. Confidence interval was kept at 95%. Individual interventions were compared using the Surface Under Cumulative Ranking Curve (SUCRA). The risk of bias was assessed by the Cochrane risk‐of‐bias tool for randomized trials (RoB 2).

**Results:**

The search query resulted in a total of 1750 studies. After screening, 17 studies were included in the final analysis, with a population of 16,006. Fingolimod (1.25 mg) was significantly associated with a decreased ARR (SMD = −0.4422, 95% CI = [−0.5450 to −0.3394], *p*‐value < 0.0001, SUCRA = 92.65%). Whereas, ozanimod (1 mg) was associated with the lowest number of new Gadolinium‐enhanced lesions (SMD = −0.6516, 95% CI = [−0.8944 to −0.4087], *p*‐value < 0.0001, SUCRA = 86.38%). Siponimod (1.25 mg) was associated with the least number of adverse events (OR = 0.4606, 95% CI = [0.1893 to 1.1205], *p* = 0.0874, SUCRA = 93.20%). Almost all of the studies had a low risk of bias.

**Conclusion:**

Fingolimod (1.25 mg) and ozanimod (1 mg) had the best efficacy, and siponimod (1.25 mg and 0.25 mg) had the best safety profile among the S1PRM. Further longitudinal studies should be conducted to assess the long‐term effects of these drugs on patient‐reported outcomes.

## 1 Introduction

Multiple Sclerosis (MS) is a neurodegenerative disease caused by autoimmune responses leading to chronic inflammation and demyelination. It is classically characterized by recurrent relapses of clinical symptoms followed by periods of recovery leading to progressive degeneration over time [1]. The goal of MS therapy is to control the autoimmune response, inflammation, neurodegeneration and symptoms from recurrent relapses. Therefore, long‐term disease modifying therapies (DMTs) are often suggested in such cases.

Sphingosine‐1‐receptor modulators are one of the most promising DMTs [2]. Sphingosine‐1‐phosphate (S1P) is a molecule derived from sphingosine through enzymes called sphingosine kinase 1 and 2 (Sk1, Sk2). It is a key cell‐signaling molecule, which plays a crucial role in the immune cell movement. It works by interacting with S1P receptors (S1PRs), specifically S1PR‐1, to regulate the exit of lymphocytes from the peripheral lymphoid organs [3]. This makes it an excellent treatment option for inflammatory diseases such as ulcerative colitis, Crohn's disease, rheumatoid arthritis and even systemic lupus erythematosus. Since MS is an immune‐mediated inflammatory response, S1PR modulation is a promising treatment option for it [4].

Fingolimod is a first generation S1PR modulator, which is also the first oral treatment for MS. It has been shown to have significant immunomodulatory effects, such as preventing new CNS lesions formation and the reactivation of pre‐existing lesions, as well as reducing the annualized relapse rate. Its use is restricted, however, due to concerns with adverse effects such as decreased heart rate and atrioventricular block, increased risk of serious infections, macular edema or development of skin neoplasms. Newer generation S1PR modulators, such as siponimod, ozanimod, ponesimod, laquinimod, and amiselimod, aim to address these issues by providing greater receptor selectivity [5].

We conducted a network meta‐analysis (NMA) to systematically evaluate and compare the efficacy and safety profiles of S1PR modulators in the treatment of MS. The NMA utilizes a connected evidence network, which enables comparisons of drugs even if they have not been directly compared in head‐to‐head studies. The aim of this analysis is to provide a comprehensive, ranked assessment of these therapies and identify the most optimal S1PR modulator by assessing their relative performance. The individual efficacy and safety profiles of these drugs have been well documented. However, there is limited data on the comparative effectiveness and tolerability of the individual drugs. With so many available options, there is hot debate on which medication provides the correct balance of efficacy and adverse events, and this analysis would help guide clinical decision‐making in identifying the most appropriate intervention.

## 2 Methods

### 2.1 Registration

**FIGURE 1:**
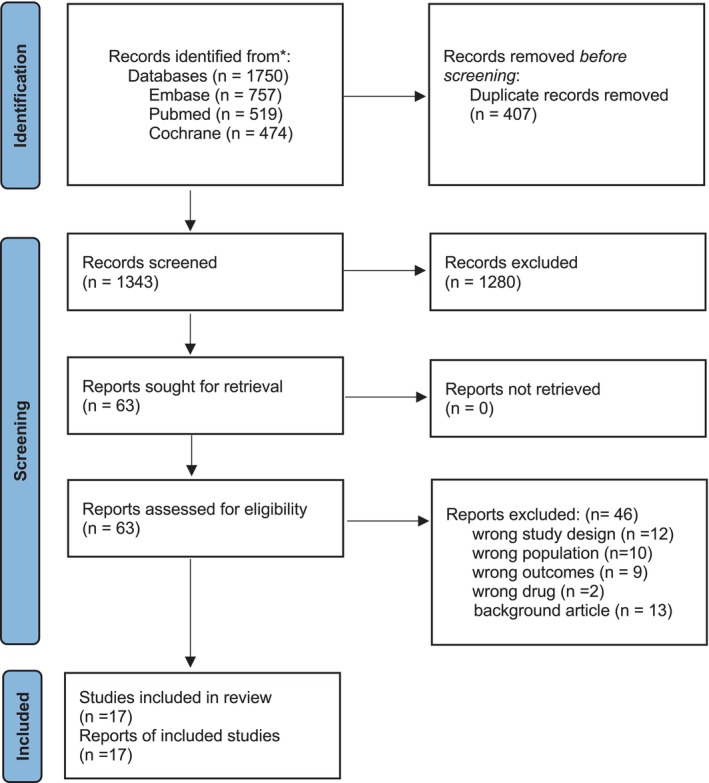
PRISMA Flow Chart.

This systematic review and network meta‐analysis is in accordance with the Preferred Reporting Items for Systematic Reviews and Meta‐Analyses (PRISMA) statement [6] Figure 1. The study protocol was registered (registration number) with the International Prospective Register of Systematic Reviews (PROSPERO).

### 2.2 Search Strategy

Three electronic databases (PubMed, EMBASE, Cochrane Central Register of Controlled Trials [CENTRAL]) were searched. The search strategy was constructed around the PICOS tool: (P) Population: adults with multiple sclerosis; (I) Intervention: sphingosine 1 receptor modulator; (C) Comparator: placebo or interferon beta; (O) Outcomes: as mentioned in the eligibility criteria section and study type: RCTs. The PICOS table and search strategy are given as Table S1. In addition to the databases, the reference lists of included articles were scanned for articles that met the inclusion criteria.

### 2.3 Eligibility Criteria

RCTs published in scientific peer‐reviewed papers prior to November 2024 were included. The study included only parallel RCTs with the population of adults with MS, intervention as S1PR modulators, and comparators as placebo or interferon beta, while single‐arm trials, case reports, and conference papers were excluded. The outcomes of efficacy included Annualized Relapse Rate (ARR), number of new or emerging T2 lesions, number of gadolinium‐enhancing lesions, and change in brain volume, with ARR being the primary outcome of efficacy. The primary outcome of safety was the incidence of adverse events (AEs); secondary outcomes included serious adverse events (SAEs) and discontinuations due to adverse events (DCAEs). The study population included MS patients treated with sphingosine 1 receptor modulators. In accordance with prior research (Li et al., 2019), we selected studies with follow‐up periods between 6 and 24 months [7].

### 2.4 Study Selection and Data Extraction

Endnote X8 literature management software was used to manage the literature search records. The selection process was split into two phases. In the initial phase (primary or abstract//title screening), the yielded articles were independently screened by two reviewers based on the titles. A third reviewer was consulted in case of any discrepancies. In Phase 2 (full‐text or secondary screening), two independent reviewers reviewed all the articles selected from the initial phase based on predetermined inclusion criteria. A third reviewer or consensus‐based discussion was used to resolve any disagreement. The reviewers used Google Sheets to extract the key study parameters. The data extracted during the screening process include: the first author's full name, publication year, the author's country of origin, assessment of the risk of bias, and outcome indicators.

### 2.5 Risk of Bias in Individual Studies

Two reviewers assessed the risk of bias (ROB) independently following the revised Cochrane risk‐of‐bias tool for randomized trials (RoB 2) tool. The following five domains were considered as follows: (i) bias due to the randomization process, (ii) deviation from intended intervention, (iii) missing outcome data, (iv) measurement of outcome, (v) selection of the reported result and overall risk of bias. The trials were categorized into high risk, low risk, or moderate risk using the ROB 2 tool. The overall quality of evidence was assessed by the GRADE Approach for Network Meta‐Analysis [8].

### 2.6 Outcome Definition and Endpoint Standardization

To ensure comparability across included studies, we established standardized definitions for all primary and secondary outcomes prior to data extraction. The annualized relapse rate (ARR) was defined as the total number of confirmed relapses per patient‐year. New gadolinium‐enhancing (Gd+) lesions were defined as newly detected lesions on T1‐weighted MRI with contrast compared to baseline. Reduction in brain volume was expressed as the percentage change from baseline over the study period. Relapse‐free patients referred to the proportion of patients who remained free of clinical relapses during the study duration. Adverse events (AEs) and serious adverse events (SAEs) were recorded as the total number of such events occurring during the study. Withdrawal due to adverse events was defined as the proportion of patients who discontinued treatment because of treatment‐related adverse effects.

### 2.7 Statistical Analysis

The Meta and Netmeta packages in R Studio with Rv.4.4.1 were used for data analysis. A random‐effects model was used to pool the effect estimates for the specified outcomes. Odds ratios (OR) and standardized mean differences (SMD) with 95% confidence intervals (CIs) were presented. Network plots were created to represent the presence of direct and indirect evidence. Treatment ranking was based on the p‐score, while the surface under the cumulative ranking curve (SUCRA) was used for treatment ranking. I [2] statistic and the Q test were used to assess the heterogeneity and consistency of the studies Table 1, respectively.

**TABLE 1:** Consistency and heterogeneity assessment.

Outcome	Total Q statistic (*p*)	Within design Q statistic (*p*)	Between designs Q statistic (*p*)	I^2^
Annualized Relapse Rate (ARR)	1.43 (0.9976)	0.38 (0.9434)	1.04 (0.9839)	0%
Mean new Gd‐enhanced lesions from baseline	27.81 (0.0035)	23.27 (0.007)	4.54 (0.4741)	60.4%
Mean reduction in brain volume	11.87 (0.1049)	6.05 (0.1954)	5.82 (0.1207)	41%
Proportion of relapse‐free patients	32.93 (0.003)	3.17 (0.5293)	29.75 (< 0.0001)	69.6%
Adverse events	16.47 (0.2852)	5.93 (0.4315)	10.55 (0.2286)	15%
Serious adverse events	14.69 (0.2589)	9.08 (0.1691)	5.61 (0.4685)	18.3%
Withdrawal due to adverse events	26.78 (0.0206)	9.70 (0.1377)	17.07 (0.0294)	47.7%

## 3 Results

### 3.1 Search Results

A systematic search of the databases PubMed, Cochrane, and Embase using the MeSH keywords and entry terms of “Multiple Sclerosis” and Sphingosine 1 receptor modulators (“Fingolimod”, “Laquinimod”, “Ozanimod”, and “Ponesimod”) was conducted in October 2024. A total of 1750 articles were retrieved. After removing 407 duplicates, 1343 articles were included for primary screening. After the initial primary screening, 1280 articles were removed, and 63 were included for full‐text screening. During the full‐text screening, 46 articles were removed, and 17 studies were included [9, 10, 11, 12, 13, 14, 15, 16, 17, 18, 19, 20, 21, 22, 23, 24, 25]. The PICOS table and search strategy are in Table S1.

### 3.2 Study Characteristics and Consistency

**FIGURE 2:**
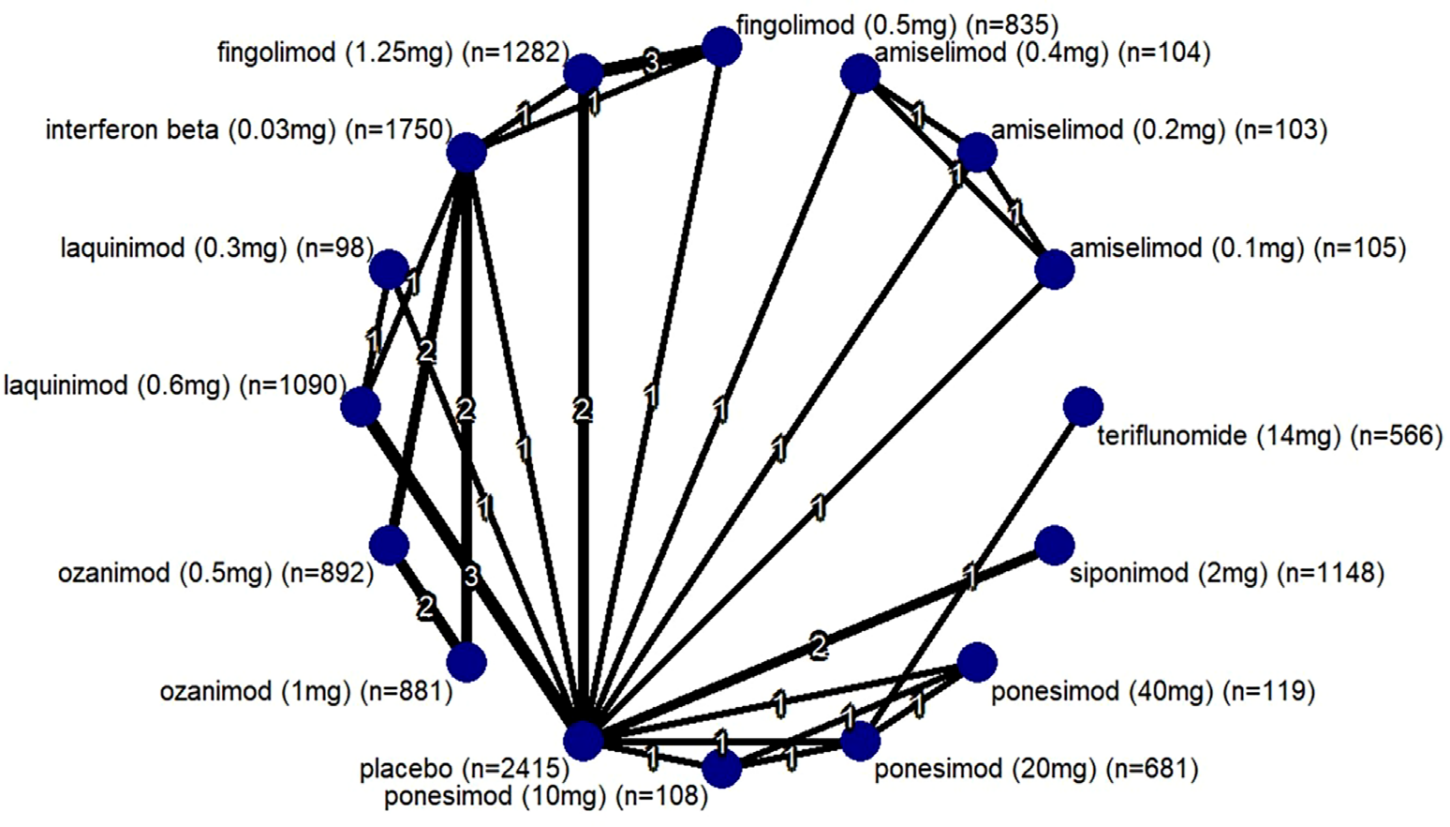
Network plot.

A total of 17 studies with 16,006 participants were part of this network meta‐analysis. The time of follow‐up ranged from 6 months to 24 months. To address any heterogeneity that may arise due to wide range of follow‐up time, we have performed meta‐regression. Significant heterogeneity was observed in “New Gd‐enhanced lesions from baseline” and “Proportion of relapse free patients”. Moderate heterogeneity was observed in “Mean reduction in brain volume and withdrawal due to adverse events”. Low heterogeneity was observed in incidence of adverse events and serious adverse events. No heterogeneity was observed in ARR. Figure 2 shows the network plot. Node‐split analysis was performed to assess the inconsistency between direct and indirect evidence. Minimal evidence of inconsistency was reported, thus contributing to the robustness of the analysis. The results of node‐split analysis are given as a Supporting Information with the name “NMA SPLIT DATA”.

### 3.3 Outcomes

#### 3.3.1 Annualized Relapse Rate (ARR)

**FIGURE 3:**
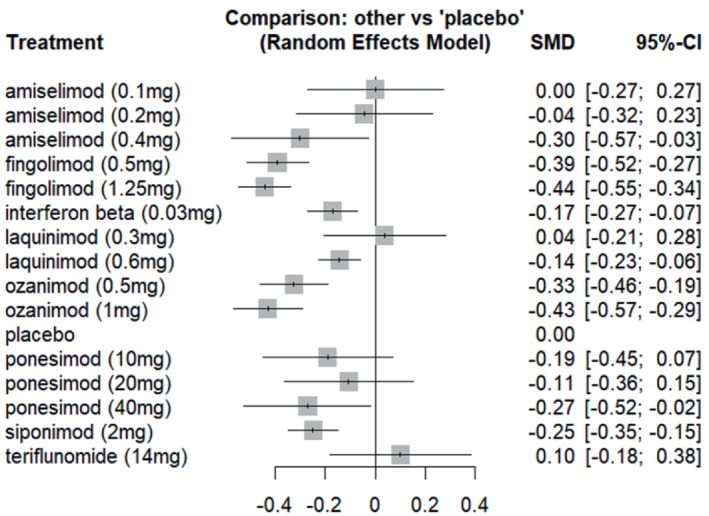
Forest plot.

**FIGURE 4:**
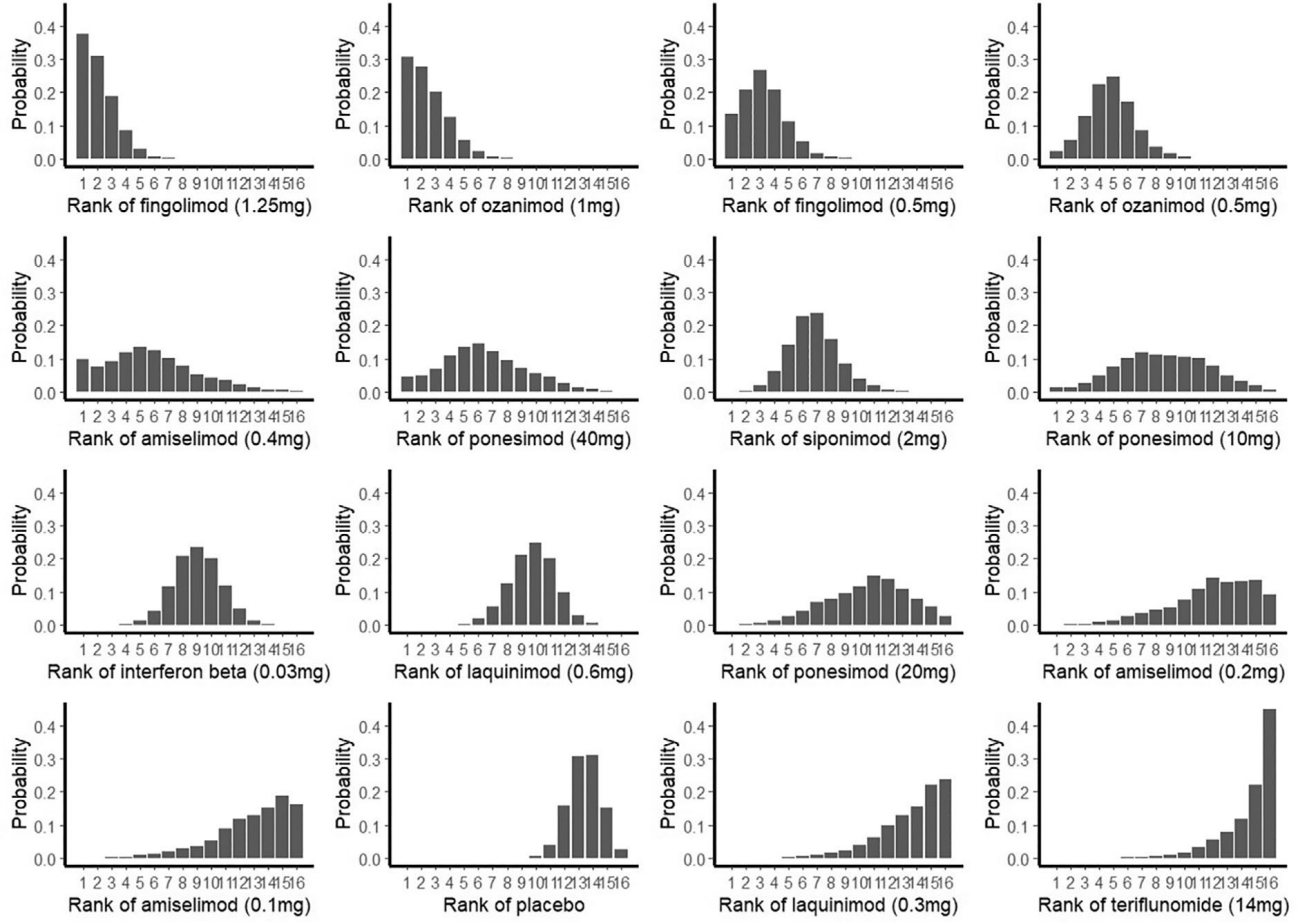
Rank plot (The higher effectiveness of placebo over that of teriflunomide is very likely a structural artifact of the network because it (teriflunomide) had been reported in only one study, while placebo was used as a common comparator in almost all trials. This disproportion between teriflunomide and placebo could amplify the relative efficacy of placebo versus teriflunomide due to a variability in event rates in a much wider set of placebo‐controlled studies).

14 studies with a total of 12,177 participants reported ARR. Fingolimod (1.25 mg) had the highest reduction in ARR (SMD = −0.4422, 95% CI = [−0.5450 to −0.3394], z‐score = −8.43, *p*‐value < 0.0001, I2 = 0%, SUCRA = 92.65%). Ozanimod (1 mg) had the second‐highest reduction in ARR (SMD = −0.4300, 95% CI = [−0.5670 to −0.2931], z‐score = −6.16, *p*‐value < 0.0001, I2 = 0%, SUCRA = 90.30%). Figures 3 and 4 show the forest plot and rank plot. Upon performing node‐split analysis, no inconsistencies were found in annualized relapse rate. The results of this analysis are presented in Figure S2.

#### 3.3.2 New Gd‐Enhanced Lesions From Baseline

Twelve studies with a total of 10,595 participants reported new Gd‐enhanced lesions from baseline. Ozanimod (1 mg) had the lowest number of new lesions as compared to placebo (SMD = −0.6516, 95% CI = [−0.8944 to −0.4087], z‐score = −5.26, *p*‐value < 0.0001, I2 = 60.4%, SUCRA = 86.38%). Fingolimod (5 mg) had the second‐lowest number of new lesions (SMD = −0.5742, 95% CI = [−0.9075 to −0.2410], z‐score = −3.38, *p*‐value = 0.0007, I2 = 60.4%, SUCRA = 73.11%). The league table is given as Table S4. The forest plot and the rank plot are given as Figures S3 and S4, respectively. Meta‐regression was performed to explain the heterogeneity in this outcome. 35.09% of the heterogeneity was explained by the differences in the mean time in years since symptom onset. Two studies [Kappos (2018) and Kappos (2021)] were excluded because they were disconnected from the network. Node‐split analysis revealed no evidence of inconsistency for this outcome. The results of this analysis are presented in Figure S7.

#### 3.3.3 Reduction in Brain Volume

Nine studies involving 9910 participants reported a reduction in brain volume from baseline. Laquinimod (1.2 mg) had the lowest reduction in brain volume as compared to placebo (SMD = −0.3585, 95% CI = [−0.5107 to −0.2062], z‐score = −4.62, *p*‐value < 0.0001, I2 = 41%, SUCRA = 86.3%). Moreover, laquinimod (0.6 mg) had the second‐lowest reduction in brain volume as compared to placebo (SMD = −0.3281, 95% CI = [−0.4444 to −0.2118], z‐score = −5.53, *p*‐value < 0.0001, I2 = 41%, SUCRA = 80.84%). No inconsistencies were detected in the node‐split analysis for reduction in brain volume, as shown in Figure S11. The league table is given as Table S5 and the forest plot, and the rank plot are given as Figures S8 and S9, respectively. Kappos (2021) was removed because it was disconnected from the network.

#### 3.3.4 Proportion of Relapse‐Free Patients

6833/10102 (67.64%) patients from 12 studies were reported to be relapse‐free at the end of the study. Siponimod (2 mg) had the highest odds of achieving relapse‐free status (OR = 7.4667, 95% CI = [2.6046 to 21.4052], z‐score = 3.74, *p*‐value = 0.0002, I2 = 69.6%, SUCRA = 94.41%). Fingolimod (5 mg) had the second‐highest odds of achieving relapse‐free status (OR = 4.5719, 95% CI = [1.6214; 12.8909], z‐score = 2.87, *p*‐value = 0.0041, I2 = 69.6%, SUCRA = 84.07%). The league table is given as Table S6. The rank plot and the forest plot are given as Figures S13 and S14, respectively. Meta‐regression was performed to explain the heterogeneity in this outcome. 35.40% of the heterogeneity was explained by the differences in the mean number of relapses in the last 12 months. Upon performing the node‐splitting analysis, inconsistencies were reported in two comparisons (fingolimod 1.25 mg vs. interferon beta 0.03 mg, *p* = 0.0098, and fingolimod 1.25 mg vs. placebo, *p* = 0.0083). These results are shown in Figure S16.

#### 3.3.5 Adverse Events

13,090/16,006 participants from 17 studies reported adverse events. Siponimod (1.25 mg) had the lowest odds of adverse events (OR = 0.4606, 95% CI = [0.1893 to 1.1205], z‐score = −1.71, *p*‐value = 0.0874, I2 = 15%, SUCRA = 93.2%). Whereas fingolimod (5 mg) had the highest odds (OR = 10.6590, 95% CI = [2.4701 to 45.9949], z‐score = 3.17, *p*‐value = 0.0015, I2 = 15%, SUCRA = 1.38%). The league table and the network plot are given as Table S7 and Figure S17, respectively. Upon performing node‐split analysis, no inconsistencies were observed in adverse events. The results of the analysis are shown in Figure S18.

#### 3.3.6 Serious Adverse Events

1313/14,673 (8.94%) participants from 16 studies reported serious adverse events. Siponimod (0.25 mg) was associated with the least odds of serious adverse events (OR = 0.1584, 95% CI = [0.0083 to 3.0375], z‐score = −1.22, *p*‐value = 0.2214, I2 = 18.3%, SUCRA = 87.84%). Whereas siponimod (0.5 mg) was associated with the highest odds of serious adverse events (OR = 3.9054, 95% CI = [1.1343 to 13.4458], z‐score = 2.16, *p*‐value = 0.0308, I2 = 18.3%, SUCRA = 5.25%). This peculiar result can be explained by the fact that Siponimod 0.25 mg and 0.5 mg are present in only one study (Selmaj, 2013) with almost 50 participants in each group. Siponimod 0.25 mg had 0/51 adverse events, whereas siponimod 0.5 mg had 8/43 serious adverse events [19]. Node‐split analysis demonstrated no signs of inconsistency in serious adverse events, as illustrated in Figure S21. The league table is given as Table S8. The rank plot and the network plot are given as Figures S19 and S20, respectively.

#### 3.3.7 Withdrawal due to Adverse Events

863/15,889 (5.43%) participants discontinued due to adverse events. Siponimod (0.25 mg) had the lowest odds of withdrawal due to adverse events (OR = 0.3354, 95% CI = [0.0348 to 3.2307], z‐score = −0.95, *p*‐value = 0.3445, I2 = 47.7%, SUCRA = 85.88%). Whereas ponesimod (40 mg) had the highest odds of withdrawal due to adverse events (OR = 6.1100, 95% CI = [1.4368 to 25.9837], z‐score = 2.45, *p*‐value = 0.0143, I2 = 47.7% SUCRA = 8.96%). Upon performing the node‐splitting analysis, inconsistency was reported in only one comparison (fingolimod 1.25 mg vs. placebo, *p* = 0.0443), as shown by Figure S25. The league table is given as Table S9. The forest plot and the rank plot are given as Figures S22 and S24, respectively.

### 3.4 Best Treatment

**FIGURE 5:**
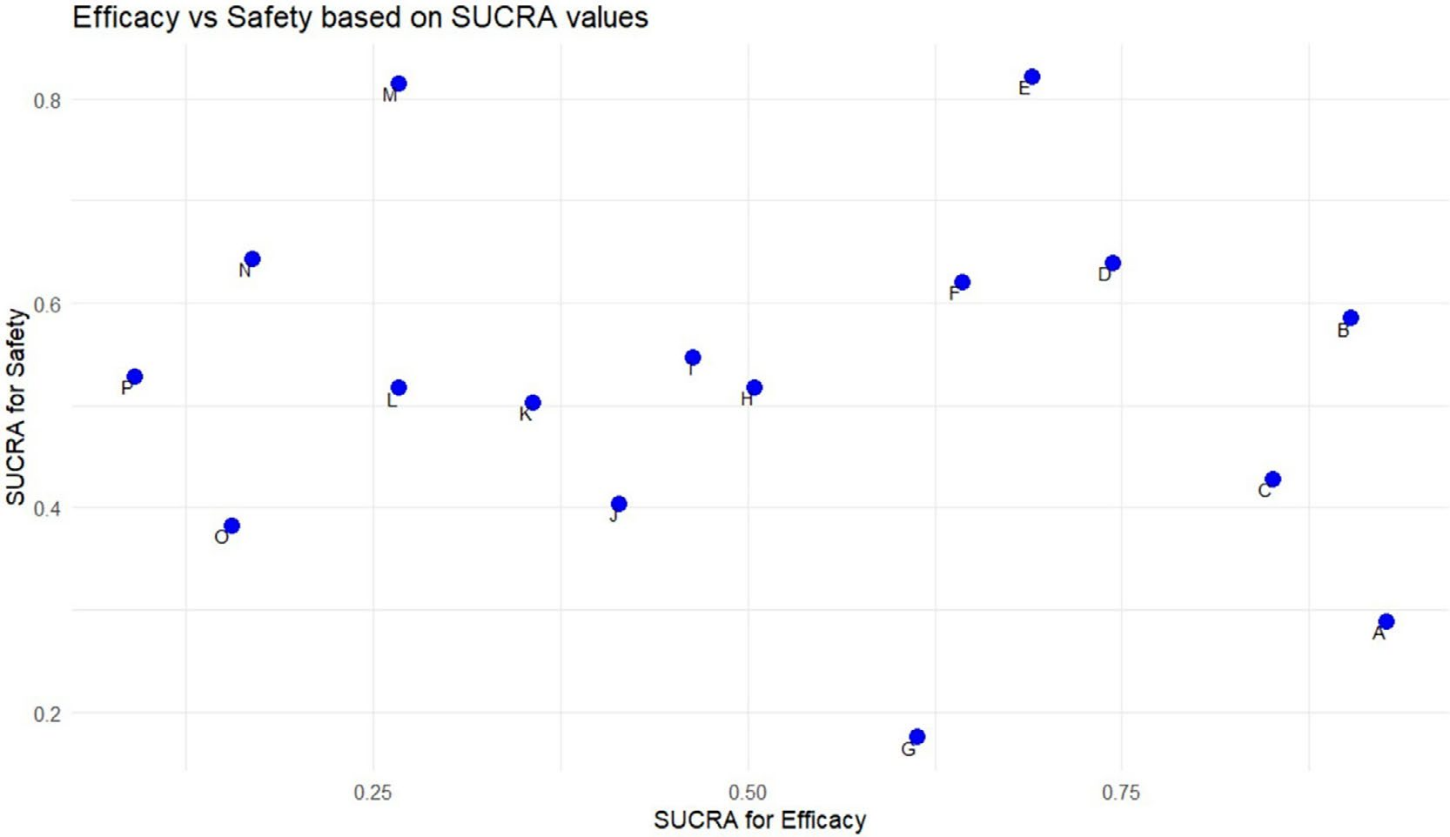
Scatter plot showing the best Treatment (Ameselimod (0.4 mg) had the highest average SUCRA of 75.59%). A: Fingolimod (1.25 mg), B: Ozanimod (1 mg), C: Fingolimod (0.5 mg), D: Ozanimod (0.5 mg), E: Amiselimod (0.4 mg), F: Ponesimod (10 mg), G: Siponimod (2 mg), H: Ponesimod (10 mg), I: Interferon beta (0.03 mg), J: Laquinimod (0.6 mg), K: Ponesimod (20 mg), L: Amiselimod (0.2 mg), M: Amiselimod (0.1 mg), N: Placebo, O: Laquinimod (0.3 mg), P: Teriflunomide (14 mg).

We plotted the SUCRAs for the primary outcome of safety (incidence of adverse events) of all treatments against the SUCRAs for the primary outcome of efficacy (ARR) on a scatter plot. The best treatment was amiselimod (0.4 mg) with an average SUCRA of 75.59%. The scatter plot is shown in Figure 5.

### 3.5 Risk of Bias and Quality Assessment

The Cochrane RoB‐2 method was used to evaluate seventeen studies in a thorough risk of bias‐evaluation. Sixteen of the studies have a low overall risk of bias, with Vollmer (2014) raising some concerns. Overall, the results show a low risk of bias in the included studies. The traffic plot for risk of bias is shown in Figure S22. Overall quality of the evidence was assessed by the GRADE approach for Network Meta‐Analysis. The details of this analysis are attached as a Supporting Information “sphingosine GRADE”. The GRADE analysis showed an overall good quality of evidence with a high confidence rating. Concerns (if any) were present in the domain of imprecision mostly and have been acknowledged as a limitation of our study.

### 3.6 Publication Bias

Since our NMA had more than 10 studies, an Egger's test was performed to assess publication bias, and a funnel plot was made. The results of Egger's test showed there was no significant publication bias (*p* = 0.8229). The funnel plot for publication bias is shown in Figure S21.

## 4 Discussion

This network meta‐analysis gives an updated evaluation of the efficacy and safety of S1P receptor modulators for the treatment of MS. We analyzed seven different S1P antagonists (amiselimod, fingolimod, laquinimod, ozanimod, ponesimod, siponimod and teriflunomide), at different dosages from a total of seventeen RCTs with 16,006 participants.

Sphingosine 1 phosphate receptor 1 (S1PR1) is expressed on the surface of lymphocytes and is used to regulate immune cell trafficking and response through regression from lymph nodes, neuron migration and function, and endothelial permeability [3]. Modulation of physiological systems takes place through G‐protein coupled receptors [3]. Fingolimod, once phosphorylated, acts as a functional antagonist by binding to S1PR1 and causing rapid degradation and internalization; nonselective modulation of S1PR3, S1PP4, S1PR5 can lead to unwanted side effects [2, 3]. Selective S1P receptor modulators include ponesimod, Siponimod, and ozanimod, which work mainly on S1PR1; these theoretically favor efficacy and minimize adverse effects [26].

Both Ozanimod and Fingolimod revealed a significantly lower number of new gadolinium‐enhanced lesions from baseline when compared to interferon beta 1a/placebo. Ozanimod 1 mg had the highest efficacy with a SUCRA of 86.38% with Fingolimod 5 mg following closely with 73.11%. These findings support the fact that these drugs are strong candidates for decreasing the progression of MS and improving long‐term patient outcomes. Kappos et al. reported a statistically significant mean number of gadolinium‐enhanced lesions at 6 months, with Fingolimod 5 mg showing a lesion count of 5.7 compared to 14.8 for placebo [14]. Comi et al. demonstrated that Ozanimod 1 mg is more effective, achieving fewer new lesions with a risk ratio of 0.37 compared with interferon beta 1a [23]. Similarly, Cohen et al. showed a statistically significant lower number of lesions at 24 months, with Ozanimod 1 mg showing a mean of 0.18 compared to 0.37 for interferon beta 1a [10]. These findings highlight the efficacy of reducing lesion formation and effectiveness in the treatment of MS.

In our study, Fingolimod 1.25 mg had the highest reduction in annualized relapse rate (ARR), with ozanimod 1 mg having the second highest; further reinforcing their clinically significant effects in treatment of MS and emphasizing their effectiveness in slowing disease progression. Cohen et al. supports these findings by reporting 0.62 rate ratio of ozanimod 1 mg in comparison to interferon beta 1a [8]; Similarly, Comi et al. reported that patients were 0.52 times as likely to relapse compared to interferon beta 1a [23]. Cohen et al. reported that the ARR was significantly lower in fingolimod 1.25 mg (0.20) compared to the interferon group (0.33) [13]. Kappos et al. reported similar results when comparing fingolimod with a placebo, emphasizing its efficacy [14, 15]. Calabresi et al. reported a statistically significant lower ARR for fingolimod 1.25 mg with a rate ratio of 0.50, indicating a 50% reduction in relapse risk [16]. Furthermore, Saida et al. reported fingolimod 1.25 mg had a lower annualized relapse rate compared to placebo by showing a statistically significant difference of 0.58 [17]. These collective findings reinforce the effectiveness of Ozanimod and Fingolimod in reducing relapse rates. The consistency in results of multiple studies highlights the value of Fingolimod in reducing relapse rates in patients with MS. Its robust efficacy across multiple studies supports its position as a prime treatment option for controlling MS and improving long‐term outcomes.

When evaluating treatments for the ability to reduce brain volume loss, by far the strongest contender was laquinimod, with 1.2 mg and 0.6 mg dosages demonstrating the lowest and second‐lowest rate of reduction in brain volume when compared to placebo, respectively. Comi et al. reported that laquinimod 0.06 mg resulted in a lower percentage of brain volume change (PBVC) (−0.87%) from baseline to 24 months, as compared with patients receiving placebo (−1.30%) [19]. Similarly, Vollmer et al. reported a significant reduction in the number of new brain lesions in the laquinimod 0.06 mg group (0.28%) when compared to the placebo group [20]. The CONCERTO clinical trial supports these findings, showing both laquinimod doses having a mean reduction of −0.4% and placebo having a −0.8% reduction of brain volume [12]. In contrast, Giovannoni et al. reported no significant difference in PBVC when comparing laquinimod 0.06 mg with placebo (adjusted mean difference, 0.016%, *p* = 0.903) [11]. Overall, the data show that laquinimod is a promising treatment for preventing the progression of physical and cognitive disabilities associated with MS, improving long‐term outcomes and quality of life for patients. However, it is important to keep in mind that the higher dose causes an increase in serious cardiovascular events and therefore is less suitable [11, 12].

Siponimod 0.25 mg displayed an excellent safety profile, showing the lowest odds of withdrawal due to adverse effects and being associated with the least likelihood of serious adverse events when ranked with the other treatments. In contrast, despite its great efficacy, fingolimod 5 mg was associated with a less favorable safety profile, ranking the highest in odds of adverse events. Kappos et al. showed that there were statistically significant results when comparing adverse events of Fingolimod 5 mg (94%) with the placebo (82%) [3]. These findings indicate that Siponimod is a good alternate treatment for patients who are unable to tolerate Fingolimod.

The scatter plot to analyze efficacy and safety showed that the leading drug candidate was amiselimod (0.4 mg), supported by a SUCRA score of 75.59%, indicating suitable performance in both aspects. This drug demonstrates great efficacy while remaining a well‐tolerated drug, making it particularly beneficial in cases where patients are experiencing side effects from other treatments. Amiselimod shows promise to be the preferred therapeutic option for MS. Our findings compare well with the previous meta‐analysis; Tong et al. ranked amiselimod highest as well when analyzing safety and efficacy together, making it the most appropriate intervention with minimum side effects [3]. The MOMUMENT core and extension studies showed that amiselimod up to 0.4 mg was well tolerated for patients with RRMS, with no serious infections or malignancies observed or any signal that long‐term use would increase the risk of such adverse events, although relevant monitoring is still recommended during treatment [24, 27]. Improvement in patients' clinical and MRI‐related outcomes supports the importance of this treatment in long‐term control of RRMS progression. However, only a single study was included that evaluated the effectiveness of this drug, highlighting the need for additional long‐term trials to provide more robust and comprehensive evidence. This further research is essential to determine whether amiselimod could emerge as the new preferred treatment option for MS, considering safety, efficacy, and overall benefit to patients over extended periods of time.

The addition of 4 new RCTS from the previous meta‐analysis strengths our study as it provides additional insights into the performance of laquinimod and Ponesimod; as well as incorporating data on an additional drug teriflunomide as a comparator. No heterogeneity was observed in annualized relapse rate, and it was consistently low in both adverse events and serious adverse events, strengthening the robustness of our results and analysis.

Our NMA contains a few limitations, such as majority of the RCTs were conducted in the West, and there could be differences in outcomes when comparing with Eastern and Asian countries. Another limitation inherent to the nature of network meta‐analyses is that these are indirect comparisons of drugs, with no head‐to‐head comparisons in the same clinical trial. This makes it difficult to make robust comparisons of the efficacy and safety of the drugs and limits the strength of the evidence. Inconsistency seen in the outcomes of “patients free from relapse” and “dropout due to adverse events” can cause some bias in the results. Heterogeneity was found in some outcomes, but some were explained by meta‐regression; heterogeneity was observed in withdrawals due to adverse events, which could alter the results. The wide range of follow‐up periods also introduces a potential source of heterogeneity within the analysis. Adverse events can arise due to different mechanisms of action and can therefore influence the reliability of the comparisons. The apparent superior efficacy of placebo over teriflunomide is also a limitation due to the disproportionate representation of placebo in multiple studies compared to teriflunomide in only one. Even though the confidence rating of our evidence is mostly “High”, the GRADE analysis showed some concerns in the domain of “imprecision”, and this is also one limitation of our study. We did not include gray literature in our search strategy, which may lead to publication bias by not including any gray literature/unpublished studies with negative or inconclusive results.

Despite the limitations, the findings from this network meta‐analysis provide valuable insight that can guide clinicals to make informed decisions on the pharmacological treatments for MS. By considering each individual patient's clinical profile, personal preferences and response to prior treatments, clinicians can maximize efficacy and improve patient outcomes.

## 5 Conclusion

Fingolimod (1.25 mg) and ozanimod (1 mg) had the best efficacy, and siponimod (1.25 mg and 0.25 mg) had the best safety profile among the S1PRM. Further longitudinal studies should be conducted to assess the long‐term effects of these drugs on patient‐reported outcomes.

## Author Contributions

**Faizan Shahzad:** conceptualization, methodology, writing – original draft. **Taimoon Rasheed:** formal analysis, writing – original draft. **Momina Riaz Siddiqui:** formal analysis, writing – original draft. **Hamza Hamid:** investigation, resources. **Marwah Bintay Khalid:** data curation, writing – review and editing. **Haroon Shabbir:** writing – review and editing. **Besher Shami:** writing – review and editing, corresponding author. **Abdullah:** writing – review and editing. **Syed Ijlal Ahmed:** writing – review and editing.

## Ethics Statement

Since this is a review article, ethical approval is not required.

## Consent

Since this is a review article of already published data, informed consent is waived.

## Conflicts of Interest

The authors declare no conflicts of interest.

## Supporting information


Data S1.

**File S1.** Sphingosine GRADE.

**Figure S1.** Network ARR.
**Figure S2.** Network split plot ARR.
**Figure S3.** Network plot for Gd lesion.
**Figure S4.** Forest plot GD Lesion.
**Figure S5.** Rank plot GD Lesion.
**Figure S6.** Bubble plot GD lesions.
**Figure S7.** Network split plot GD lesions.
**Figure S8.** Forest plot brain vol.
**Figure S9.** Rank plot Brain vol.
**Figure S10.** Network plot brain vols.
**Figure S11.** Network Split Plot Brain Volume.
**Figure S12.** Network relapse free.
**Figure S13.** Rank plot relapse free.
**Figure S14.** Forest plot relapse free.
**Figure S15.** Bubble plot relapse free.
**Figure S16.** Network split plot relapse free.
**Figure S17.** Network plot for AEs.
**Figure S18.** Network split plot AEs.
**Figure S19.** Rank plot for SAEs.
**Figure S20.** Network plot for SAEs.
**Figure S21.** Network split plot SAEs.
**Figure S22.** Forest plot for DCAE.
**Figure S23.** Network plot for DCAE.
**Figure S24.** Rank plot for DCAEs.
**Figure S25.** Network split plot DCAEs.
**Figure S26.** Forest plot for Adverse Events.
**Figure S27.** Rank plot for Adverse Events.
**Figure S28.** Funnel plot.
**Figure S29.** Traffic plot for risk of bias.
**Table S1.** PICOS table and search strategy.
**Table S2.** Baseline Characteristics.
**Table S3.** League Table ARR.
**Table S4.** League table GD lesion.
**Table S5.** League Table Brain vol.
**Table S6.** League table for relapse free.
**Table S7.** League table AEs.
**Table S8.** League Table for SAEs.
**Table S9.** League table for DCAE.

## Data Availability

The data that support the findings of this study are available from the corresponding author upon reasonable request.
