# Supplementary material for: The Comparative Effectiveness and Tolerability of Sphingosine‐1‐Phosphate Receptor Modulators in Patients With Multiple Sclerosis: A Network Meta‐Analysis of Randomized Controlled Trials
Source: Ann Clin Transl Neurol. 2025 Jul 4;12(10):2002–11. doi: 10.1002/acn3.70122 (PMC12516247; doi:10.1002/acn3.70122)
Supplement: Supplementary file 1 — Data S1. [file ACN3-12-2002-s001.docx]

| Comparison | Number of studies | Within-study bias | Reporting bias | Indirectness | Imprecision | Heterogeneity | Incoherence | Confidence rating | Reason(s) for downgrading |
| --- | --- | --- | --- | --- | --- | --- | --- | --- | --- |
| A (0.1mg):A (0.2mg) | 1 | No concerns | Low risk | No concerns | Major concerns | No concerns | No concerns | High | ["Imprecision"] |
| A (0.1mg):A (0.4mg) | 1 | No concerns | Low risk | No concerns | Major concerns | No concerns | No concerns | High | ["Imprecision"] |
| A (0.1mg):placebo | 1 | No concerns | Low risk | No concerns | Major concerns | No concerns | No concerns | High | ["Imprecision"] |
| A (0.2mg):A (0.4mg) | 1 | No concerns | Low risk | No concerns | Major concerns | No concerns | No concerns | High | ["Imprecision"] |
| A (0.2mg):placebo | 1 | No concerns | Low risk | No concerns | Major concerns | No concerns | No concerns | High | ["Imprecision"] |
| A (0.4mg):placebo | 1 | No concerns | Low risk | No concerns | Major concerns | No concerns | No concerns | High | ["Imprecision"] |
| F (0.5mg):F (1.25mg) | 4 | No concerns | Low risk | No concerns | Major concerns | No concerns | No concerns | High | ["Imprecision"] |
| F (0.5mg):IB (0.03mg) | 1 | No concerns | Low risk | No concerns | No concerns | Major concerns | No concerns | High | ["Heterogeneity"] |
| F (0.5mg):placebo | 3 | No concerns | Low risk | No concerns | Major concerns | No concerns | No concerns | High | ["Imprecision"] |
| F (1.25mg):F (5mg) | 1 | No concerns | Low risk | No concerns | No concerns | No concerns | No concerns | High | [] |
| F (1.25mg):IB (0.03mg) | 1 | No concerns | Low risk | No concerns | Major concerns | No concerns | No concerns | High | ["Imprecision"] |
| F (1.25mg):placebo | 4 | No concerns | Low risk | No concerns | No concerns | No concerns | No concerns | High | [] |
| F (5mg):placebo | 1 | No concerns | Low risk | No concerns | No concerns | No concerns | No concerns | High | [] |
| IB (0.03mg):L (0.6mg) | 1 | No concerns | Low risk | No concerns | No concerns | No concerns | No concerns | High | [] |
| IB (0.03mg):O (0.5mg) | 2 | No concerns | Low risk | No concerns | No concerns | No concerns | No concerns | High | [] |
| IB (0.03mg):O (1mg) | 2 | No concerns | Low risk | No concerns | No concerns | No concerns | No concerns | High | [] |
| IB (0.03mg):placebo | 1 | No concerns | Low risk | No concerns | No concerns | No concerns | No concerns | High | [] |
| L (0.3mg):L (0.6mg) | 1 | No concerns | Low risk | No concerns | Major concerns | No concerns | No concerns | High | ["Imprecision"] |
| L (0.3mg):placebo | 1 | No concerns | Low risk | No concerns | Major concerns | No concerns | No concerns | High | ["Imprecision"] |
| L (0.6mg):L (1.2mg) | 1 | No concerns | Low risk | No concerns | Major concerns | No concerns | No concerns | High | ["Imprecision"] |
| L (0.6mg):L (1.5mg) | 1 | No concerns | Low risk | No concerns | No concerns | No concerns | No concerns | High | [] |
| L (0.6mg):placebo | 5 | No concerns | Low risk | No concerns | No concerns | Major concerns | No concerns | High | ["Heterogeneity"] |
| L (1.2mg):placebo | 1 | No concerns | Low risk | No concerns | No concerns | Major concerns | No concerns | High | ["Heterogeneity"] |
| L (1.5mg):placebo | 1 | No concerns | Low risk | No concerns | No concerns | Major concerns | No concerns | High | ["Heterogeneity"] |
| O (0.5mg):O (1mg) | 2 | No concerns | Low risk | No concerns | Major concerns | No concerns | No concerns | High | ["Imprecision"] |
| P (10mg):P (20mg) | 1 | No concerns | Low risk | No concerns | Major concerns | No concerns | No concerns | High | ["Imprecision"] |
| P (10mg):P (40mg) | 1 | No concerns | Low risk | No concerns | Major concerns | No concerns | No concerns | High | ["Imprecision"] |
| P (10mg):placebo | 1 | No concerns | Low risk | No concerns | Major concerns | No concerns | No concerns | High | ["Imprecision"] |
| P (20mg):P (40mg) | 1 | No concerns | Low risk | No concerns | Major concerns | No concerns | No concerns | High | ["Imprecision"] |
| P (20mg):T (14mg) | 1 | No concerns | Low risk | No concerns | Major concerns | No concerns | No concerns | High | ["Imprecision"] |
| P (20mg):placebo | 1 | No concerns | Low risk | No concerns | Major concerns | No concerns | No concerns | High | ["Imprecision"] |
| P (40mg):placebo | 1 | No concerns | Low risk | No concerns | Major concerns | No concerns | No concerns | High | ["Imprecision"] |
| S (0.25mg):S (0.5mg) | 1 | No concerns | Low risk | No concerns | Major concerns | No concerns | No concerns | High | ["Imprecision"] |
| S (0.25mg):S (1.25mg) | 1 | No concerns | Low risk | No concerns | Major concerns | No concerns | No concerns | High | ["Imprecision"] |
| S (0.25mg):S (10mg) | 1 | No concerns | Low risk | No concerns | No concerns | No concerns | No concerns | High | [] |
| S (0.25mg):S (2mg) | 1 | No concerns | Low risk | No concerns | No concerns | Major concerns | Major concerns | Moderate | ["Heterogeneity","Incoherence"] |
| placebo:S (0.25mg) | 1 | No concerns | Low risk | No concerns | Major concerns | No concerns | No concerns | High | ["Imprecision"] |
| S (0.5mg):S (1.25mg) | 1 | No concerns | Low risk | No concerns | Major concerns | No concerns | No concerns | High | ["Imprecision"] |
| S (0.5mg):S (10mg) | 1 | No concerns | Low risk | No concerns | Major concerns | No concerns | No concerns | High | ["Imprecision"] |
| S (0.5mg):S (2mg) | 1 | No concerns | Low risk | No concerns | Major concerns | No concerns | No concerns | High | ["Imprecision"] |
| placebo:S (0.5mg) | 1 | No concerns | Low risk | No concerns | Major concerns | No concerns | No concerns | High | ["Imprecision"] |
| S (10mg):S (1.25mg) | 1 | No concerns | Low risk | No concerns | No concerns | No concerns | No concerns | High | [] |
| S (1.25mg):S (2mg) | 1 | No concerns | Low risk | No concerns | No concerns | No concerns | Major concerns | High | ["Incoherence"] |
| placebo:S (1.25mg) | 1 | No concerns | Low risk | No concerns | Major concerns | No concerns | Major concerns | Moderate | ["Imprecision","Incoherence"] |
| S (10mg):S (2mg) | 1 | No concerns | Low risk | No concerns | Major concerns | No concerns | Major concerns | Moderate | ["Imprecision","Incoherence"] |
| placebo:S (10mg) | 1 | No concerns | Low risk | No concerns | No concerns | Major concerns | Major concerns | Moderate | ["Heterogeneity","Incoherence"] |
| placebo:S (2mg) | 2 | No concerns | Low risk | No concerns | No concerns | No concerns | No concerns | High | [] |
| A (0.1mg):F (0.5mg) | 0 | No concerns | Low risk | No concerns | Major concerns | No concerns | No concerns | High | ["Imprecision"] |
| A (0.1mg):F (1.25mg) | 0 | No concerns | Low risk | No concerns | No concerns | Major concerns | No concerns | High | ["Heterogeneity"] |
| A (0.1mg):F (5mg) | 0 | No concerns | Low risk | No concerns | No concerns | No concerns | No concerns | High | [] |
| A (0.1mg):IB (0.03mg) | 0 | No concerns | Low risk | No concerns | No concerns | No concerns | No concerns | High | [] |
| A (0.1mg):L (0.3mg) | 0 | No concerns | Low risk | No concerns | Major concerns | No concerns | No concerns | High | ["Imprecision"] |
| A (0.1mg):L (0.6mg) | 0 | No concerns | Low risk | No concerns | Major concerns | No concerns | No concerns | High | ["Imprecision"] |
| A (0.1mg):L (1.2mg) | 0 | No concerns | Low risk | No concerns | Major concerns | No concerns | No concerns | High | ["Imprecision"] |
| A (0.1mg):L (1.5mg) | 0 | No concerns | Low risk | No concerns | Major concerns | No concerns | No concerns | High | ["Imprecision"] |
| A (0.1mg):O (0.5mg) | 0 | No concerns | Low risk | No concerns | Major concerns | No concerns | No concerns | High | ["Imprecision"] |
| A (0.1mg):O (1mg) | 0 | No concerns | Low risk | No concerns | Major concerns | No concerns | No concerns | High | ["Imprecision"] |
| A (0.1mg):P (10mg) | 0 | No concerns | Low risk | No concerns | Major concerns | No concerns | No concerns | High | ["Imprecision"] |
| A (0.1mg):P (20mg) | 0 | No concerns | Low risk | No concerns | Major concerns | No concerns | No concerns | High | ["Imprecision"] |
| A (0.1mg):P (40mg) | 0 | No concerns | Low risk | No concerns | Major concerns | No concerns | No concerns | High | ["Imprecision"] |
| A (0.1mg):S (0.25mg) | 0 | No concerns | Low risk | No concerns | Major concerns | No concerns | No concerns | High | ["Imprecision"] |
| A (0.1mg):S (0.5mg) | 0 | No concerns | Low risk | No concerns | Major concerns | No concerns | No concerns | High | ["Imprecision"] |
| A (0.1mg):S (1.25mg) | 0 | No concerns | Low risk | No concerns | Major concerns | No concerns | No concerns | High | ["Imprecision"] |
| A (0.1mg):S (10mg) | 0 | No concerns | Low risk | No concerns | No concerns | No concerns | No concerns | High | [] |
| A (0.1mg):S (2mg) | 0 | No concerns | Low risk | No concerns | No concerns | No concerns | No concerns | High | [] |
| A (0.1mg):T (14mg) | 0 | No concerns | Low risk | No concerns | Major concerns | No concerns | No concerns | High | ["Imprecision"] |
| A (0.2mg):F (0.5mg) | 0 | No concerns | Low risk | No concerns | Major concerns | No concerns | No concerns | High | ["Imprecision"] |
| A (0.2mg):F (1.25mg) | 0 | No concerns | Low risk | No concerns | Major concerns | No concerns | No concerns | High | ["Imprecision"] |
| A (0.2mg):F (5mg) | 0 | No concerns | Low risk | No concerns | No concerns | No concerns | No concerns | High | [] |
| A (0.2mg):IB (0.03mg) | 0 | No concerns | Low risk | No concerns | Major concerns | No concerns | No concerns | High | ["Imprecision"] |
| A (0.2mg):L (0.3mg) | 0 | No concerns | Low risk | No concerns | Major concerns | No concerns | No concerns | High | ["Imprecision"] |
| A (0.2mg):L (0.6mg) | 0 | No concerns | Low risk | No concerns | Major concerns | No concerns | No concerns | High | ["Imprecision"] |
| A (0.2mg):L (1.2mg) | 0 | No concerns | Low risk | No concerns | Major concerns | No concerns | No concerns | High | ["Imprecision"] |
| A (0.2mg):L (1.5mg) | 0 | No concerns | Low risk | No concerns | Major concerns | No concerns | No concerns | High | ["Imprecision"] |
| A (0.2mg):O (0.5mg) | 0 | No concerns | Low risk | No concerns | Major concerns | No concerns | No concerns | High | ["Imprecision"] |
| A (0.2mg):O (1mg) | 0 | No concerns | Low risk | No concerns | Major concerns | No concerns | No concerns | High | ["Imprecision"] |
| A (0.2mg):P (10mg) | 0 | No concerns | Low risk | No concerns | Major concerns | No concerns | No concerns | High | ["Imprecision"] |
| A (0.2mg):P (20mg) | 0 | No concerns | Low risk | No concerns | Major concerns | No concerns | No concerns | High | ["Imprecision"] |
| A (0.2mg):P (40mg) | 0 | No concerns | Low risk | No concerns | Major concerns | No concerns | No concerns | High | ["Imprecision"] |
| A (0.2mg):S (0.25mg) | 0 | No concerns | Low risk | No concerns | Major concerns | No concerns | No concerns | High | ["Imprecision"] |
| A (0.2mg):S (0.5mg) | 0 | No concerns | Low risk | No concerns | Major concerns | No concerns | No concerns | High | ["Imprecision"] |
| A (0.2mg):S (1.25mg) | 0 | No concerns | Low risk | No concerns | Major concerns | No concerns | No concerns | High | ["Imprecision"] |
| A (0.2mg):S (10mg) | 0 | No concerns | Low risk | No concerns | Major concerns | No concerns | No concerns | High | ["Imprecision"] |
| A (0.2mg):S (2mg) | 0 | No concerns | Low risk | No concerns | Major concerns | No concerns | No concerns | High | ["Imprecision"] |
| A (0.2mg):T (14mg) | 0 | No concerns | Low risk | No concerns | Major concerns | No concerns | No concerns | High | ["Imprecision"] |
| A (0.4mg):F (0.5mg) | 0 | No concerns | Low risk | No concerns | Major concerns | No concerns | No concerns | High | ["Imprecision"] |
| A (0.4mg):F (1.25mg) | 0 | No concerns | Low risk | No concerns | No concerns | Major concerns | No concerns | High | ["Heterogeneity"] |
| A (0.4mg):F (5mg) | 0 | No concerns | Low risk | No concerns | No concerns | No concerns | No concerns | High | [] |
| A (0.4mg):IB (0.03mg) | 0 | No concerns | Low risk | No concerns | No concerns | No concerns | No concerns | High | [] |
| A (0.4mg):L (0.3mg) | 0 | No concerns | Low risk | No concerns | Major concerns | No concerns | No concerns | High | ["Imprecision"] |
| A (0.4mg):L (0.6mg) | 0 | No concerns | Low risk | No concerns | Major concerns | No concerns | No concerns | High | ["Imprecision"] |
| A (0.4mg):L (1.2mg) | 0 | No concerns | Low risk | No concerns | Major concerns | No concerns | No concerns | High | ["Imprecision"] |
| A (0.4mg):L (1.5mg) | 0 | No concerns | Low risk | No concerns | Major concerns | No concerns | No concerns | High | ["Imprecision"] |
| A (0.4mg):O (0.5mg) | 0 | No concerns | Low risk | No concerns | Major concerns | No concerns | No concerns | High | ["Imprecision"] |
| A (0.4mg):O (1mg) | 0 | No concerns | Low risk | No concerns | Major concerns | No concerns | No concerns | High | ["Imprecision"] |
| A (0.4mg):P (10mg) | 0 | No concerns | Low risk | No concerns | Major concerns | No concerns | No concerns | High | ["Imprecision"] |
| A (0.4mg):P (20mg) | 0 | No concerns | Low risk | No concerns | Major concerns | No concerns | No concerns | High | ["Imprecision"] |
| A (0.4mg):P (40mg) | 0 | No concerns | Low risk | No concerns | Major concerns | No concerns | No concerns | High | ["Imprecision"] |
| A (0.4mg):S (0.25mg) | 0 | No concerns | Low risk | No concerns | Major concerns | No concerns | No concerns | High | ["Imprecision"] |
| A (0.4mg):S (0.5mg) | 0 | No concerns | Low risk | No concerns | Major concerns | No concerns | No concerns | High | ["Imprecision"] |
| A (0.4mg):S (1.25mg) | 0 | No concerns | Low risk | No concerns | Major concerns | No concerns | No concerns | High | ["Imprecision"] |
| A (0.4mg):S (10mg) | 0 | No concerns | Low risk | No concerns | No concerns | No concerns | No concerns | High | [] |
| A (0.4mg):S (2mg) | 0 | No concerns | Low risk | No concerns | No concerns | No concerns | No concerns | High | [] |
| A (0.4mg):T (14mg) | 0 | No concerns | Low risk | No concerns | Major concerns | No concerns | No concerns | High | ["Imprecision"] |
| F (0.5mg):F (5mg) | 0 | No concerns | Low risk | No concerns | No concerns | No concerns | No concerns | High | [] |
| F (0.5mg):L (0.3mg) | 0 | No concerns | Low risk | No concerns | Major concerns | No concerns | No concerns | High | ["Imprecision"] |
| F (0.5mg):L (0.6mg) | 0 | No concerns | Low risk | No concerns | Major concerns | No concerns | No concerns | High | ["Imprecision"] |
| F (0.5mg):L (1.2mg) | 0 | No concerns | Low risk | No concerns | Major concerns | No concerns | No concerns | High | ["Imprecision"] |
| F (0.5mg):L (1.5mg) | 0 | No concerns | Low risk | No concerns | No concerns | No concerns | No concerns | High | [] |
| F (0.5mg):O (0.5mg) | 0 | No concerns | Low risk | No concerns | Major concerns | No concerns | No concerns | High | ["Imprecision"] |
| F (0.5mg):O (1mg) | 0 | No concerns | Low risk | No concerns | Major concerns | No concerns | No concerns | High | ["Imprecision"] |
| F (0.5mg):P (10mg) | 0 | No concerns | Low risk | No concerns | Major concerns | No concerns | No concerns | High | ["Imprecision"] |
| F (0.5mg):P (20mg) | 0 | No concerns | Low risk | No concerns | Major concerns | No concerns | No concerns | High | ["Imprecision"] |
| F (0.5mg):P (40mg) | 0 | No concerns | Low risk | No concerns | Major concerns | No concerns | No concerns | High | ["Imprecision"] |
| F (0.5mg):S (0.25mg) | 0 | No concerns | Low risk | No concerns | Major concerns | No concerns | No concerns | High | ["Imprecision"] |
| F (0.5mg):S (0.5mg) | 0 | No concerns | Low risk | No concerns | Major concerns | No concerns | No concerns | High | ["Imprecision"] |
| F (0.5mg):S (1.25mg) | 0 | No concerns | Low risk | No concerns | No concerns | Major concerns | No concerns | High | ["Heterogeneity"] |
| F (0.5mg):S (10mg) | 0 | No concerns | Low risk | No concerns | Major concerns | No concerns | No concerns | High | ["Imprecision"] |
| F (0.5mg):S (2mg) | 0 | No concerns | Low risk | No concerns | Major concerns | No concerns | No concerns | High | ["Imprecision"] |
| F (0.5mg):T (14mg) | 0 | No concerns | Low risk | No concerns | Major concerns | No concerns | No concerns | High | ["Imprecision"] |
| F (1.25mg):L (0.3mg) | 0 | No concerns | Low risk | No concerns | Major concerns | No concerns | No concerns | High | ["Imprecision"] |
| F (1.25mg):L (0.6mg) | 0 | No concerns | Low risk | No concerns | Major concerns | No concerns | No concerns | High | ["Imprecision"] |
| F (1.25mg):L (1.2mg) | 0 | No concerns | Low risk | No concerns | Major concerns | No concerns | No concerns | High | ["Imprecision"] |
| F (1.25mg):L (1.5mg) | 0 | No concerns | Low risk | No concerns | No concerns | No concerns | No concerns | High | [] |
| F (1.25mg):O (0.5mg) | 0 | No concerns | Low risk | No concerns | Major concerns | No concerns | No concerns | High | ["Imprecision"] |
| F (1.25mg):O (1mg) | 0 | No concerns | Low risk | No concerns | Major concerns | No concerns | No concerns | High | ["Imprecision"] |
| F (1.25mg):P (10mg) | 0 | No concerns | Low risk | No concerns | Major concerns | No concerns | No concerns | High | ["Imprecision"] |
| F (1.25mg):P (20mg) | 0 | No concerns | Low risk | No concerns | Major concerns | No concerns | No concerns | High | ["Imprecision"] |
| F (1.25mg):P (40mg) | 0 | No concerns | Low risk | No concerns | Major concerns | No concerns | No concerns | High | ["Imprecision"] |
| F (1.25mg):S (0.25mg) | 0 | No concerns | Low risk | No concerns | Major concerns | No concerns | No concerns | High | ["Imprecision"] |
| F (1.25mg):S (0.5mg) | 0 | No concerns | Low risk | No concerns | Major concerns | No concerns | No concerns | High | ["Imprecision"] |
| F (1.25mg):S (1.25mg) | 0 | No concerns | Low risk | No concerns | No concerns | No concerns | No concerns | High | [] |
| F (1.25mg):S (10mg) | 0 | No concerns | Low risk | No concerns | Major concerns | No concerns | No concerns | High | ["Imprecision"] |
| F (1.25mg):S (2mg) | 0 | No concerns | Low risk | No concerns | Major concerns | No concerns | No concerns | High | ["Imprecision"] |
| F (1.25mg):T (14mg) | 0 | No concerns | Low risk | No concerns | Major concerns | No concerns | No concerns | High | ["Imprecision"] |
| F (5mg):IB (0.03mg) | 0 | No concerns | Low risk | No concerns | No concerns | No concerns | No concerns | High | [] |
| F (5mg):L (0.3mg) | 0 | No concerns | Low risk | No concerns | No concerns | No concerns | No concerns | High | [] |
| F (5mg):L (0.6mg) | 0 | No concerns | Low risk | No concerns | No concerns | No concerns | No concerns | High | [] |
| F (5mg):L (1.2mg) | 0 | No concerns | Low risk | No concerns | No concerns | No concerns | No concerns | High | [] |
| F (5mg):L (1.5mg) | 0 | No concerns | Low risk | No concerns | No concerns | No concerns | No concerns | High | [] |
| F (5mg):O (0.5mg) | 0 | No concerns | Low risk | No concerns | No concerns | No concerns | No concerns | High | [] |
| F (5mg):O (1mg) | 0 | No concerns | Low risk | No concerns | No concerns | No concerns | No concerns | High | [] |
| F (5mg):P (10mg) | 0 | No concerns | Low risk | No concerns | No concerns | No concerns | No concerns | High | [] |
| F (5mg):P (20mg) | 0 | No concerns | Low risk | No concerns | No concerns | No concerns | No concerns | High | [] |
| F (5mg):P (40mg) | 0 | No concerns | Low risk | No concerns | No concerns | No concerns | No concerns | High | [] |
| F (5mg):S (0.25mg) | 0 | No concerns | Low risk | No concerns | No concerns | No concerns | No concerns | High | [] |
| F (5mg):S (0.5mg) | 0 | No concerns | Low risk | No concerns | No concerns | No concerns | No concerns | High | [] |
| F (5mg):S (1.25mg) | 0 | No concerns | Low risk | No concerns | No concerns | No concerns | No concerns | High | [] |
| F (5mg):S (10mg) | 0 | No concerns | Low risk | No concerns | Major concerns | No concerns | No concerns | High | ["Imprecision"] |
| F (5mg):S (2mg) | 0 | No concerns | Low risk | No concerns | No concerns | No concerns | No concerns | High | [] |
| F (5mg):T (14mg) | 0 | No concerns | Low risk | No concerns | No concerns | No concerns | No concerns | High | [] |
| IB (0.03mg):L (0.3mg) | 0 | No concerns | Low risk | No concerns | Major concerns | No concerns | No concerns | High | ["Imprecision"] |
| IB (0.03mg):L (1.2mg) | 0 | No concerns | Low risk | No concerns | No concerns | Major concerns | No concerns | High | ["Heterogeneity"] |
| IB (0.03mg):L (1.5mg) | 0 | No concerns | Low risk | No concerns | No concerns | No concerns | No concerns | High | [] |
| IB (0.03mg):P (10mg) | 0 | No concerns | Low risk | No concerns | Major concerns | No concerns | No concerns | High | ["Imprecision"] |
| IB (0.03mg):P (20mg) | 0 | No concerns | Low risk | No concerns | Major concerns | No concerns | No concerns | High | ["Imprecision"] |
| IB (0.03mg):P (40mg) | 0 | No concerns | Low risk | No concerns | No concerns | Major concerns | No concerns | High | ["Heterogeneity"] |
| IB (0.03mg):S (0.25mg) | 0 | No concerns | Low risk | No concerns | No concerns | No concerns | No concerns | High | [] |
| IB (0.03mg):S (0.5mg) | 0 | No concerns | Low risk | No concerns | Major concerns | No concerns | No concerns | High | ["Imprecision"] |
| IB (0.03mg):S (1.25mg) | 0 | No concerns | Low risk | No concerns | No concerns | No concerns | No concerns | High | [] |
| IB (0.03mg):S (10mg) | 0 | No concerns | Low risk | No concerns | Major concerns | No concerns | No concerns | High | ["Imprecision"] |
| IB (0.03mg):S (2mg) | 0 | No concerns | Low risk | No concerns | Major concerns | No concerns | No concerns | High | ["Imprecision"] |
| IB (0.03mg):T (14mg) | 0 | No concerns | Low risk | No concerns | Major concerns | No concerns | No concerns | High | ["Imprecision"] |
| L (0.3mg):L (1.2mg) | 0 | No concerns | Low risk | No concerns | Major concerns | No concerns | No concerns | High | ["Imprecision"] |
| L (0.3mg):L (1.5mg) | 0 | No concerns | Low risk | No concerns | No concerns | No concerns | No concerns | High | [] |
| L (0.3mg):O (0.5mg) | 0 | No concerns | Low risk | No concerns | Major concerns | No concerns | No concerns | High | ["Imprecision"] |
| L (0.3mg):O (1mg) | 0 | No concerns | Low risk | No concerns | Major concerns | No concerns | No concerns | High | ["Imprecision"] |
| L (0.3mg):P (10mg) | 0 | No concerns | Low risk | No concerns | Major concerns | No concerns | No concerns | High | ["Imprecision"] |
| L (0.3mg):P (20mg) | 0 | No concerns | Low risk | No concerns | Major concerns | No concerns | No concerns | High | ["Imprecision"] |
| L (0.3mg):P (40mg) | 0 | No concerns | Low risk | No concerns | Major concerns | No concerns | No concerns | High | ["Imprecision"] |
| L (0.3mg):S (0.25mg) | 0 | No concerns | Low risk | No concerns | Major concerns | No concerns | No concerns | High | ["Imprecision"] |
| L (0.3mg):S (0.5mg) | 0 | No concerns | Low risk | No concerns | Major concerns | No concerns | No concerns | High | ["Imprecision"] |
| L (0.3mg):S (1.25mg) | 0 | No concerns | Low risk | No concerns | No concerns | No concerns | No concerns | High | [] |
| L (0.3mg):S (10mg) | 0 | No concerns | Low risk | No concerns | Major concerns | No concerns | No concerns | High | ["Imprecision"] |
| L (0.3mg):S (2mg) | 0 | No concerns | Low risk | No concerns | Major concerns | No concerns | No concerns | High | ["Imprecision"] |
| L (0.3mg):T (14mg) | 0 | No concerns | Low risk | No concerns | Major concerns | No concerns | No concerns | High | ["Imprecision"] |
| L (0.6mg):O (0.5mg) | 0 | No concerns | Low risk | No concerns | Major concerns | No concerns | No concerns | High | ["Imprecision"] |
| L (0.6mg):O (1mg) | 0 | No concerns | Low risk | No concerns | Major concerns | No concerns | No concerns | High | ["Imprecision"] |
| L (0.6mg):P (10mg) | 0 | No concerns | Low risk | No concerns | Major concerns | No concerns | No concerns | High | ["Imprecision"] |
| L (0.6mg):P (20mg) | 0 | No concerns | Low risk | No concerns | Major concerns | No concerns | No concerns | High | ["Imprecision"] |
| L (0.6mg):P (40mg) | 0 | No concerns | Low risk | No concerns | Major concerns | No concerns | No concerns | High | ["Imprecision"] |
| L (0.6mg):S (0.25mg) | 0 | No concerns | Low risk | No concerns | Major concerns | No concerns | No concerns | High | ["Imprecision"] |
| L (0.6mg):S (0.5mg) | 0 | No concerns | Low risk | No concerns | Major concerns | No concerns | No concerns | High | ["Imprecision"] |
| L (0.6mg):S (1.25mg) | 0 | No concerns | Low risk | No concerns | No concerns | Major concerns | No concerns | High | ["Heterogeneity"] |
| L (0.6mg):S (10mg) | 0 | No concerns | Low risk | No concerns | Major concerns | No concerns | No concerns | High | ["Imprecision"] |
| L (0.6mg):S (2mg) | 0 | No concerns | Low risk | No concerns | Major concerns | No concerns | No concerns | High | ["Imprecision"] |
| L (0.6mg):T (14mg) | 0 | No concerns | Low risk | No concerns | Major concerns | No concerns | No concerns | High | ["Imprecision"] |
| L (1.2mg):L (1.5mg) | 0 | No concerns | Low risk | No concerns | No concerns | No concerns | No concerns | High | [] |
| L (1.2mg):O (0.5mg) | 0 | No concerns | Low risk | No concerns | Major concerns | No concerns | No concerns | High | ["Imprecision"] |
| L (1.2mg):O (1mg) | 0 | No concerns | Low risk | No concerns | Major concerns | No concerns | No concerns | High | ["Imprecision"] |
| L (1.2mg):P (10mg) | 0 | No concerns | Low risk | No concerns | Major concerns | No concerns | No concerns | High | ["Imprecision"] |
| L (1.2mg):P (20mg) | 0 | No concerns | Low risk | No concerns | Major concerns | No concerns | No concerns | High | ["Imprecision"] |
| L (1.2mg):P (40mg) | 0 | No concerns | Low risk | No concerns | Major concerns | No concerns | No concerns | High | ["Imprecision"] |
| L (1.2mg):S (0.25mg) | 0 | No concerns | Low risk | No concerns | Major concerns | No concerns | No concerns | High | ["Imprecision"] |
| L (1.2mg):S (0.5mg) | 0 | No concerns | Low risk | No concerns | Major concerns | No concerns | No concerns | High | ["Imprecision"] |
| L (1.2mg):S (1.25mg) | 0 | No concerns | Low risk | No concerns | No concerns | Major concerns | No concerns | High | ["Heterogeneity"] |
| L (1.2mg):S (10mg) | 0 | No concerns | Low risk | No concerns | Major concerns | No concerns | No concerns | High | ["Imprecision"] |
| L (1.2mg):S (2mg) | 0 | No concerns | Low risk | No concerns | Major concerns | No concerns | No concerns | High | ["Imprecision"] |
| L (1.2mg):T (14mg) | 0 | No concerns | Low risk | No concerns | Major concerns | No concerns | No concerns | High | ["Imprecision"] |
| L (1.5mg):O (0.5mg) | 0 | No concerns | Low risk | No concerns | Major concerns | No concerns | No concerns | High | ["Imprecision"] |
| L (1.5mg):O (1mg) | 0 | No concerns | Low risk | No concerns | No concerns | Major concerns | No concerns | High | ["Heterogeneity"] |
| L (1.5mg):P (10mg) | 0 | No concerns | Low risk | No concerns | Major concerns | No concerns | No concerns | High | ["Imprecision"] |
| L (1.5mg):P (20mg) | 0 | No concerns | Low risk | No concerns | Major concerns | No concerns | No concerns | High | ["Imprecision"] |
| L (1.5mg):P (40mg) | 0 | No concerns | Low risk | No concerns | Major concerns | No concerns | No concerns | High | ["Imprecision"] |
| L (1.5mg):S (0.25mg) | 0 | No concerns | Low risk | No concerns | Major concerns | No concerns | No concerns | High | ["Imprecision"] |
| L (1.5mg):S (0.5mg) | 0 | No concerns | Low risk | No concerns | Major concerns | No concerns | No concerns | High | ["Imprecision"] |
| L (1.5mg):S (1.25mg) | 0 | No concerns | Low risk | No concerns | Major concerns | No concerns | No concerns | High | ["Imprecision"] |
| L (1.5mg):S (10mg) | 0 | No concerns | Low risk | No concerns | No concerns | No concerns | No concerns | High | [] |
| L (1.5mg):S (2mg) | 0 | No concerns | Low risk | No concerns | No concerns | No concerns | No concerns | High | [] |
| L (1.5mg):T (14mg) | 0 | No concerns | Low risk | No concerns | Major concerns | No concerns | No concerns | High | ["Imprecision"] |
| O (0.5mg):P (10mg) | 0 | No concerns | Low risk | No concerns | Major concerns | No concerns | No concerns | High | ["Imprecision"] |
| O (0.5mg):P (20mg) | 0 | No concerns | Low risk | No concerns | Major concerns | No concerns | No concerns | High | ["Imprecision"] |
| O (0.5mg):P (40mg) | 0 | No concerns | Low risk | No concerns | Major concerns | No concerns | No concerns | High | ["Imprecision"] |
| O (0.5mg):S (0.25mg) | 0 | No concerns | Low risk | No concerns | Major concerns | No concerns | No concerns | High | ["Imprecision"] |
| O (0.5mg):S (0.5mg) | 0 | No concerns | Low risk | No concerns | Major concerns | No concerns | No concerns | High | ["Imprecision"] |
| O (0.5mg):S (1.25mg) | 0 | No concerns | Low risk | No concerns | Major concerns | No concerns | No concerns | High | ["Imprecision"] |
| O (0.5mg):S (10mg) | 0 | No concerns | Low risk | No concerns | No concerns | Major concerns | No concerns | High | ["Heterogeneity"] |
| O (0.5mg):S (2mg) | 0 | No concerns | Low risk | No concerns | No concerns | No concerns | No concerns | High | [] |
| O (0.5mg):T (14mg) | 0 | No concerns | Low risk | No concerns | Major concerns | No concerns | No concerns | High | ["Imprecision"] |
| O (0.5mg):placebo | 0 | No concerns | Low risk | No concerns | Major concerns | No concerns | No concerns | High | ["Imprecision"] |
| O (1mg):P (10mg) | 0 | No concerns | Low risk | No concerns | Major concerns | No concerns | No concerns | High | ["Imprecision"] |
| O (1mg):P (20mg) | 0 | No concerns | Low risk | No concerns | Major concerns | No concerns | No concerns | High | ["Imprecision"] |
| O (1mg):P (40mg) | 0 | No concerns | Low risk | No concerns | Major concerns | No concerns | No concerns | High | ["Imprecision"] |
| O (1mg):S (0.25mg) | 0 | No concerns | Low risk | No concerns | Major concerns | No concerns | No concerns | High | ["Imprecision"] |
| O (1mg):S (0.5mg) | 0 | No concerns | Low risk | No concerns | Major concerns | No concerns | No concerns | High | ["Imprecision"] |
| O (1mg):S (1.25mg) | 0 | No concerns | Low risk | No concerns | Major concerns | No concerns | No concerns | High | ["Imprecision"] |
| O (1mg):S (10mg) | 0 | No concerns | Low risk | No concerns | Major concerns | No concerns | No concerns | High | ["Imprecision"] |
| O (1mg):S (2mg) | 0 | No concerns | Low risk | No concerns | No concerns | Major concerns | No concerns | High | ["Heterogeneity"] |
| O (1mg):T (14mg) | 0 | No concerns | Low risk | No concerns | Major concerns | No concerns | No concerns | High | ["Imprecision"] |
| O (1mg):placebo | 0 | No concerns | Low risk | No concerns | Major concerns | No concerns | No concerns | High | ["Imprecision"] |
| P (10mg):S (0.25mg) | 0 | No concerns | Low risk | No concerns | Major concerns | No concerns | No concerns | High | ["Imprecision"] |
| P (10mg):S (0.5mg) | 0 | No concerns | Low risk | No concerns | Major concerns | No concerns | No concerns | High | ["Imprecision"] |
| P (10mg):S (1.25mg) | 0 | No concerns | Low risk | No concerns | Major concerns | No concerns | No concerns | High | ["Imprecision"] |
| P (10mg):S (10mg) | 0 | No concerns | Low risk | No concerns | Major concerns | No concerns | No concerns | High | ["Imprecision"] |
| P (10mg):S (2mg) | 0 | No concerns | Low risk | No concerns | Major concerns | No concerns | No concerns | High | ["Imprecision"] |
| P (10mg):T (14mg) | 0 | No concerns | Low risk | No concerns | Major concerns | No concerns | No concerns | High | ["Imprecision"] |
| P (20mg):S (0.25mg) | 0 | No concerns | Low risk | No concerns | Major concerns | No concerns | No concerns | High | ["Imprecision"] |
| P (20mg):S (0.5mg) | 0 | No concerns | Low risk | No concerns | Major concerns | No concerns | No concerns | High | ["Imprecision"] |
| P (20mg):S (1.25mg) | 0 | No concerns | Low risk | No concerns | Major concerns | No concerns | No concerns | High | ["Imprecision"] |
| P (20mg):S (10mg) | 0 | No concerns | Low risk | No concerns | Major concerns | No concerns | No concerns | High | ["Imprecision"] |
| P (20mg):S (2mg) | 0 | No concerns | Low risk | No concerns | Major concerns | No concerns | No concerns | High | ["Imprecision"] |
| P (40mg):S (0.25mg) | 0 | No concerns | Low risk | No concerns | Major concerns | No concerns | No concerns | High | ["Imprecision"] |
| P (40mg):S (0.5mg) | 0 | No concerns | Low risk | No concerns | Major concerns | No concerns | No concerns | High | ["Imprecision"] |
| P (40mg):S (1.25mg) | 0 | No concerns | Low risk | No concerns | Major concerns | No concerns | No concerns | High | ["Imprecision"] |
| P (40mg):S (10mg) | 0 | No concerns | Low risk | No concerns | Major concerns | No concerns | No concerns | High | ["Imprecision"] |
| P (40mg):S (2mg) | 0 | No concerns | Low risk | No concerns | Major concerns | No concerns | No concerns | High | ["Imprecision"] |
| P (40mg):T (14mg) | 0 | No concerns | Low risk | No concerns | Major concerns | No concerns | No concerns | High | ["Imprecision"] |
| S (0.25mg):T (14mg) | 0 | No concerns | Low risk | No concerns | Major concerns | No concerns | No concerns | High | ["Imprecision"] |
| S (0.5mg):T (14mg) | 0 | No concerns | Low risk | No concerns | Major concerns | No concerns | No concerns | High | ["Imprecision"] |
| S (1.25mg):T (14mg) | 0 | No concerns | Low risk | No concerns | Major concerns | No concerns | No concerns | High | ["Imprecision"] |
| S (10mg):T (14mg) | 0 | No concerns | Low risk | No concerns | Major concerns | No concerns | No concerns | High | ["Imprecision"] |
| S (2mg):T (14mg) | 0 | No concerns | Low risk | No concerns | Major concerns | No concerns | No concerns | High | ["Imprecision"] |
| placebo:T (14mg) | 0 | No concerns | Low risk | No concerns | Major concerns | No concerns | No concerns | High | ["Imprecision"] |
|  |  |  |  |  |  |  |  |  |  |
|  |  |  |  |  |  |  |  |  |  |

**Supplementary file 3**: Sphingosine GRADE
