# Supplementary material for: The Comparative Effectiveness and Tolerability of Sphingosine‐1‐Phosphate Receptor Modulators in Patients With Multiple Sclerosis: A Network Meta‐Analysis of Randomized Controlled Trials
Source: Ann Clin Transl Neurol. 2025 Jul 4;12(10):2002–11. doi: 10.1002/acn3.70122 (PMC12516247; doi:10.1002/acn3.70122)
Supplement: Supplementary file 3 — Figure S1. Network ARR. Figure S2. Network split plot ARR. Figure S3. Network plot for Gd lesion. Figure S4. Forest plot GD Lesion. Figure S5. Rank plot GD Lesion. Figure S6. Bubble plot GD lesions. Figure S7. Network split plot GD lesions. Figure S8. Forest plot brain vol. Figure S9. Rank plot Brain vol. Figure S10. Network plot brain vols. Figure S11. Network Split Plot Brain Volume. Figure S12. Network relapse free. Figure S13. Rank plot relapse free. Figure S14. Forest plot relapse free. Figure S15. Bubble plot relapse free. Figure S16. Network split plot relapse free. Figure S17. Network plot for AEs. Figure S18. Network split plot AEs. Figure S19. Rank plot for SAEs. Figure S20. Network plot for SAEs. Figure S21. Network split plot SAEs. Figure S22. Forest plot for DCAE. Figure S23. Network plot for DCAE. Figure S24. Rank plot for DCAEs. Figure S25. Network split plot DCAEs. Figure S26. Forest plot for Adverse Events. Figure S27. Rank plot for Adverse Events. Figure S28. Funnel plot. Figure S29. Traffic plot for risk of bias. Table S1. PICOS table and search strategy. Table S2. Baseline Characteristics. Table S3. League Table ARR. Table S4. League table GD lesion. Table S5. League Table Brain vol. Table S6. League table for relapse free. Table S7. League table AEs. Table S8. League Table for SAEs. Table S9. League table for DCAE. [file ACN3-12-2002-s003.docx]

| **Population** | **Intervention** | **Compare** | **Outcome** | **Study type** |
| --- | --- | --- | --- | --- |
| Patients with Multiple Sclerosis | Sphingosine 1 receptor modulator | Placebo |  | RCTs |
| Multiple Sclerosis [MeSH] | Sphingosine-1-Phosphate Receptor [MeSH]  Fingolimod Hydrochloride [MeSH] | Placebos [MeSH] |  |  |
| Keywords for Multiple Sclerosis:   - MS - Relapsing-remitting MS - Relapsing-remitting Multiple Sclerosis - RRMS - Primary progressive MS - Primary progressive Multiple Sclerosis - PPMS - Secondary progressive MS - Secondary Progressive Multiple Sclerosis - SPMS - Progressive Relapsing MS - Progressive Relapsing Multiple Sclerosis - PRMS - Clinically isolated syndrome - CIS - Radiologically isolated syndrome - RIS - Demyelinating disease - Autoimmune disease of the CNS - Autoimmune encephalitis - Central nervous system demyelination - CNS demyelination - Autoimmune demyelinating disease - Multiple Sclerosis, Acute Relapsing - -Acute Relapsing Multiple Sclerosis - -Remitting-Relapsing Multiple Sclerosis - -Multiple Sclerosis, Remitting-Relapsing - -Remitting Relapsing Multiple Sclerosis - -Chronic Progressive Multiple Sclerosis - -Multiple Sclerosis, Progressive Relapsing - -Multiple Sclerosis, Remittent Progressive - -Remittent Progressive Multiple Sclerosis - -Multiple Sclerosis, Primary Progressive - Sclerosis, Disseminated - Disseminated Sclerosis - Multiple Sclerosis, Acute Fulminating | - Fingolimod - 2-Amino-2-(2-(4-octylphenyl)ethyl)-1,3-propanediol hydrochloride - FTY-720 - FTY720 - FTY 720 - Gilenia - Gilenya - Laquinimod - Nerventra - Siponimod - Mayzent - Ozanimod - Zeposia - Amiselimod - MT-1303 - Ponesimod - Ponvory - ACT-128800 - Sphingosine 1-phosphate receptor modulators - S1P receptor modulators - S1P receptor agonists - Sphingosine-1-phosphate receptor agonists - Sphingosine-1-receptor modulators - S1P receptor modulators - S1P1 receptor - S1P1 receptor agonists - S1P1 receptor modulators - Sphingosine-1-phosphate receptor 1 | Keywords:   - Placebo - Placebos - Inactive Substance - Control - Sugar pill - Sham treatment - Active placebo - Inactive placebo |  |  |

**FINAL SEARCH STRATEGY>**

(("Multiple Sclerosis"[MeSH] OR “Multiple Sclerosis, Acute Relapsing” OR “Remitting-Relapsing Multiple Sclerosis” OR “Multiple Sclerosis, Remitting-Relapsing” OR “Remitting Relapsing Multiple Sclerosis” OR “Chronic Progressive Multiple Sclerosis” OR “Multiple Sclerosis, Progressive Relapsing” OR “Multiple Sclerosis, Remittent Progressive” OR “Remittent Progressive Multiple Sclerosis” OR “Multiple Sclerosis, Primary Progressive” OR “Sclerosis, Disseminated” OR “Disseminated Sclerosis” OR “Multiple Sclerosis, Acute Fulminating” OR "MS" OR "Relapsing-remitting MS" OR "Relapsing-remitting Multiple Sclerosis" OR "RRMS" OR "Primary progressive MS" OR "Primary progressive Multiple Sclerosis" OR "PPMS" OR "Secondary progressive MS" OR "Secondary Progressive Multiple Sclerosis" OR "SPMS" OR "Progressive Relapsing MS" OR "Progressive Relapsing Multiple Sclerosis" OR "PRMS" OR "Clinically isolated syndrome" OR "CIS" OR "Radiologically isolated syndrome" OR "RIS" OR "Demyelinating disease" OR "Autoimmune encephalitis" OR "Central nervous system demyelination" OR "CNS demyelination" OR "Autoimmune demyelinating disease")) AND (("Sphingosine 1 Phosphate Receptor"[MeSH] OR "Fingolimod Hydrochloride"[MeSH] OR "Fingolimod" OR "Gilenya" OR “2-Amino-2-(2-(4-octylphenyl)ethyl)-1,3-propanediol hydrochloride” OR “FTY-720” OR “FTY720” OR “FTY 720” OR “Gilenia” OR "Laquinimod" OR "Siponimod" OR "Mayzent" OR "Ozanimod" OR "Zeposia" OR "Amiselimod" OR "MT-1303" OR "Ponesimod" OR "Ponvory" OR "ACT-128800" OR "Sphingosine-1-phosphate receptor modulators" OR "S1P receptor modulators" OR "S1P receptor agonists" OR "Sphingosine-1-phosphate receptor agonists" OR "Sphingosine-1-receptor modulators" OR "S1P receptor modulators" OR "S1P1 receptor" OR "S1P1 receptor agonists" OR "S1P1 receptor modulators" OR "Sphingosine-1-phosphate receptor 1")) AND (("Placebos"[MeSH] OR "Placebo" OR "Placebos" OR "Inactive Substance" OR "Control" OR "Sugar pill" OR "Sham treatment" OR "Active placebo" OR "Inactive placebo"))

Supplementary Table 1: PICOS table and search strategy

| **Name and Year** | **Interventions (with dose)** | **Female, n (%)** | **Age in years Mean (SD)** | **Baseline EDSS Mean (SD)** | **Treatment duration** | **Follow-up time** | **Dropout (%)** |
| --- | --- | --- | --- | --- | --- | --- | --- |
| Cohen (2010) | fingolimod(1.25mg),  fingolimod(0.5mg),  interferon beta-1a(0.030mg) | fingolimod(1.25mg) **n=293 (68.8)**,  fingolimod(0.5mg) **n=282 (65.4)**,  interferon beta-1a(0.030mg) **n=295 (67.8)** | fingolimod(1.25mg) **n=35.8 (8.4)**,  fingolimod(0.5mg) **n=36.7 (8.8)**,  interferon beta-1a(0.030mg) **n=36.0 (8.3)** | fingolimod(1.25mg) **n=2.21 (1.31)**,  fingolimod(0.5mg) **n=2.24 (1.33)**,  interferon beta-1a(0.030mg) **n=2.19 (1.26)** | 12 months | 12 months | 13.08% |
| Kappos (2006) | fingolimod(1.25mg),  fingolimod(5mg),  placebo | fingolimod(1.25mg) **n=70 (75)**,  fingolimod(5mg) **n=65 (71)**,  placebo **n= 61 (66)** | fingolimod(1.25mg) **n=38.0,**  fingolimod(5mg) **n=38.3**,  placebo **n=37.1** | fingolimod(1.25mg) **n=2.7**  fingolimod(5mg) **n=2.5**,  placebo **n=2.6** | 12 months | 12 months | 19.22% |
| Kappos (2010) | fingolimod(1.25mg),  fingolimod(0.5mg),  placebo | fingolimod(1.25mg) **n=295 (68.8)**,  fingolimod(0.5mg) **n=296 (69.6)**,  placebo **n=298 (71.3)** | fingolimod(1.25mg) **n=37.4 (8.9)**,  fingolimod(0.5mg) **n=36.6 (8.8)**,  placebo **n=37.2 (8.6)** | fingolimod(1.25mg) **n=2.4 (1.4)**,  fingolimod(0.5mg) **n=2.3 (1.3)**,  placebo **n=2.5 (1.3)** | 24 months | 24 months | 18.71% |
| Calabresi (2014) | fingolimod(1.25mg),  fingolimod(0.5mg),  placebo | fingolimod(1.25mg) **n=281 (76),**  fingolimod(0.5mg) **n=275 (77)**,  placebo **n=288 (81)** | fingolimod(1.25mg) **n=40·9 (8·9)**,  fingolimod(0.5mg) **n=40·6 (8·4),**  placebo **n=40·1 (8·4)** | fingolimod(1.25mg) **n= 2·5 (1·3)**,  fingolimod(0.5mg) **n= 2·4 (1·3),**  placebo **n= 2·4 (1·3)** | 24 months | 24 months | 28.16% |
| Saida (2012) | fingolimod(1.25mg) fingolimod(0.5mg) placebo | fingolimod(1.25mg) **n=39 (68.4)**,  fingolimod(0.5mg) **n=40 (70.2)**,  placebo **n=39 (68.4)** | fingolimod(1.25mg) **n=36.0 (9.3)**,  fingolimod(0.5mg) **n=35.0 (9.0)**,  placebo **n=35.0 (8.9)** | fingolimod(1.25mg) **n=1.8 (1.7)**,  fingolimod(0.5mg) **n=2.3 (1.9)**,  placebo **n=2.1 (1.7)** | 6 months | 6 months | 14.04% |
| Comi (2008) | laquinimod(0,3mg) laquinimod(0.6mg) placebo | N/R | N/R | laquinimod(0,3mg) **n=2·3(1·1)**,  laquinimod(0.6mg) **n=2·3 (1·0)**,  placebo **n=2·5 (1·1)** | 36 weeks | 36 weeks | 7.52% |
| Comi (2012) | laquinimod(0.6mg) placebo | laquinimod(0.6mg) **n=391 (71.1)**,  placebo **n=368 (66.2)** | laquinimod(0.6mg) **n=38.9 (9.2)**,  placebo **n=38.5 (9.1)** | laquinimod(0.6mg) **n=2.6 (1.3)**,  placebo **n=2.6 (1.3)** | 24 months | 24 months | 21.88% |
| Vollmer (2014) | laquinimod(0.6mg),  interferon beta-1a(0.03mg) placebo | laquinimod(0.6mg) n=282 (65.0 ),  interferon beta-1a(0.03mg) n=307 (68.7 ),  placebo n=321 (71.3 ) | Median (P25, P75) given: laquinimod(0.6mg) n=36.7 (29.6, 44.0)  interferon beta-1a(0.03mg) n=38.5 (30.3, 45.9),  placebo n=37.5 (30.3, 45.4) | Median (P25, P75) given: laquinimod(0.6mg) n=2.5 (1.5, 3.5),  interferon beta-1a(0.03mg) n=2.5 (1.5, 3.5),  placebo n=2.5 (1.5, 3.5) | 24 months | 24 months | 18.12% |
| Selmaj (2013) | Siponimod(10mg),  Siponimod(2mg),  Siponimod (1·25mg),  Siponimod (0·5mg),  Siponimod (0·25mg),  Placebo | Siponimod(10mg) **n=30 (60)**,  Siponimod(2mg) **n=34 (69)**,  Siponimod (1·25mg) **n= 31 (74)**,  Siponimod (0·5mg) **n=30 (70)**,  Siponimod (0·25mg) **n=42 (82)**,  Placebo **n=45 (73)** | Siponimod(10mg) **n=36·4 (8·4)**,  Siponimod(2mg) **n= 37·4 (8·9)**,  Siponimod (1·25mg) **n= 35·4 (8·9)**,  Siponimod (0·5mg) **n=36·0 (8·8)**,  Siponimod (0·25mg) **37·4 (8·4)**,  Placebo n=**35·4 (8·6)** | Siponimod(10mg) **n=2·3 (1·0)**,  Siponimod(2mg) **n= 2·4 (1·2)**,  Siponimod (1·25mg) **n= 2·0 (1·0)**,  Siponimod (0·5mg) **n=2·2 (1·3)**,  Siponimod (0·25mg) **2·3 (1·1)**,  Placebo n=**2·3 (1·1)** | siponimod 10 mg, 2 mg, or 0·5 mg **(6 months)** siponimod 1·25 mg, 0·25 mg **(3 months)** | siponimod 10 mg, 2 mg, or 0·5 mg **(6 months)** siponimod 1·25 mg, 0·25 mg **(3 months)** | 11.45% |
| Kappos (2018) | siponimod(2mg),  placebo | siponimod(2mg) **n=669 (61)**,  placebo **n=323 (59)** | siponimod(2mg)**n=48·0 (7·8)**,  placebo **n=48·1 (7·9)** | siponimod(2mg)**n=5·4 (1·1)**,  placebo **n=5·4 (1·0)** | 24 months | 24 months | 19.32% |
| Comi (2019) | ozanimod(1mg),  ozanimod(0.5mg),  interferon beta-1a(0.30mg) | ozanimod(1mg) **n=283 (63·3%)** ozanimod(0.5mg) **n=311 (69·0%)** interferon beta-1a(0.30mg) **n=300 (67·0%)** | ozanimod(1mg) **n= 34·8 (9·2)**,  ozanimod(0.5mg) **n=36·0 (9·4)**,  interferon beta-1a(0.30mg) **n=35·9 (9·1)** | ozanimod(1mg) **n=2·6 (1·2)**,  ozanimod(0.5mg) **n=2·7 (1·1)**, interferon beta-1a(0.30mg) **n=2·6 (1·1)** | 12 months | 12 months | 6.76% |
| Kapoos (2016) | amiselimod(0.1mg),  amiselimod(0.2mg),  amiselimod(0.4mg), placebo | amiselimod(0.1mg) **n=69 (66%)**,  amiselimod(0.2mg) **n= 73 (71%)**,  amiselimod(0.4mg) **n= 72 (69%)**,  placebo **n=67 (65%)** | amiselimod(0.1mg) **n=37·2 (9·4)**,  amiselimod(0.2mg) **n= 38·0 (9·6)**,  amiselimod(0.4mg) **n= 37·6 (8·7),**  placebo **n=37·2 (8·5)** | amiselimod(0.1mg) **n= 2·9 (1·3)**,  amiselimod(0.2mg) **n= 2·8 (1·3)**,  amiselimod(0.4mg) **n= 2·6 (1·3),**  placebo **n=2·7 (1·3)** | 24 weeks | 24 weeks | 8.19% |
| Olsson (2014) | ponesimod(10mg),  ponesimod(20mg),  ponesimod(40mg),  placebo | ponesimod(10mg) **n=71 (65.7)**,  ponesimod(20mg) **n=77 (67.5)**,  ponesimod(40mg) **n=79 (66.4)**,  placebo **n=85 (70.2)** | ponesimod(10mg) **n=36.9 (9.2),**  ponesimod(20mg) **n=35.5 (8.5)**,  ponesimod(40mg) **n=36.5 (8.5)**,  placebo **n=36.6 (8.6)** | ponesimod(10mg) **n=2.4 (1.25),**  ponesimod(20mg) **n=2.2 (1.31)**,  ponesimod(40mg) **n=2.2 (1.17)**,  placebo **n=2.2 (1.23)** | 24 weeks | 24 weeks | 14.87% |
| Giovannoni (2020) | laquinimod(0.6mg),  laquinimod(1.5mg), (1.5mg arm discontinued due to AE) placebo | laquinimod(0.6mg) **n=57 (41)** laquinimod(1.5mg) **n=45 (47)** placebo **n=67 (48)** | "laquinimod (0.6mg) **n=46.1 (6.7)** laquinimod (1.5mg) **n=46.1 (7.2)**  placebo **n=46.6 (7.2)** | laquinimod(0.6mg) **n=4.5 (1.0)** laquinimod(1.5mg) **n=4.4 (1.0)** placebo **n=4.5 (0.9)** | 48 weeks | 48 weeks | 46% |
| Cohen (2019) | ozanimod(1mg),  ozanimod(0·5mg),  interferon beta-1a(30 μg/0.03mg) | ozanimod(1mg) **n=291 (67·2%)** ozanimod(0·5mg) **n=287 (65·4%)** interferon beta-1a(30 μg/0.03mg) **n=304 (68·9%)** | ozanimod(1mg) **n=36·0 (8·9)** ozanimod(0·5mg) **n=35·4 (8·8)** interferon beta-1a(30 μg/0.03mg) **n=35·1 (9·1)** | ozanimod(1mg) **n=2·6 (1·15)** ozanimod(0·5mg) **n=2·5 (1·17)** interferon beta-1a(30 μg/0.03mg) **n=2·5 (1·16)** | 24-month | 24-month | 13.80% |
| Kappos (2021) | ponesimod(20mg)  teriflunomide(14mg) | ponesimod(20mg) **n=363 (64.0)** teriflunomide(14mg) **n=372 (65.7)** | ponesimod(20mg) **n=36.7 (8.74)** teriflunomide(14mg) **n=36.8 (8.74)** | ponesimod(20mg) **n=2.57 (1.17)** teriflunomide(14mg) **n=2.56 (1.23)** | 24 months | 24 months + 30 days | 13.06% |
| Comi (2022) | laquinimod(0.6mg)  laquinimod(1.2mg) placebo | laquinimod(0.6mg) **n=510 (70.2%)** laquinimod(1.2mg) **n=475 (64.9%)** placebo **n=488 (65.9%)** | laquinimod(0.6mg) **n=36.8(9.25)** laquinimod(1.2mg) **n=36.1(9.22)** placebo **n=35.9(8.95)** | laquinimod(0.6mg) **(<=4.0)=635 (87.3%), (>4.0)=90 (12.4%), (Missing)=2(0.3%)** laquinimod(1.2mg)**(<=4.0)=644 (88.0%), (>4.0)=88*12.0%)** placebo**(<=4.0)=653 (88.2%), (>4.0)=871 (1.8%)** | 24 months | 24 months | 20.37% |

Supplementary Table 2: Baseline Characteristics


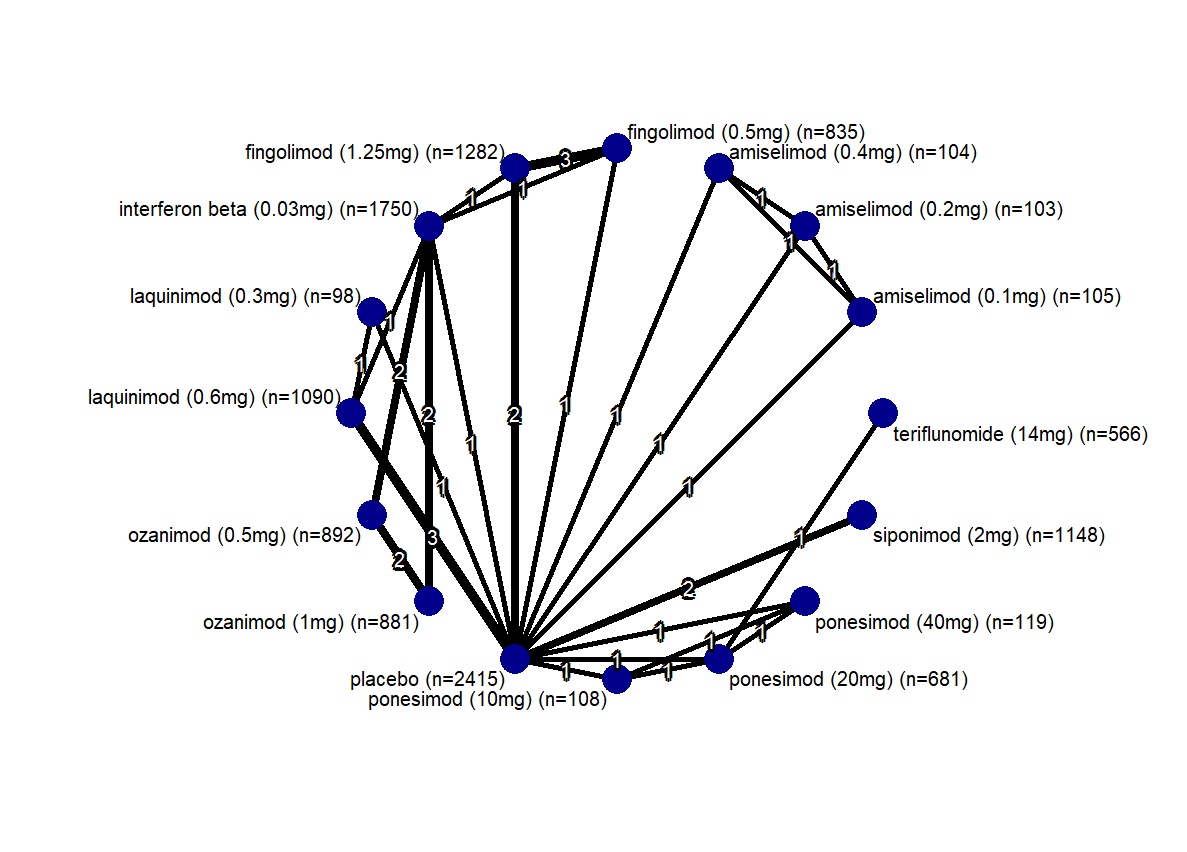


Supplementary Figure: 1 Network ARR


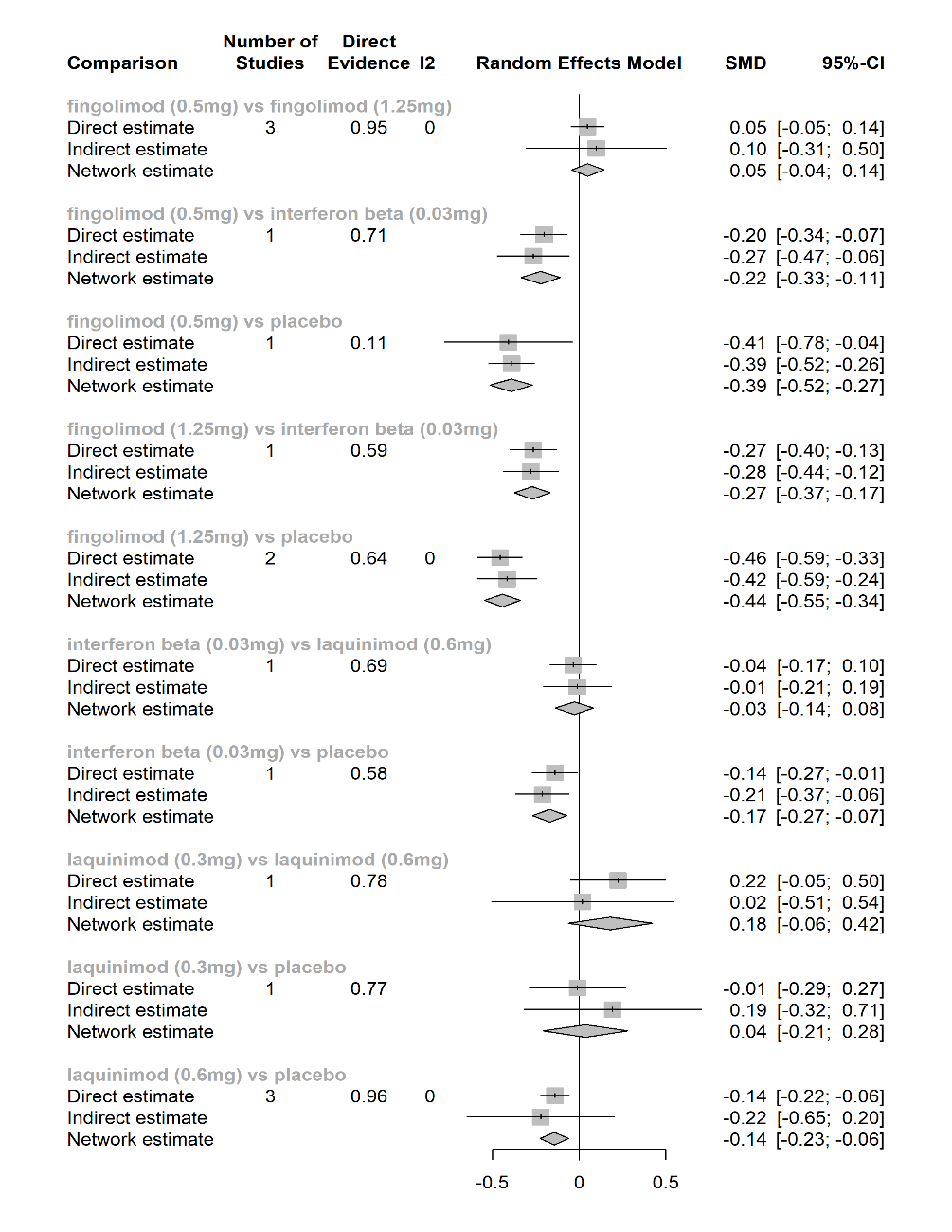


Supplementary Figure 2: Network split plot ARR

| amiselimod (0.1mg) | 0.04 [-0.23; 0.32] | 0.30 [ 0.03; 0.57] | . | . | . | . | . | . | . | 0.00 [-0.27; 0.27] | . | . | . | . | . |
| --- | --- | --- | --- | --- | --- | --- | --- | --- | --- | --- | --- | --- | --- | --- | --- |
| 0.04 [-0.23; 0.32] | amiselimod (0.2mg) | 0.26 [-0.02; 0.53] | . | . | . | . | . | . | . | -0.04 [-0.32; 0.23] | . | . | . | . | . |
| 0.30 [ 0.03; 0.57] | 0.26 [-0.02; 0.53] | amiselimod (0.4mg) | . | . | . | . | . | . | . | -0.30 [-0.57; -0.03] | . | . | . | . | . |
| 0.39 [ 0.09; 0.69] | 0.35 [ 0.05; 0.65] | 0.09 [-0.21; 0.39] | fingolimod (0.5mg) | 0.05 [-0.05; 0.14] | -0.20 [-0.34; -0.07] | . | . | . | . | -0.41 [-0.78; -0.04] | . | . | . | . | . |
| 0.44 [ 0.15; 0.73] | 0.40 [ 0.11; 0.69] | 0.14 [-0.15; 0.43] | 0.05 [-0.04; 0.14] | fingolimod (1.25mg) | -0.27 [-0.40; -0.13] | . | . | . | . | -0.46 [-0.59; -0.33] | . | . | . | . | . |
| 0.17 [-0.12; 0.46] | 0.13 [-0.16; 0.42] | -0.13 [-0.42; 0.16] | -0.22 [-0.33; -0.11] | -0.27 [-0.37; -0.17] | interferon beta (0.03mg) | . | -0.04 [-0.17; 0.10] | 0.15 [ 0.06; 0.25] | 0.26 [ 0.17; 0.35] | -0.14 [-0.27; -0.01] | . | . | . | . | . |
| -0.04 [-0.40; 0.33] | -0.08 [-0.45; 0.29] | -0.34 [-0.70; 0.03] | -0.43 [-0.70; -0.16] | -0.48 [-0.74; -0.22] | -0.21 [-0.47; 0.05] | laquinimod (0.3mg) | 0.22 [-0.05; 0.50] | . | . | -0.01 [-0.29; 0.27] | . | . | . | . | . |
| 0.14 [-0.14; 0.43] | 0.10 [-0.19; 0.38] | -0.16 [-0.44; 0.13] | -0.25 [-0.39; -0.11] | -0.30 [-0.42; -0.18] | -0.03 [-0.14; 0.08] | 0.18 [-0.06; 0.42] | laquinimod (0.6mg) | . | . | -0.14 [-0.22; -0.06] | . | . | . | . | . |
| 0.33 [ 0.02; 0.63] | 0.28 [-0.02; 0.59] | 0.02 [-0.28; 0.33] | -0.07 [-0.21; 0.08] | -0.12 [-0.26; 0.02] | 0.15 [ 0.06; 0.25] | 0.36 [ 0.09; 0.64] | 0.18 [ 0.04; 0.33] | ozanimod (0.5mg) | 0.10 [ 0.01; 0.20] | . | . | . | . | . | . |
| 0.43 [ 0.13; 0.73] | 0.39 [ 0.08; 0.69] | 0.13 [-0.18; 0.43] | 0.04 [-0.11; 0.18] | -0.01 [-0.15; 0.13] | 0.26 [ 0.17; 0.35] | 0.47 [ 0.19; 0.74] | 0.29 [ 0.14; 0.43] | 0.10 [ 0.01; 0.20] | ozanimod (1mg) | . | . | . | . | . | . |
| 0.00 [-0.27; 0.27] | -0.04 [-0.32; 0.23] | -0.30 [-0.57; -0.03] | -0.39 [-0.52; -0.27] | -0.44 [-0.55; -0.34] | -0.17 [-0.27; -0.07] | 0.04 [-0.21; 0.28] | -0.14 [-0.23; -0.06] | -0.33 [-0.46; -0.19] | -0.43 [-0.57; -0.29] | placebo | 0.19 [-0.07; 0.45] | 0.11 [-0.15; 0.36] | 0.27 [ 0.02; 0.52] | 0.25 [ 0.15; 0.35] | . |
| 0.19 [-0.19; 0.57] | 0.15 [-0.23; 0.52] | -0.11 [-0.49; 0.27] | -0.20 [-0.49; 0.09] | -0.25 [-0.53; 0.03] | 0.02 [-0.26; 0.30] | 0.23 [-0.13; 0.58] | 0.05 [-0.22; 0.32] | -0.13 [-0.43; 0.16] | -0.24 [-0.53; 0.05] | 0.19 [-0.07; 0.45] | ponesimod (10mg) | -0.08 [-0.35; 0.18] | 0.08 [-0.18; 0.34] | . | . |
| 0.11 [-0.27; 0.48] | 0.06 [-0.31; 0.44] | -0.19 [-0.57; 0.18] | -0.29 [-0.57; 0.00] | -0.34 [-0.61; -0.06] | -0.06 [-0.34; 0.21] | 0.14 [-0.21; 0.50] | -0.04 [-0.31; 0.23] | -0.22 [-0.51; 0.07] | -0.32 [-0.61; -0.03] | 0.11 [-0.15; 0.36] | -0.08 [-0.35; 0.18] | ponesimod (20mg) | 0.16 [-0.09; 0.42] | . | -0.21 [-0.32; -0.09] |
| 0.27 [-0.10; 0.64] | 0.23 [-0.15; 0.60] | -0.03 [-0.40; 0.34] | -0.12 [-0.40; 0.16] | -0.17 [-0.44; 0.10] | 0.10 [-0.17; 0.37] | 0.31 [-0.04; 0.66] | 0.13 [-0.14; 0.39] | -0.05 [-0.34; 0.23] | -0.16 [-0.45; 0.13] | 0.27 [ 0.02; 0.52] | 0.08 [-0.18; 0.34] | 0.16 [-0.09; 0.42] | ponesimod (40mg) | . | . |
| 0.25 [-0.04; 0.54] | 0.20 [-0.09; 0.50] | -0.05 [-0.34; 0.24] | -0.14 [-0.30; 0.02] | -0.19 [-0.34; -0.05] | 0.08 [-0.06; 0.22] | 0.29 [ 0.02; 0.55] | 0.11 [-0.02; 0.23] | -0.08 [-0.25; 0.09] | -0.18 [-0.35; -0.01] | 0.25 [ 0.15; 0.35] | 0.06 [-0.22; 0.34] | 0.14 [-0.13; 0.42] | -0.02 [-0.29; 0.25] | siponimod (2mg) | . |
| -0.10 [-0.49; 0.29] | -0.14 [-0.54; 0.25] | -0.40 [-0.79; -0.01] | -0.49 [-0.80; -0.18] | -0.54 [-0.84; -0.24] | -0.27 [-0.57; 0.03] | -0.06 [-0.44; 0.31] | -0.24 [-0.54; 0.05] | -0.42 [-0.74; -0.11] | -0.53 [-0.84; -0.22] | -0.10 [-0.38; 0.18] | -0.29 [-0.58; 0.00] | -0.21 [-0.32; -0.09] | -0.37 [-0.65; -0.09] | -0.35 [-0.65; -0.05] | teriflunomide (14mg) |

Supplementary Table 3: League Table ARR

| amiselimod (0.1mg) | 0.26 [-0.08; 0.60] | 0.31 [-0.03; 0.65] | . | . | . | . | . | . | . | . | -0.23 [-0.57; 0.11] | . |
| --- | --- | --- | --- | --- | --- | --- | --- | --- | --- | --- | --- | --- |
| 0.26 [-0.08; 0.60] | amiselimod (0.2mg) | 0.05 [-0.29; 0.39] | . | . | . | . | . | . | . | . | -0.49 [-0.83; -0.15] | . |
| 0.31 [-0.03; 0.65] | 0.05 [-0.29; 0.39] | amiselimod (0.4mg) | . | . | . | . | . | . | . | . | -0.54 [-0.88; -0.20] | . |
| 0.28 [-0.09; 0.65] | 0.02 [-0.35; 0.39] | -0.03 [-0.40; 0.35] | fingolimod (0.5mg) | 0.07 [-0.08; 0.21] | . | -0.22 [-0.47; 0.03] | . | . | . | . | -0.52 [-0.69; -0.35] | . |
| 0.33 [-0.04; 0.70] | 0.07 [-0.30; 0.44] | 0.02 [-0.35; 0.39] | 0.05 [-0.09; 0.19] | fingolimod (1.25mg) | 0.13 [-0.24; 0.51] | -0.29 [-0.55; -0.04] | . | . | . | . | -0.54 [-0.69; -0.38] | . |
| 0.35 [-0.13; 0.82] | 0.08 [-0.40; 0.56] | 0.03 [-0.44; 0.51] | 0.06 [-0.28; 0.41] | 0.02 [-0.32; 0.35] | fingolimod (5mg) | . | . | . | . | . | -0.45 [-0.83; -0.08] | . |
| 0.10 [-0.28; 0.48] | -0.16 [-0.55; 0.22] | -0.21 [-0.60; 0.17] | -0.18 [-0.37; 0.00] | -0.23 [-0.41; -0.05] | -0.25 [-0.61; 0.12] | interferon beta (0.03mg) | . | -0.23 [-0.48; 0.01] | 0.16 [-0.02; 0.33] | 0.32 [ 0.15; 0.50] | -0.36 [-0.60; -0.11] | . |
| -0.19 [-0.65; 0.28] | -0.45 [-0.91; 0.02] | -0.50 [-0.96; -0.03] | -0.47 [-0.81; -0.13] | -0.51 [-0.85; -0.18] | -0.53 [-0.98; -0.08] | -0.28 [-0.63; 0.06] | laquinimod (0.3mg) | 0.15 [-0.19; 0.50] | . | . | -0.08 [-0.43; 0.26] | . |
| -0.07 [-0.44; 0.30] | -0.33 [-0.71; 0.04] | -0.38 [-0.76; -0.01] | -0.35 [-0.55; -0.15] | -0.40 [-0.60; -0.20] | -0.42 [-0.78; -0.05] | -0.17 [-0.36; 0.03] | 0.11 [-0.19; 0.42] | laquinimod (0.6mg) | . | . | -0.17 [-0.32; -0.02] | . |
| 0.25 [-0.16; 0.67] | -0.01 [-0.43; 0.41] | -0.06 [-0.48; 0.36] | -0.03 [-0.28; 0.23] | -0.08 [-0.33; 0.18] | -0.09 [-0.49; 0.31] | 0.16 [-0.02; 0.33] | 0.44 [ 0.06; 0.82] | 0.33 [ 0.06; 0.59] | ozanimod (0.5mg) | 0.17 [-0.01; 0.34] | . | . |
| 0.42 [ 0.00; 0.84] | 0.16 [-0.26; 0.58] | 0.11 [-0.31; 0.53] | 0.14 [-0.11; 0.39] | 0.09 [-0.16; 0.34] | 0.08 [-0.33; 0.48] | 0.32 [ 0.15; 0.50] | 0.61 [ 0.22; 0.99] | 0.49 [ 0.23; 0.75] | 0.17 [-0.01; 0.34] | ozanimod (1mg) | . | . |
| -0.23 [-0.57; 0.11] | -0.49 [-0.83; -0.15] | -0.54 [-0.88; -0.20] | -0.51 [-0.66; -0.36] | -0.56 [-0.70; -0.42] | -0.57 [-0.91; -0.24] | -0.33 [-0.50; -0.16] | -0.04 [-0.35; 0.26] | -0.16 [-0.31; -0.01] | -0.48 [-0.73; -0.24] | -0.65 [-0.89; -0.41] | placebo | 0.54 [ 0.30; 0.77] |
| 0.31 [-0.11; 0.72] | 0.05 [-0.37; 0.46] | -0.00 [-0.42; 0.41] | 0.03 [-0.25; 0.30] | -0.02 [-0.30; 0.25] | -0.04 [-0.45; 0.37] | 0.21 [-0.08; 0.50] | 0.49 [ 0.10; 0.88] | 0.38 [ 0.10; 0.65] | 0.05 [-0.28; 0.39] | -0.12 [-0.45; 0.22] | 0.54 [ 0.30; 0.77] | siponimod (2mg) |

Supplementary Table 4: League table GD lesion


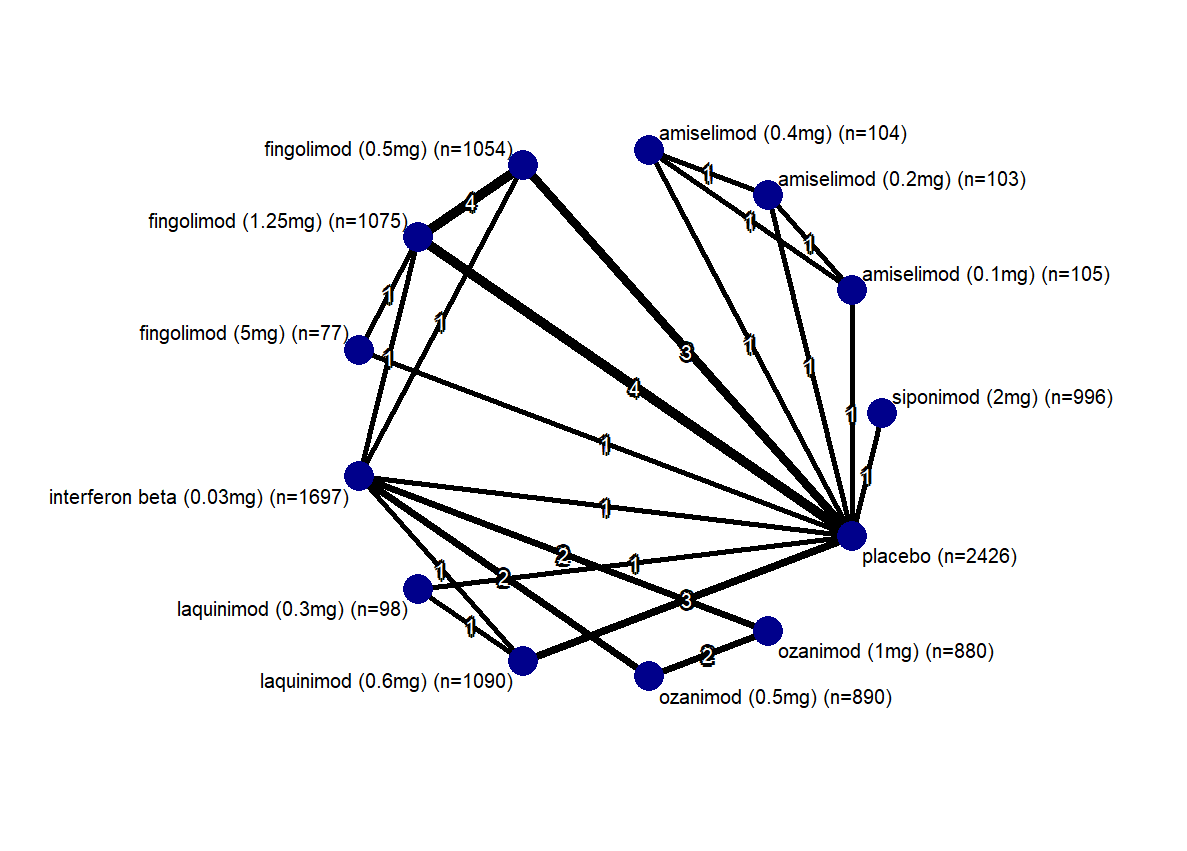


Supplementary Figure 3: Network plot for Gd lesion


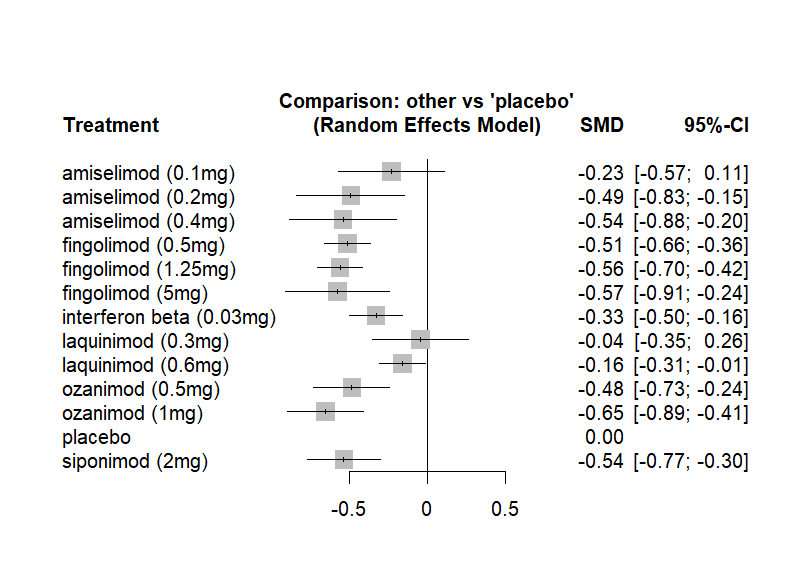


Supplementary Figure 4: Forest plot GD Lesion


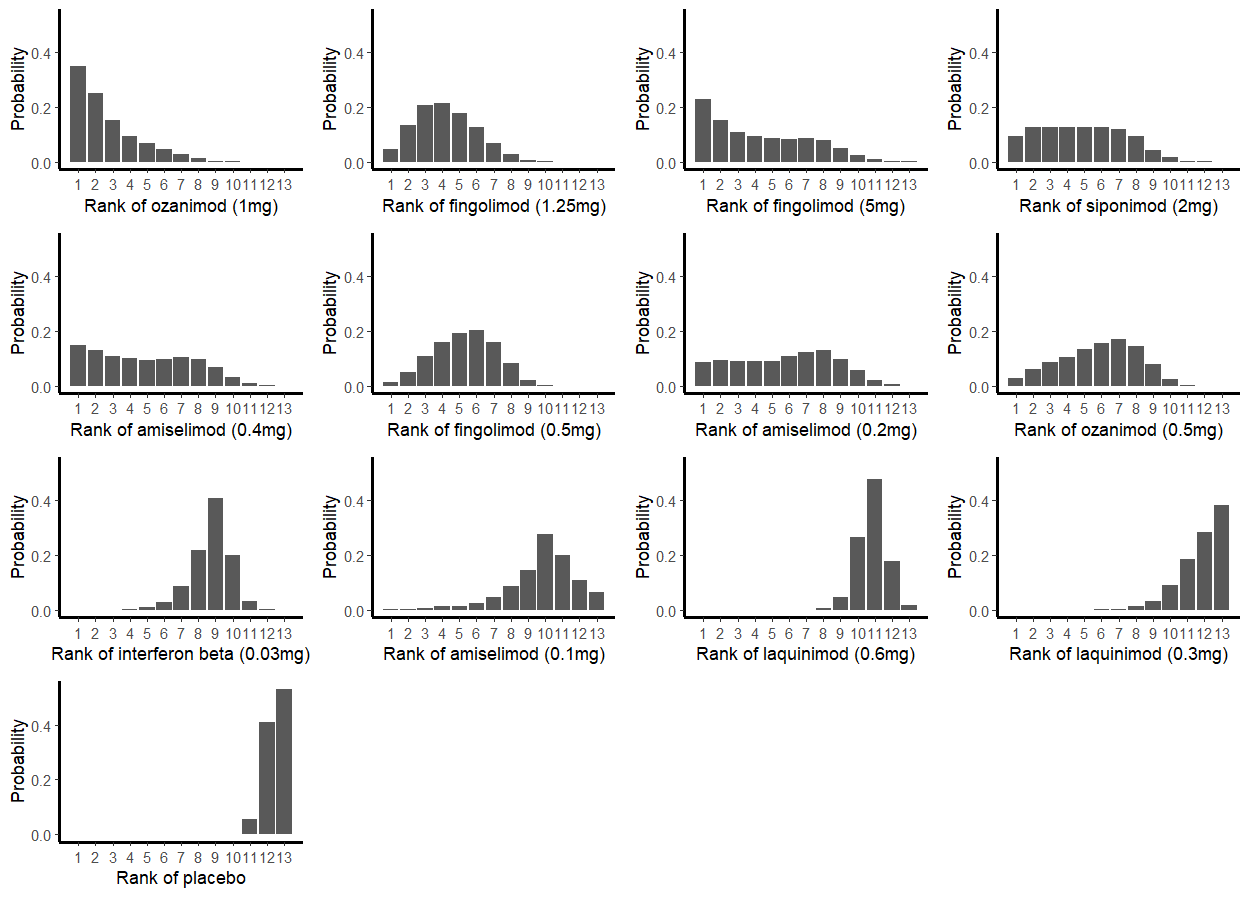


Supplementary Figure 5: Rank plot GD Lesion


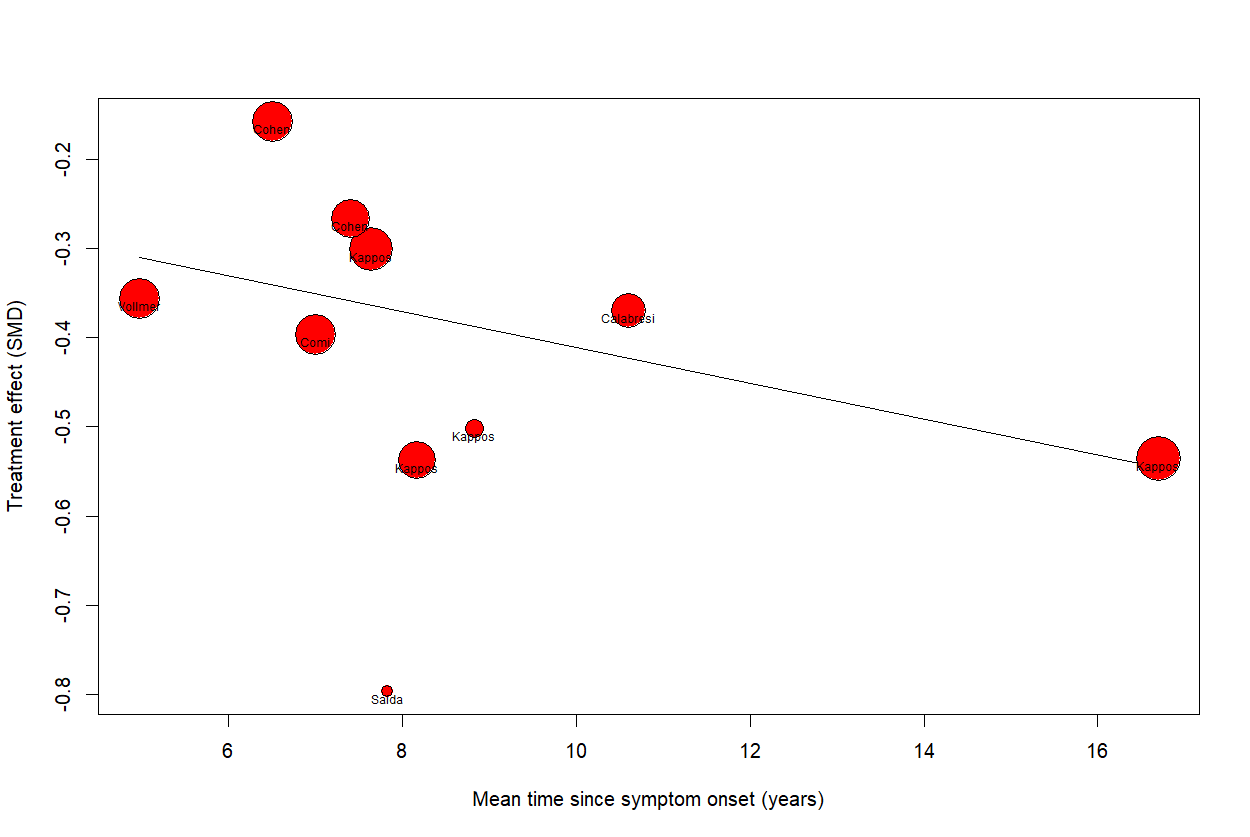


Supplementary Figure 6: Bubble plot GD lesions


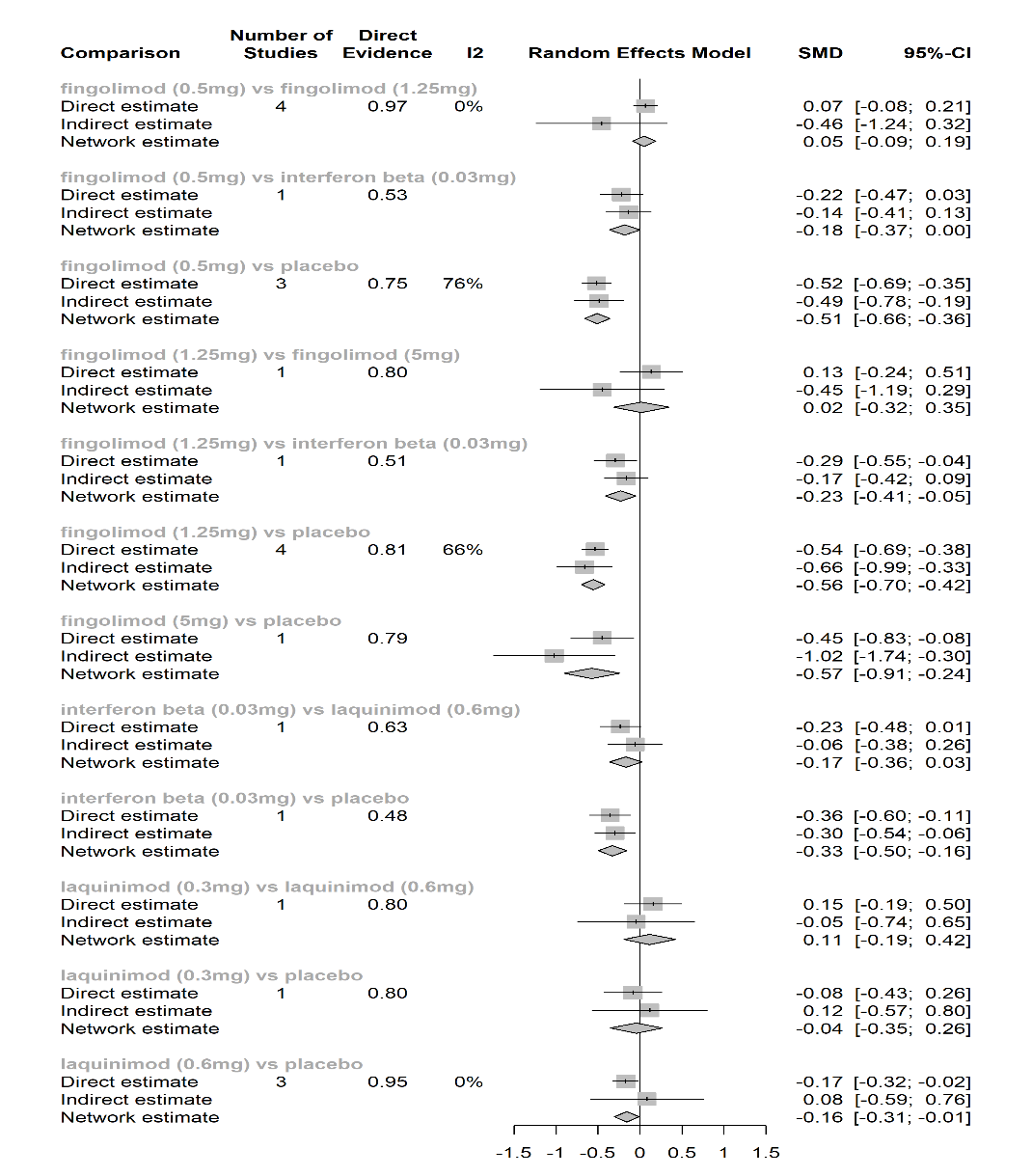


Supplementary Figure 7: Network split plot GD lesions

| amiselimod (0.1mg) | 0.02 [-0.28; 0.32] | 0.16 [-0.13; 0.46] | . | . | . | . | . | . | . | 0.12 [-0.17; 0.42] | . |
| --- | --- | --- | --- | --- | --- | --- | --- | --- | --- | --- | --- |
| 0.02 [-0.28; 0.32] | amiselimod (0.2mg) | 0.14 [-0.15; 0.44] | . | . | . | . | . | . | . | 0.10 [-0.19; 0.40] | . |
| 0.16 [-0.13; 0.46] | 0.14 [-0.15; 0.44] | amiselimod (0.4mg) | . | . | . | . | . | . | . | -0.04 [-0.34; 0.26] | . |
| 0.38 [ 0.06; 0.70] | 0.36 [ 0.03; 0.68] | 0.21 [-0.11; 0.53] | fingolimod (0.5mg) | 0.05 [-0.06; 0.16] | -0.21 [-0.38; -0.03] | . | . | . | . | -0.27 [-0.42; -0.13] | . |
| 0.43 [ 0.11; 0.75] | 0.41 [ 0.09; 0.73] | 0.26 [-0.06; 0.58] | 0.05 [-0.06; 0.16] | fingolimod (1.25mg) | -0.22 [-0.40; -0.04] | . | . | . | . | -0.35 [-0.49; -0.21] | . |
| 0.14 [-0.18; 0.46] | 0.12 [-0.21; 0.44] | -0.03 [-0.35; 0.30] | -0.24 [-0.37; -0.10] | -0.29 [-0.43; -0.15] | interferon beta (0.03mg) | 0.31 [ 0.13; 0.49] | . | 0.23 [ 0.10; 0.35] | 0.29 [ 0.16; 0.42] | 0.09 [-0.09; 0.26] | . |
| 0.45 [ 0.13; 0.77] | 0.43 [ 0.11; 0.75] | 0.29 [-0.03; 0.61] | 0.08 [-0.08; 0.23] | 0.02 [-0.13; 0.18] | 0.31 [ 0.17; 0.46] | laquinimod (0.6mg) | 0.00 [-0.16; 0.16] | . | . | -0.31 [-0.43; -0.19] | . |
| 0.48 [ 0.15; 0.82] | 0.46 [ 0.13; 0.80] | 0.32 [-0.02; 0.65] | 0.11 [-0.08; 0.30] | 0.05 [-0.14; 0.25] | 0.34 [ 0.16; 0.53] | 0.03 [-0.12; 0.18] | laquinimod (1.2mg) | . | . | -0.39 [-0.55; -0.23] | . |
| 0.36 [ 0.02; 0.71] | 0.34 [ 0.00; 0.69] | 0.20 [-0.15; 0.54] | -0.01 [-0.20; 0.17] | -0.06 [-0.25; 0.12] | 0.23 [ 0.10; 0.35] | -0.09 [-0.28; 0.10] | -0.12 [-0.34; 0.11] | ozanimod (0.5mg) | 0.06 [-0.06; 0.19] | . | . |
| 0.43 [ 0.08; 0.77] | 0.41 [ 0.06; 0.75] | 0.26 [-0.08; 0.61] | 0.05 [-0.13; 0.24] | -0.00 [-0.19; 0.19] | 0.29 [ 0.16; 0.42] | -0.02 [-0.22; 0.17] | -0.05 [-0.28; 0.17] | 0.06 [-0.06; 0.19] | ozanimod (1mg) | . | . |
| 0.12 [-0.17; 0.42] | 0.10 [-0.19; 0.40] | -0.04 [-0.34; 0.26] | -0.25 [-0.37; -0.13] | -0.30 [-0.43; -0.18] | -0.01 [-0.14; 0.11] | -0.33 [-0.44; -0.21] | -0.36 [-0.51; -0.21] | -0.24 [-0.42; -0.06] | -0.30 [-0.48; -0.12] | placebo | 0.25 [ 0.06; 0.45] |
| 0.38 [ 0.02; 0.73] | 0.36 [ 0.00; 0.71] | 0.21 [-0.14; 0.57] | 0.00 [-0.23; 0.23] | -0.05 [-0.28; 0.18] | 0.24 [ 0.01; 0.47] | -0.07 [-0.30; 0.15] | -0.10 [-0.35; 0.14] | 0.01 [-0.25; 0.28] | -0.05 [-0.31; 0.22] | 0.25 [ 0.06; 0.45] | siponimod (2mg) |

Supplementary Table 5: League Table Brain vol


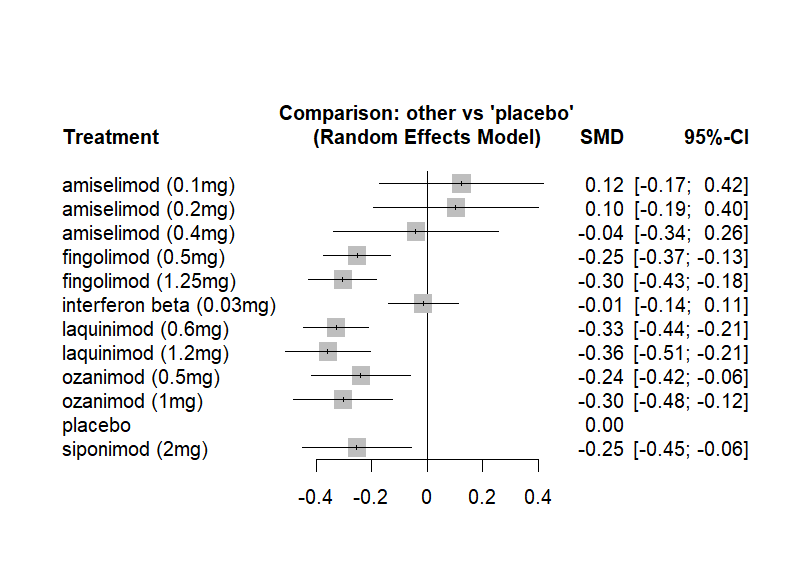


Supplementary Figure 8: Forest plot brain vol


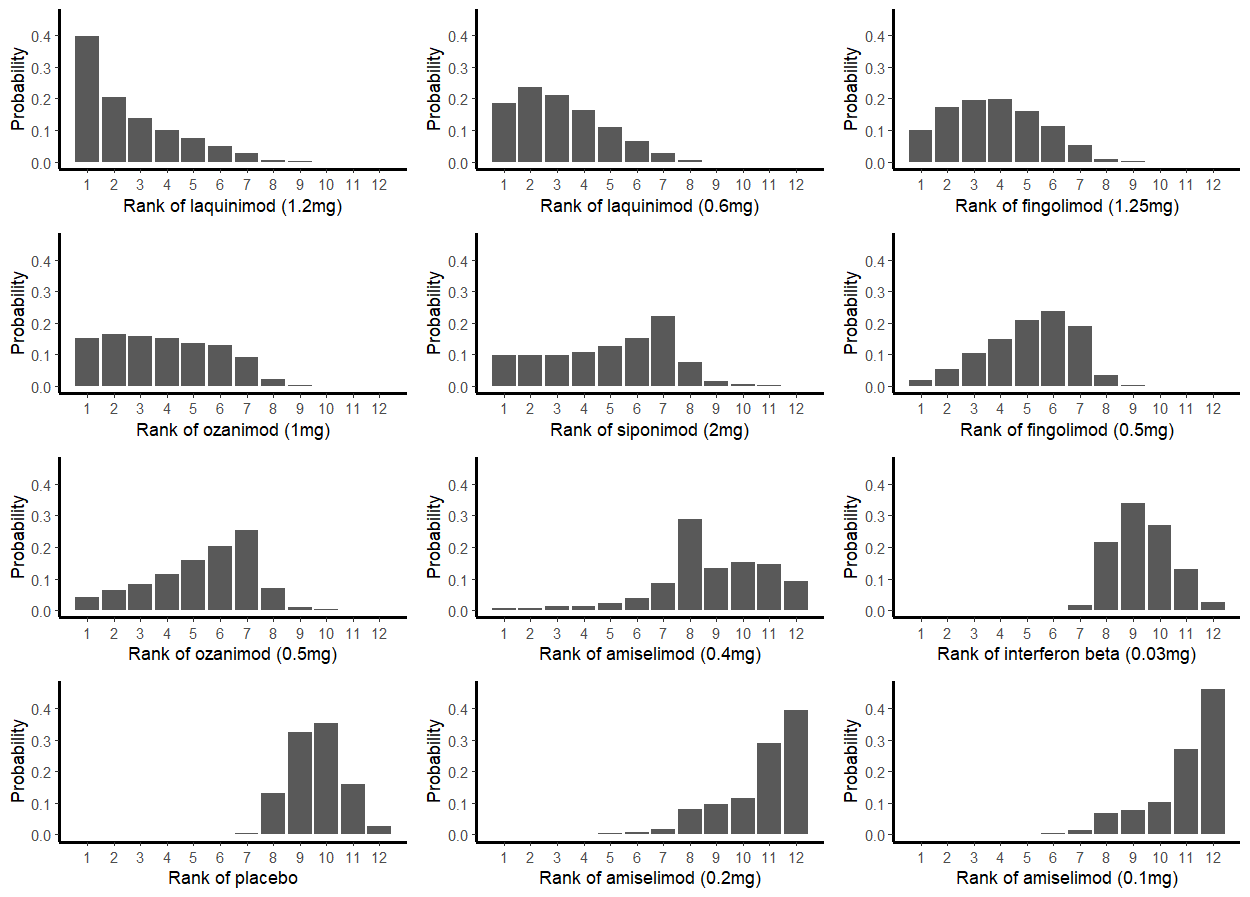


Supplementary Figure 9: Rank plot Brain vol


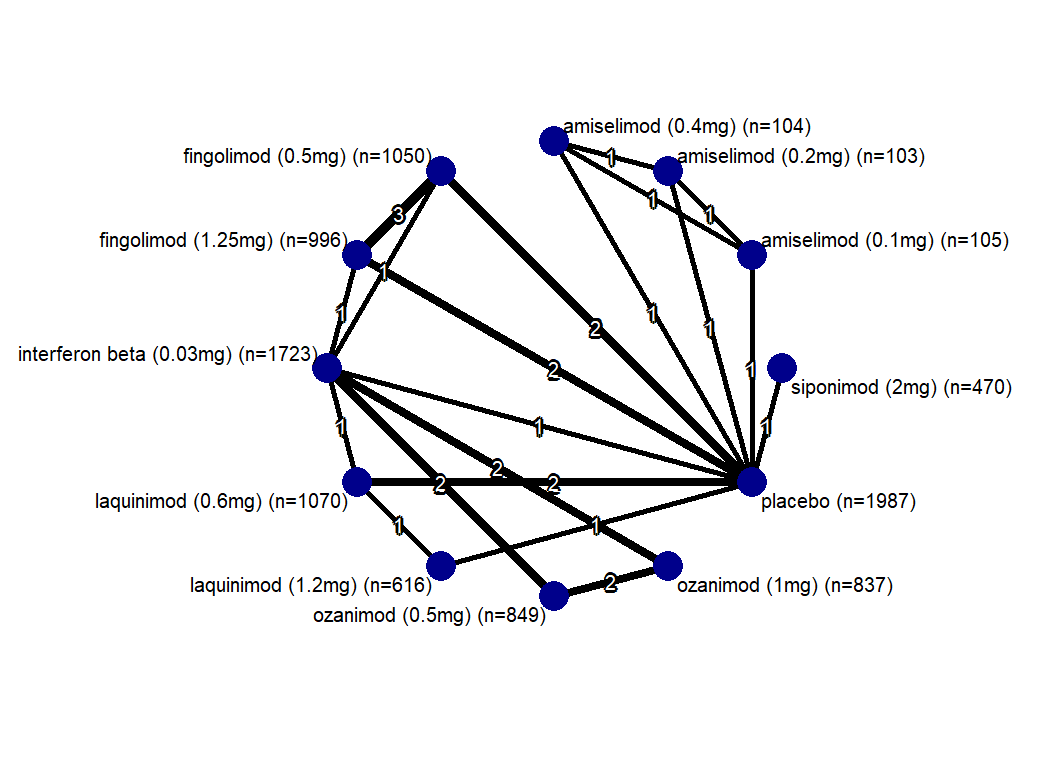


Supplementary Figure 10: Network plot brain vols


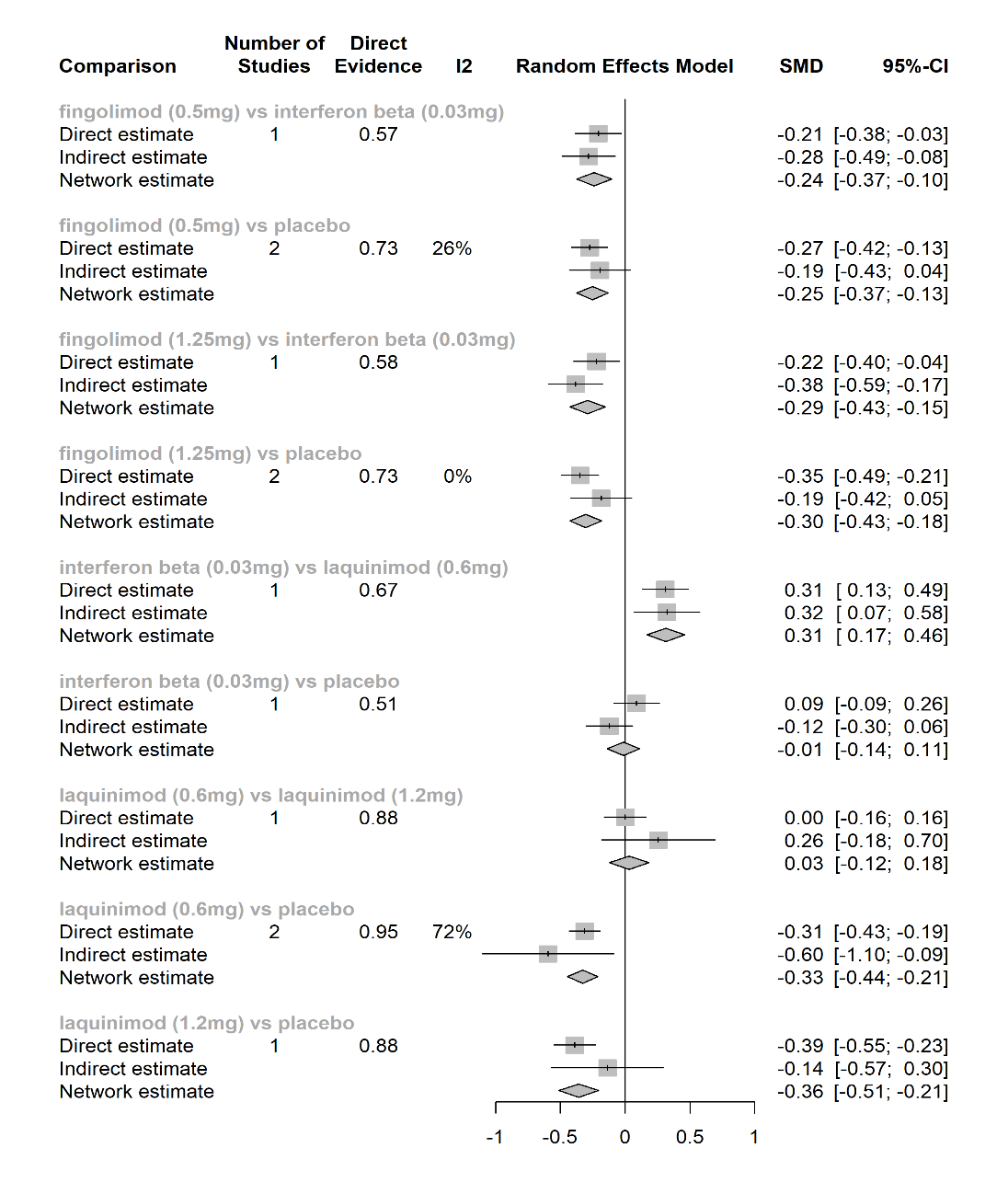


Supplementary Figure 11: Network Split Plot Brain Volume

| amiselimod (0.1mg) | 0.68 [0.28; 1.68] | 0.67 [0.27; 1.66] | . | . | . | . | . | . | . | 1.49 [0.65; 3.45] | . | . | . | . | . | . |
| --- | --- | --- | --- | --- | --- | --- | --- | --- | --- | --- | --- | --- | --- | --- | --- | --- |
| 0.68 [0.28; 1.68] | amiselimod (0.2mg) | 0.99 [0.39; 2.53] | . | . | . | . | . | . | . | 2.19 [0.91; 5.27] | . | . | . | . | . | . |
| 0.67 [0.27; 1.66] | 0.99 [0.39; 2.53] | amiselimod (0.4mg) | . | . | . | . | . | . | . | 2.22 [0.92; 5.33] | . | . | . | . | . | . |
| 0.58 [0.23; 1.43] | 0.85 [0.33; 2.18] | 0.86 [0.33; 2.21] | fingolimod (0.5mg) | 1.06 [0.76; 1.50] | . | 1.30 [0.69; 2.44] | . | . | . | 2.45 [1.65; 3.64] | . | . | . | . | . | . |
| 0.60 [0.24; 1.47] | 0.87 [0.34; 2.24] | 0.88 [0.34; 2.26] | 1.03 [0.74; 1.44] | fingolimod (1.25mg) | 0.86 [0.25; 2.99] | 0.70 [0.38; 1.31] | . | . | . | 3.11 [2.14; 4.52] | . | . | . | . | . | . |
| 0.33 [0.09; 1.24] | 0.48 [0.12; 1.86] | 0.48 [0.12; 1.89] | 0.56 [0.19; 1.66] | 0.55 [0.19; 1.58] | fingolimod (5mg) | . | . | . | . | 5.65 [1.91; 16.69] | . | . | . | . | . | . |
| 0.74 [0.29; 1.89] | 1.09 [0.41; 2.87] | 1.10 [0.42; 2.90] | 1.28 [0.82; 2.01] | 1.25 [0.80; 1.94] | 2.27 [0.75; 6.84] | interferon beta (0.03mg) | . | 1.16 [0.63; 2.12] | . | 1.40 [0.77; 2.54] | . | . | . | . | . | . |
| 1.72 [0.58; 5.11] | 2.53 [0.82; 7.74] | 2.56 [0.83; 7.82] | 2.98 [1.38; 6.44] | 2.89 [1.34; 6.23] | 5.27 [1.52; 18.34] | 2.32 [1.05; 5.11] | laquinimod (0.3mg) | 0.60 [0.27; 1.32] | . | 0.86 [0.40; 1.88] | . | . | . | . | . | . |
| 1.02 [0.42; 2.50] | 1.50 [0.59; 3.81] | 1.52 [0.60; 3.85] | 1.77 [1.13; 2.77] | 1.72 [1.11; 2.67] | 3.14 [1.07; 9.23] | 1.38 [0.88; 2.18] | 0.60 [0.30; 1.19] | laquinimod (0.6mg) | 0.74 [0.42; 1.32] | 1.39 [1.02; 1.89] | . | . | . | . | . | . |
| 0.78 [0.29; 2.08] | 1.14 [0.41; 3.16] | 1.15 [0.42; 3.20] | 1.34 [0.72; 2.50] | 1.30 [0.71; 2.41] | 2.38 [0.75; 7.58] | 1.05 [0.55; 1.99] | 0.45 [0.19; 1.05] | 0.76 [0.45; 1.28] | laquinimod (1.2mg) | 1.88 [1.06; 3.34] | . | . | . | . | . | . |
| 1.49 [0.65; 3.45] | 2.19 [0.91; 5.27] | 2.22 [0.92; 5.33] | 2.58 [1.82; 3.66] | 2.51 [1.79; 3.51] | 4.57 [1.62; 12.89] | 2.01 [1.33; 3.04] | 0.87 [0.43; 1.73] | 1.46 [1.07; 1.98] | 1.92 [1.14; 3.24] | placebo | 0.64 [0.27; 1.51] | 0.87 [0.38; 2.00] | 0.37 [0.15; 0.94] | 0.36 [0.13; 0.99] | 0.26 [0.09; 0.72] | 0.13 [0.04; 0.44] |
| 0.95 [0.28; 3.17] | 1.39 [0.40; 4.79] | 1.41 [0.41; 4.84] | 1.64 [0.64; 4.18] | 1.59 [0.63; 4.04] | 2.91 [0.75; 11.23] | 1.28 [0.49; 3.34] | 0.55 [0.18; 1.67] | 0.93 [0.37; 2.32] | 1.22 [0.44; 3.36] | 0.64 [0.27; 1.51] | ponesimod (10mg) | 1.38 [0.57; 3.33] | 0.59 [0.22; 1.55] | . | . | . |
| 1.30 [0.40; 4.24] | 1.91 [0.57; 6.41] | 1.94 [0.58; 6.48] | 2.26 [0.92; 5.55] | 2.19 [0.90; 5.36] | 3.99 [1.06; 15.07] | 1.76 [0.70; 4.44] | 0.76 [0.26; 2.23] | 1.27 [0.53; 3.08] | 1.68 [0.63; 4.47] | 0.87 [0.38; 2.00] | 1.37 [0.57; 3.33] | ponesimod (20mg) | 0.43 [0.17; 1.09] | . | . | . |
| 0.56 [0.16; 1.94] | 0.82 [0.23; 2.92] | 0.82 [0.23; 2.95] | 0.96 [0.36; 2.58] | 0.93 [0.35; 2.50] | 1.70 [0.42; 6.83] | 0.75 [0.27; 2.06] | 0.32 [0.10; 1.03] | 0.54 [0.20; 1.44] | 0.72 [0.25; 2.07] | 0.37 [0.15; 0.94] | 0.59 [0.22; 1.55] | 0.43 [0.17; 1.09] | ponesimod (40mg) | . | . | . |
| 0.53 [0.14; 1.99] | 0.78 [0.20; 3.00] | 0.79 [0.21; 3.04] | 0.92 [0.31; 2.71] | 0.90 [0.31; 2.62] | 1.63 [0.38; 6.98] | 0.72 [0.24; 2.16] | 0.31 [0.09; 1.06] | 0.52 [0.18; 1.51] | 0.69 [0.22; 2.16] | 0.36 [0.13; 0.99] | 0.56 [0.15; 2.14] | 0.41 [0.11; 1.52] | 0.96 [0.24; 3.80] | siponimod (0.5mg) | 0.72 [0.23; 2.27] | 0.38 [0.10; 1.35] |
| 0.39 [0.10; 1.46] | 0.57 [0.15; 2.19] | 0.57 [0.15; 2.22] | 0.67 [0.23; 1.98] | 0.65 [0.22; 1.91] | 1.18 [0.27; 5.09] | 0.52 [0.17; 1.58] | 0.22 [0.06; 0.78] | 0.38 [0.13; 1.10] | 0.50 [0.16; 1.58] | 0.26 [0.09; 0.72] | 0.41 [0.11; 1.56] | 0.30 [0.08; 1.11] | 0.70 [0.17; 2.77] | 0.72 [0.23; 2.27] | siponimod (10mg) | 0.52 [0.14; 1.88] |
| 0.20 [0.05; 0.85] | 0.29 [0.07; 1.28] | 0.30 [0.07; 1.29] | 0.35 [0.10; 1.18] | 0.34 [0.10; 1.15] | 0.61 [0.13; 2.95] | 0.27 [0.08; 0.94] | 0.12 [0.03; 0.46] | 0.20 [0.06; 0.66] | 0.26 [0.07; 0.94] | 0.13 [0.04; 0.44] | 0.21 [0.05; 0.91] | 0.15 [0.04; 0.65] | 0.36 [0.08; 1.61] | 0.37 [0.10; 1.35] | 0.52 [0.14; 1.88] | siponimod (2mg) |

Supplementary Table 6: League table for relapse free


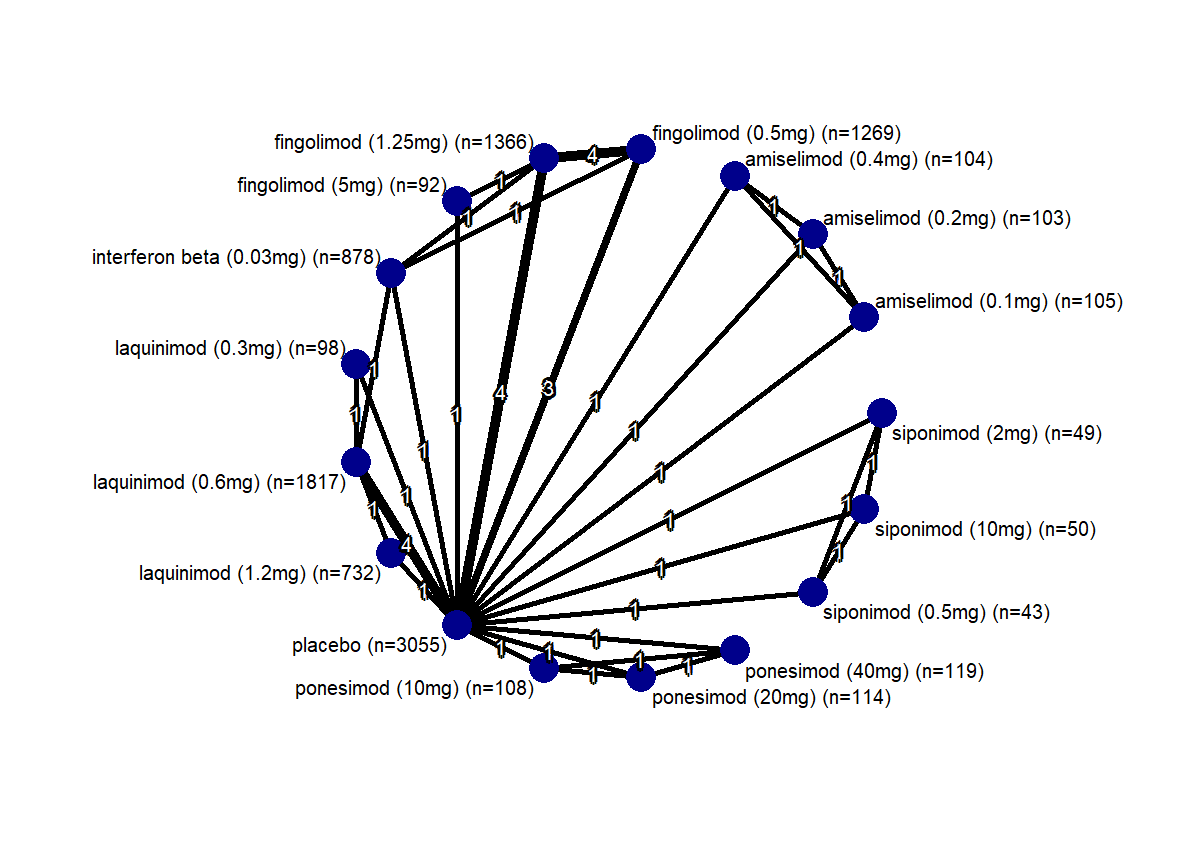


Supplementary Figure 12: Network relapse free


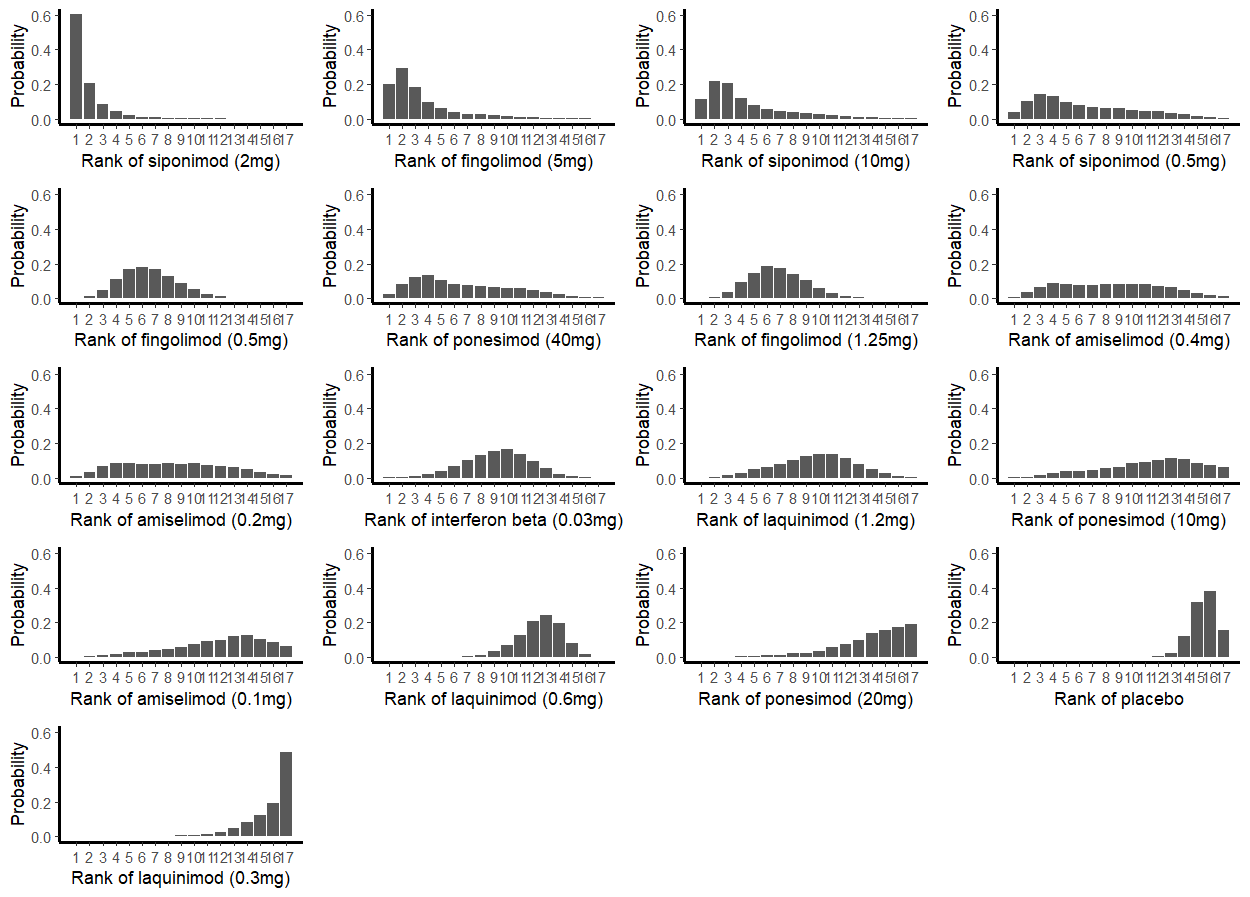


Supplementary Figure 13: Rank plot relapse free


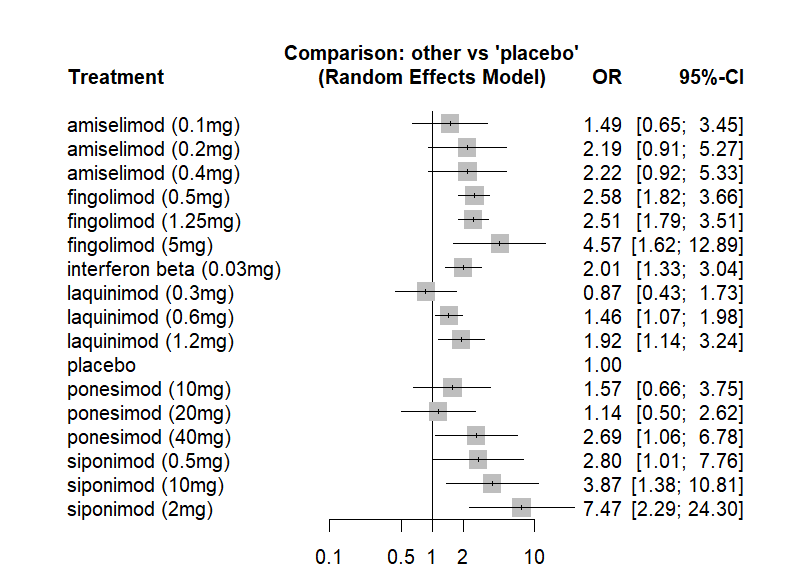


Supplementary Figure 14: Forest plot relapse free


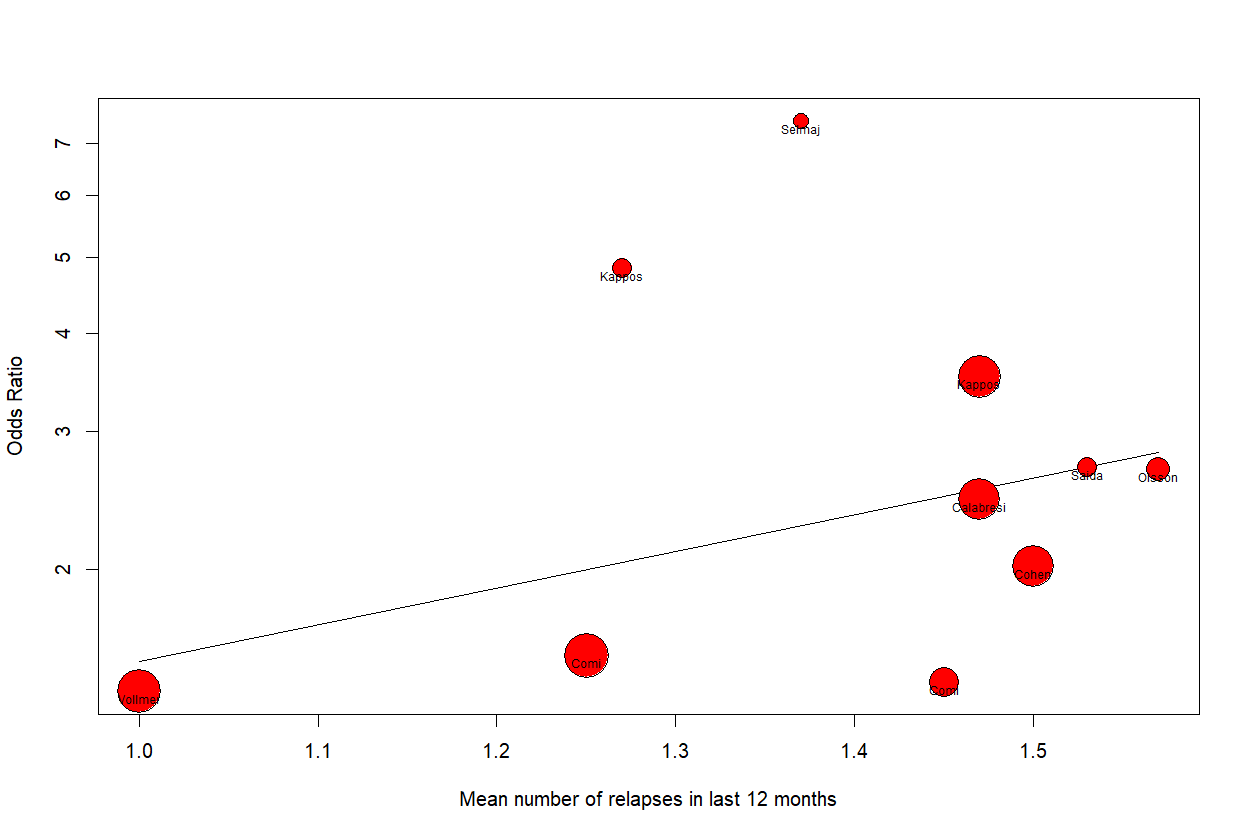


Supplementary Figure 15: Bubble plot relapse free


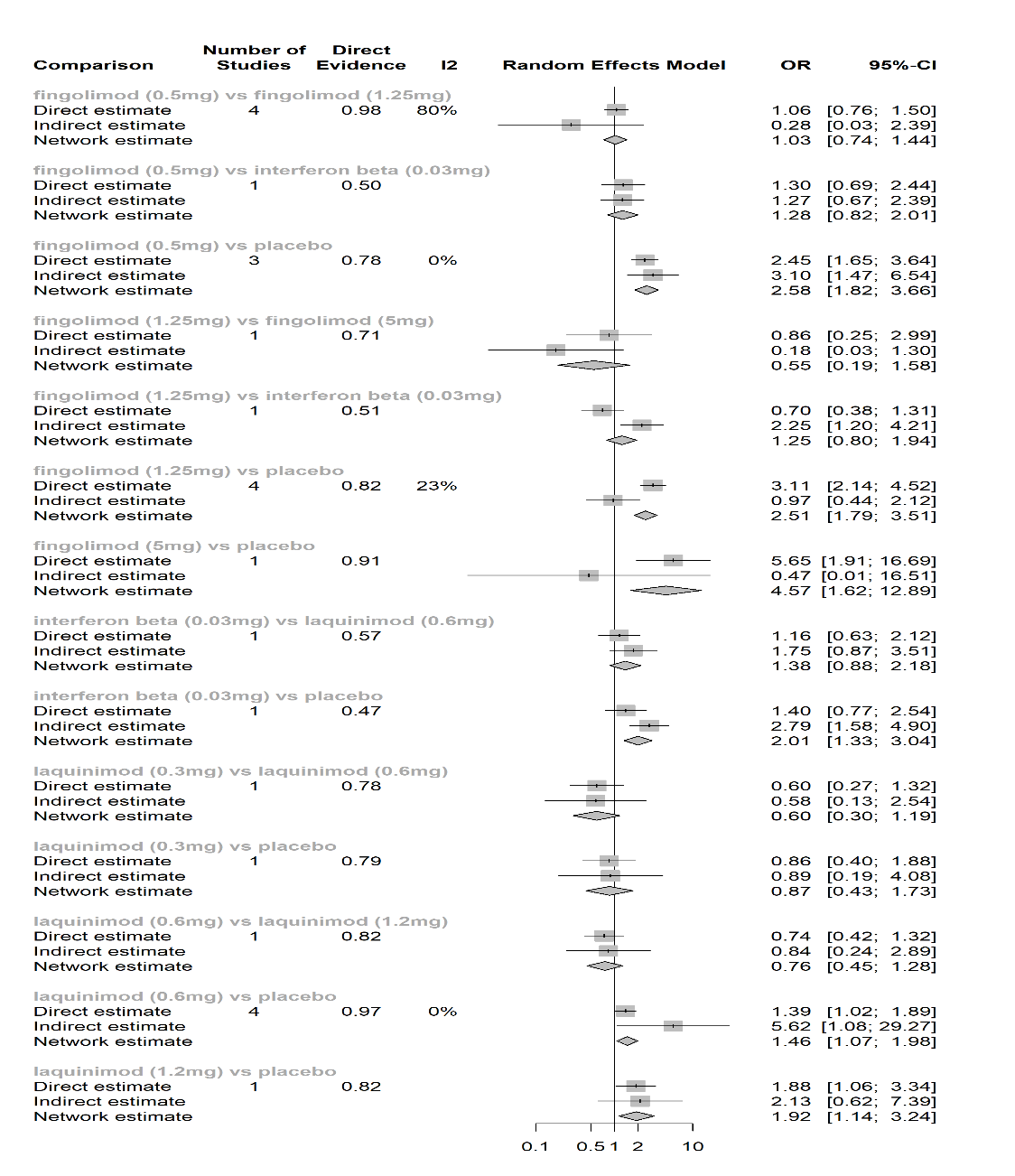


Supplementary Figure 16: Network split plot relapse free

| amiselimod (0.1mg) | 0.63 [0.36; 1.11] | 1.02 [0.59; 1.76] | . | . | . | . | . | . | . | . | . | . | 0.72 [0.41; 1.26] | . | . | . | . | . | . | . | . | amiselimod (0.1mg) |
| --- | --- | --- | --- | --- | --- | --- | --- | --- | --- | --- | --- | --- | --- | --- | --- | --- | --- | --- | --- | --- | --- | --- |
| 0.63 [0.36; 1.11] | amiselimod (0.2mg) | 1.61 [0.92; 2.83] | . | . | . | . | . | . | . | . | . | . | 1.14 [0.64; 2.02] | . | . | . | . | . | . | . | . | 0.63 [0.36; 1.11] |
| 1.02 [0.59; 1.76] | 1.61 [0.92; 2.83] | amiselimod (0.4mg) | . | . | . | . | . | . | . | . | . | . | 0.71 [0.40; 1.24] | . | . | . | . | . | . | . | . | 1.02 [0.59; 1.76] |
| 0.57 [0.30; 1.08] | 0.90 [0.46; 1.73] | 0.56 [0.29; 1.06] | fingolimod (0.5mg) | 0.81 [0.59; 1.10] | . | 0.56 [0.36; 0.87] | . | . | . | . | . | . | 1.54 [1.00; 2.38] | . | . | . | . | . | . | . | . | 0.57 [0.30; 1.08] |
| 0.47 [0.25; 0.89] | 0.75 [0.39; 1.43] | 0.46 [0.25; 0.88] | 0.83 [0.61; 1.13] | fingolimod (1.25mg) | 0.13 [0.03; 0.57] | 0.87 [0.54; 1.39] | . | . | . | . | . | . | 1.37 [0.94; 2.00] | . | . | . | . | . | . | . | . | 0.47 [0.25; 0.89] |
| 0.07 [0.01; 0.32] | 0.11 [0.02; 0.51] | 0.07 [0.01; 0.32] | 0.12 [0.03; 0.52] | 0.14 [0.03; 0.62] | fingolimod (5mg) | . | . | . | . | . | . | . | 9.47 [2.11; 42.52] | . | . | . | . | . | . | . | . | 0.07 [0.01; 0.32] |
| 0.36 [0.20; 0.67] | 0.57 [0.30; 1.07] | 0.35 [0.19; 0.66] | 0.64 [0.46; 0.89] | 0.76 [0.55; 1.07] | 5.35 [1.22; 23.41] | interferon beta (0.03mg) | . | 1.52 [1.10; 2.10] | . | . | 2.02 [1.63; 2.50] | 1.88 [1.51; 2.33] | 1.97 [1.44; 2.69] | . | . | . | . | . | . | . | . | 0.36 [0.20; 0.67] |
| 0.44 [0.19; 1.04] | 0.70 [0.29; 1.66] | 0.43 [0.18; 1.02] | 0.78 [0.38; 1.60] | 0.93 [0.46; 1.92] | 6.54 [1.32; 32.38] | 1.22 [0.61; 2.44] | laquinimod (0.3mg) | 1.62 [0.79; 3.31] | . | . | . | . | 1.19 [0.56; 2.51] | . | . | . | . | . | . | . | . | 0.44 [0.19; 1.04] |
| 0.55 [0.31; 0.99] | 0.88 [0.48; 1.59] | 0.54 [0.31; 0.97] | 0.98 [0.69; 1.38] | 1.17 [0.84; 1.64] | 8.21 [1.89; 35.66] | 1.53 [1.17; 2.00] | 1.26 [0.66; 2.40] | laquinimod (0.6mg) | 0.95 [0.74; 1.22] | 2.43 [1.32; 4.49] | . | . | 1.30 [1.11; 1.51] | . | . | . | . | . | . | . | . | 0.55 [0.31; 0.99] |
| 0.54 [0.30; 0.99] | 0.86 [0.46; 1.59] | 0.53 [0.29; 0.97] | 0.95 [0.65; 1.40] | 1.15 [0.78; 1.67] | 8.01 [1.83; 35.16] | 1.50 [1.08; 2.08] | 1.23 [0.62; 2.42] | 0.98 [0.78; 1.23] | laquinimod (1.2mg) | . | . | . | 1.30 [1.02; 1.66] | . | . | . | . | . | . | . | . | 0.54 [0.30; 0.99] |
| 1.31 [0.61; 2.82] | 2.08 [0.95; 4.52] | 1.29 [0.60; 2.77] | 2.31 [1.26; 4.26] | 2.78 [1.52; 5.09] | 19.45 [4.12; 91.87] | 3.64 [2.05; 6.46] | 2.98 [1.30; 6.81] | 2.37 [1.40; 4.01] | 2.43 [1.39; 4.25] | laquinimod (1.5mg) | . | . | 0.56 [0.31; 1.00] | . | . | . | . | . | . | . | . | 1.31 [0.61; 2.82] |
| 0.73 [0.38; 1.39] | 1.15 [0.59; 2.24] | 0.72 [0.37; 1.37] | 1.28 [0.86; 1.91] | 1.54 [1.04; 2.30] | 10.79 [2.43; 47.97] | 2.02 [1.63; 2.50] | 1.65 [0.80; 3.41] | 1.31 [0.93; 1.85] | 1.35 [0.91; 1.99] | 0.55 [0.30; 1.02] | ozanimod (0.5mg) | 0.93 [0.76; 1.14] | . | . | . | . | . | . | . | . | . | 0.73 [0.38; 1.39] |
| 0.68 [0.35; 1.30] | 1.07 [0.55; 2.09] | 0.67 [0.35; 1.28] | 1.20 [0.80; 1.78] | 1.44 [0.96; 2.14] | 10.05 [2.26; 44.68] | 1.88 [1.51; 2.33] | 1.54 [0.74; 3.18] | 1.22 [0.87; 1.73] | 1.25 [0.85; 1.86] | 0.52 [0.28; 0.95] | 0.93 [0.76; 1.14] | ozanimod (1mg) | . | . | . | . | . | . | . | . | . | 0.68 [0.35; 1.30] |
| 0.72 [0.41; 1.26] | 1.14 [0.64; 2.02] | 0.71 [0.40; 1.24] | 1.27 [0.92; 1.75] | 1.52 [1.12; 2.08] | 10.66 [2.47; 45.99] | 1.99 [1.54; 2.57] | 1.63 [0.85; 3.13] | 1.30 [1.12; 1.51] | 1.33 [1.06; 1.67] | 0.55 [0.32; 0.92] | 0.99 [0.71; 1.38] | 1.06 [0.76; 1.48] | placebo | 0.87 [0.48; 1.60] | 0.86 [0.47; 1.56] | 1.02 [0.57; 1.82] | 1.26 [0.51; 3.10] | 0.66 [0.23; 1.93] | 1.83 [0.74; 4.54] | 0.17 [0.04; 0.80] | 0.54 [0.41; 0.72] | 0.72 [0.41; 1.26] |
| 0.63 [0.28; 1.43] | 0.99 [0.43; 2.29] | 0.62 [0.27; 1.41] | 1.11 [0.56; 2.20] | 1.33 [0.68; 2.63] | 9.32 [1.92; 45.37] | 1.74 [0.90; 3.36] | 1.43 [0.59; 3.47] | 1.14 [0.61; 2.12] | 1.16 [0.61; 2.22] | 0.48 [0.22; 1.07] | 0.86 [0.43; 1.72] | 0.93 [0.46; 1.85] | 0.87 [0.48; 1.60] | ponesimod (10mg) | 0.98 [0.52; 1.83] | 1.17 [0.64; 2.14] | . | . | . | . | . | 0.63 [0.28; 1.43] |
| 0.62 [0.27; 1.40] | 0.98 [0.43; 2.24] | 0.61 [0.27; 1.37] | 1.09 [0.55; 2.14] | 1.31 [0.67; 2.56] | 9.14 [1.88; 44.38] | 1.71 [0.89; 3.28] | 1.40 [0.58; 3.39] | 1.11 [0.60; 2.06] | 1.14 [0.60; 2.16] | 0.47 [0.21; 1.04] | 0.85 [0.43; 1.68] | 0.91 [0.46; 1.81] | 0.86 [0.47; 1.56] | 0.98 [0.52; 1.83] | ponesimod (20mg) | 1.19 [0.65; 2.17] | . | . | . | . | . | 0.62 [0.27; 1.40] |
| 0.74 [0.33; 1.64] | 1.16 [0.51; 2.63] | 0.72 [0.32; 1.61] | 1.30 [0.67; 2.51] | 1.56 [0.81; 3.00] | 10.90 [2.26; 52.52] | 2.04 [1.08; 3.83] | 1.67 [0.70; 3.98] | 1.33 [0.73; 2.41] | 1.36 [0.73; 2.53] | 0.56 [0.26; 1.22] | 1.01 [0.52; 1.97] | 1.08 [0.56; 2.12] | 1.02 [0.57; 1.82] | 1.17 [0.64; 2.14] | 1.19 [0.65; 2.17] | ponesimod (40mg) | . | . | . | . | . | 0.74 [0.33; 1.64] |
| 1.07 [0.38; 3.05] | 1.70 [0.59; 4.87] | 1.05 [0.37; 3.00] | 1.89 [0.74; 4.84] | 2.27 [0.89; 5.79] | 15.88 [2.88; 87.69] | 2.97 [1.18; 7.45] | 2.43 [0.81; 7.29] | 1.94 [0.79; 4.74] | 1.98 [0.80; 4.93] | 0.82 [0.29; 2.28] | 1.47 [0.57; 3.79] | 1.58 [0.61; 4.07] | 1.49 [0.62; 3.61] | 1.70 [0.58; 4.97] | 1.74 [0.60; 5.05] | 1.46 [0.51; 4.19] | siponimod (0.25mg) | 0.53 [0.18; 1.55] | 1.46 [0.58; 3.66] | 0.14 [0.03; 0.64] | 0.07 [0.01; 0.54] | 1.07 [0.38; 3.05] |
| 0.56 [0.17; 1.86] | 0.89 [0.27; 2.96] | 0.56 [0.17; 1.83] | 1.00 [0.33; 2.99] | 1.20 [0.40; 3.58] | 8.37 [1.38; 50.71] | 1.57 [0.53; 4.62] | 1.28 [0.37; 4.41] | 1.02 [0.35; 2.95] | 1.04 [0.36; 3.06] | 0.43 [0.13; 1.39] | 0.78 [0.26; 2.34] | 0.83 [0.28; 2.51] | 0.79 [0.27; 2.25] | 0.90 [0.27; 3.02] | 0.92 [0.27; 3.07] | 0.77 [0.23; 2.55] | 0.53 [0.18; 1.55] | Siponimod (0.5mg) | 2.76 [0.94; 8.16] | 0.26 [0.05; 1.35] | 0.13 [0.01; 1.11] | 0.56 [0.17; 1.86] |
| 1.56 [0.55; 4.46] | 2.47 [0.86; 7.12] | 1.53 [0.54; 4.38] | 2.75 [1.07; 7.08] | 3.31 [1.29; 8.48] | 23.14 [4.18; 128.10] | 4.33 [1.72; 10.91] | 3.54 [1.18; 10.66] | 2.82 [1.14; 6.95] | 2.89 [1.15; 7.22] | 1.19 [0.42; 3.34] | 2.15 [0.83; 5.54] | 2.30 [0.89; 5.95] | 2.17 [0.89; 5.28] | 2.48 [0.85; 7.28] | 2.53 [0.87; 7.39] | 2.12 [0.74; 6.13] | 1.46 [0.58; 3.66] | 2.76 [0.94; 8.16] | siponimod (1.25mg) | 0.09 [0.02; 0.44] | 0.05 [0.01; 0.37] | 1.56 [0.55; 4.46] |
| 0.15 [0.03; 0.74] | 0.23 [0.04; 1.19] | 0.14 [0.03; 0.73] | 0.26 [0.05; 1.23] | 0.31 [0.06; 1.48] | 2.15 [0.26; 17.95] | 0.40 [0.08; 1.91] | 0.33 [0.06; 1.75] | 0.26 [0.06; 1.23] | 0.27 [0.06; 1.27] | 0.11 [0.02; 0.56] | 0.20 [0.04; 0.96] | 0.21 [0.04; 1.03] | 0.20 [0.04; 0.94] | 0.23 [0.04; 1.20] | 0.24 [0.05; 1.22] | 0.20 [0.04; 1.02] | 0.14 [0.03; 0.64] | 0.26 [0.05; 1.35] | 0.09 [0.02; 0.44] | siponimod (10mg) | 0.50 [0.04; 5.70] | 0.15 [0.03; 0.74] |
| 0.39 [0.21; 0.73] | 0.62 [0.32; 1.17] | 0.38 [0.20; 0.72] | 0.69 [0.45; 1.05] | 0.82 [0.54; 1.25] | 5.77 [1.30; 25.57] | 1.08 [0.74; 1.58] | 0.88 [0.43; 1.80] | 0.70 [0.51; 0.97] | 0.72 [0.50; 1.03] | 0.30 [0.16; 0.54] | 0.53 [0.35; 0.83] | 0.57 [0.37; 0.89] | 0.54 [0.41; 0.72] | 0.62 [0.32; 1.21] | 0.63 [0.33; 1.22] | 0.53 [0.28; 1.01] | 0.36 [0.14; 0.91] | 0.69 [0.23; 2.03] | 0.25 [0.10; 0.63] | 2.68 [0.56; 12.74] | siponimod (2mg) | . |
| 0.64 [0.26; 1.56] | 1.01 [0.41; 2.50] | 0.63 [0.26; 1.54] | 1.13 [0.52; 2.44] | 1.36 [0.63; 2.91] | 9.48 [1.87; 47.96] | 1.77 [0.84; 3.73] | 1.45 [0.56; 3.77] | 1.16 [0.56; 2.36] | 1.18 [0.57; 2.47] | 0.49 [0.20; 1.17] | 0.88 [0.40; 1.91] | 0.94 [0.43; 2.05] | 0.89 [0.44; 1.79] | 1.02 [0.49; 2.10] | 1.04 [0.72; 1.49] | 0.87 [0.43; 1.75] | 0.60 [0.19; 1.84] | 1.13 [0.32; 4.01] | 0.41 [0.13; 1.27] | 4.41 [0.81; 23.87] | 1.64 [0.77; 3.50] | teriflunomide (14mg) |

Supplementary Table 7: League table AEs


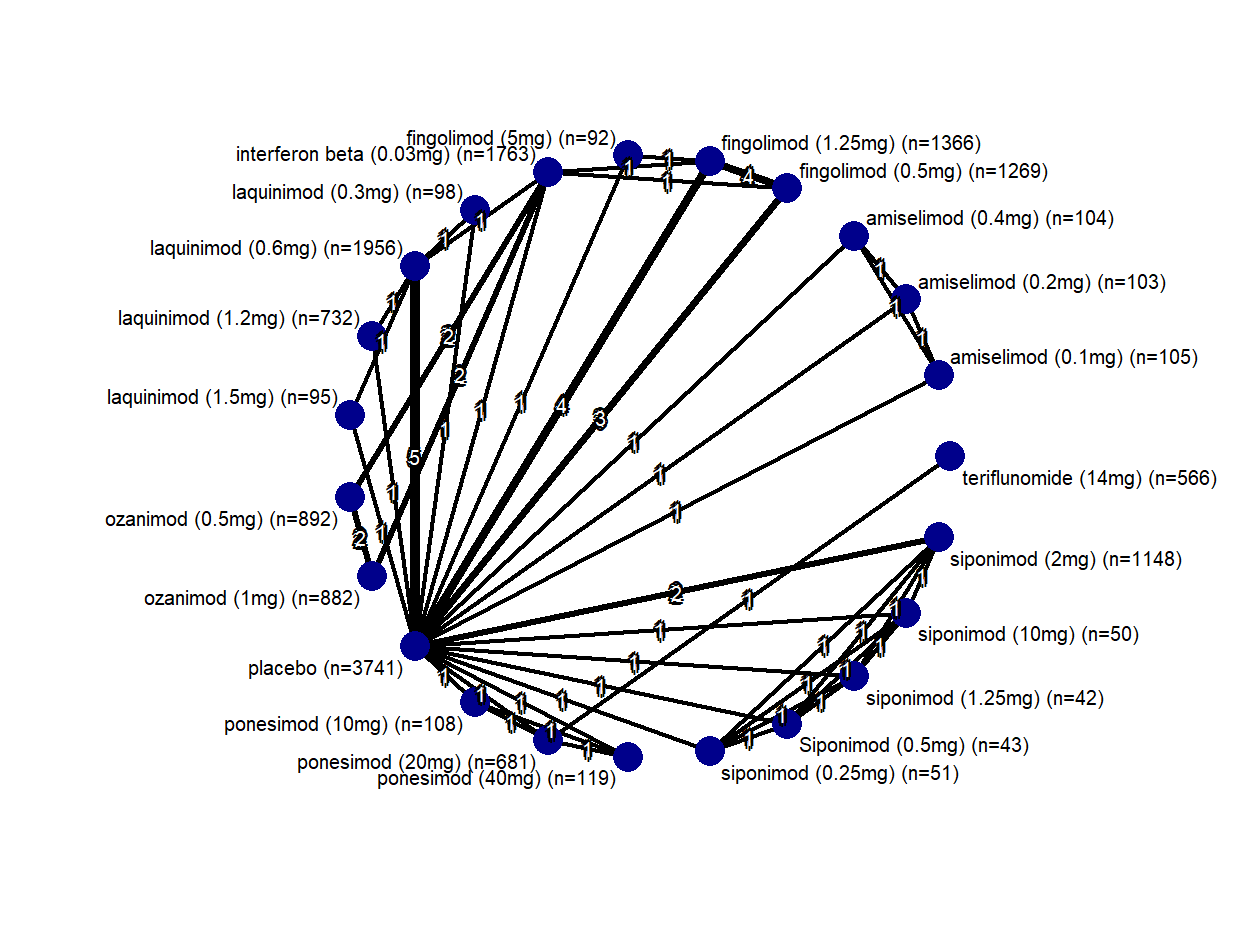


Supplementary Figure 17: Network plot for AEs


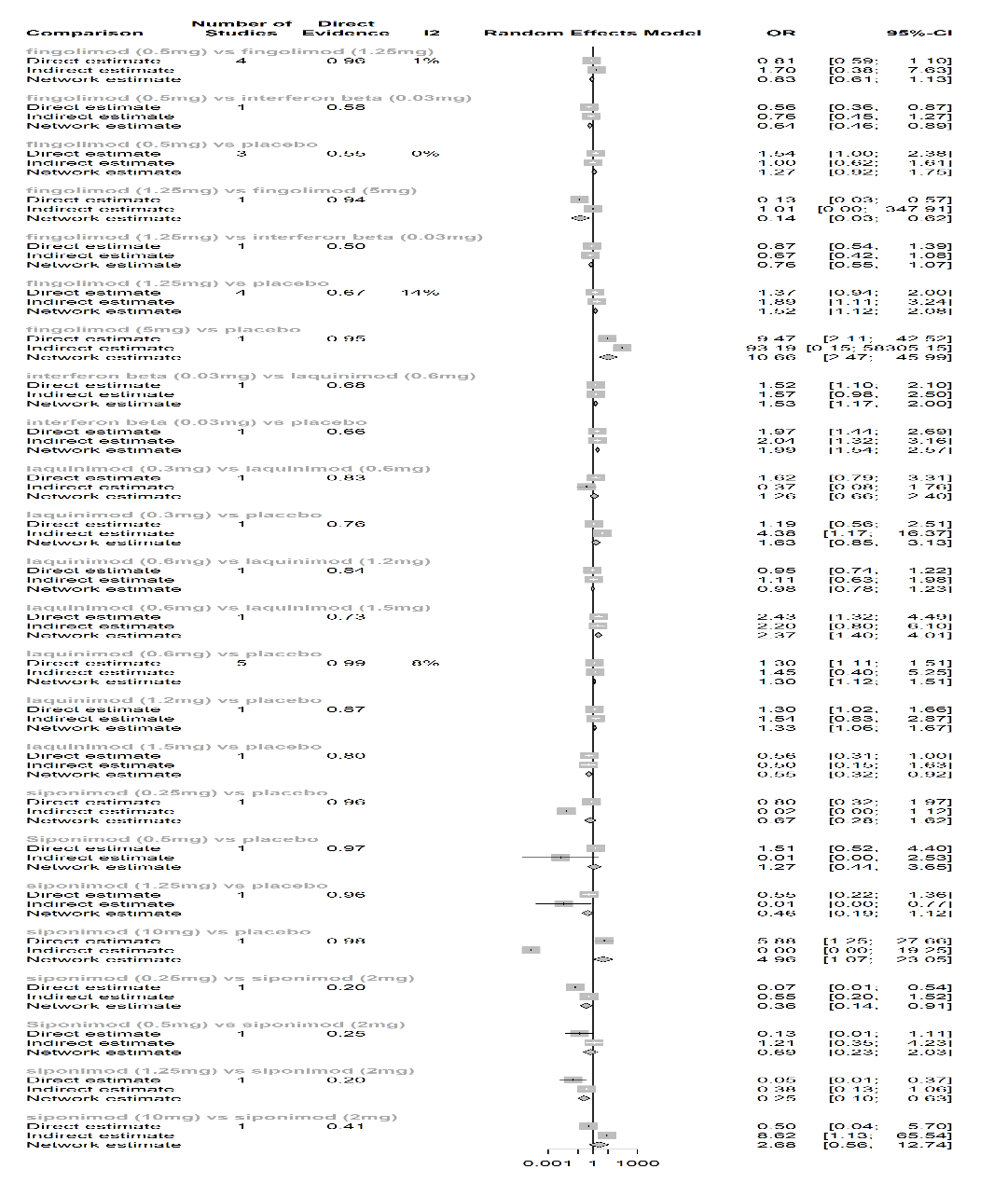


Supplementary Figure 18: Network split plot AEs

| amiselimod (0.1mg) | 1.13 [0.39; 3.31] | 1.35 [0.44; 4.10] | . | . | . | . | . | . | . | . | . | . | 0.77 [0.28; 2.07] | . | . | . | . | . | . | . | . | . |
| --- | --- | --- | --- | --- | --- | --- | --- | --- | --- | --- | --- | --- | --- | --- | --- | --- | --- | --- | --- | --- | --- | --- |
| 1.13 [0.39; 3.31] | amiselimod (0.2mg) | 1.19 [0.38; 3.74] | . | . | . | . | . | . | . | . | . | . | 0.68 [0.24; 1.89] | . | . | . | . | . | . | . | . | . |
| 1.35 [0.44; 4.10] | 1.19 [0.38; 3.74] | amiselimod (0.4mg) | . | . | . | . | . | . | . | . | . | . | 0.57 [0.20; 1.66] | . | . | . | . | . | . | . | . | . |
| 0.83 [0.29; 2.34] | 0.73 [0.25; 2.14] | 0.61 [0.20; 1.87] | fingolimod (0.5mg) | 0.80 [0.61; 1.05] | . | 1.22 [0.68; 2.19] | . | . | . | . | . | . | 0.98 [0.71; 1.36] | . | . | . | . | . | . | . | . | . |
| 0.65 [0.23; 1.84] | 0.58 [0.20; 1.68] | 0.49 [0.16; 1.47] | 0.79 [0.60; 1.04] | fingolimod (1.25mg) | 0.52 [0.20; 1.35] | 1.95 [1.13; 3.37] | . | . | . | . | . | . | 1.13 [0.83; 1.53] | . | . | . | . | . | . | . | . | . |
| 0.32 [0.09; 1.17] | 0.29 [0.08; 1.07] | 0.24 [0.06; 0.93] | 0.39 [0.17; 0.91] | 0.49 [0.22; 1.12] | fingolimod (5mg) | . | . | . | . | . | . | . | 2.57 [0.92; 7.17] | . | . | . | . | . | . | . | . | . |
| 1.15 [0.37; 3.63] | 1.02 [0.31; 3.30] | 0.86 [0.25; 2.88] | 1.40 [0.83; 2.36] | 1.76 [1.06; 2.95] | 3.58 [1.37; 9.34] | interferon beta (0.03mg) | . | . | . | . | 0.82 [0.52; 1.31] | 0.94 [0.58; 1.51] | . | . | . | . | . | . | . | . | . | . |
| 0.54 [0.12; 2.50] | 0.48 [0.10; 2.26] | 0.40 [0.08; 1.95] | 0.66 [0.20; 2.19] | 0.83 [0.25; 2.75] | 1.68 [0.40; 6.98] | 0.47 [0.13; 1.71] | laquinimod (0.3mg) | 1.85 [0.42; 8.05] | . | . | . | . | 1.04 [0.29; 3.78] | . | . | . | . | . | . | . | . | . |
| 0.61 [0.22; 1.71] | 0.54 [0.18; 1.57] | 0.45 [0.15; 1.37] | 0.74 [0.48; 1.13] | 0.93 [0.61; 1.42] | 1.88 [0.78; 4.53] | 0.53 [0.28; 1.00] | 1.12 [0.35; 3.63] | laquinimod (0.6mg) | 1.19 [0.76; 1.85] | 2.38 [0.63; 9.02] | . | . | 1.26 [0.94; 1.70] | . | . | . | . | . | . | . | . | . |
| 0.69 [0.23; 2.04] | 0.61 [0.20; 1.86] | 0.51 [0.16; 1.63] | 0.84 [0.49; 1.42] | 1.06 [0.63; 1.79] | 2.15 [0.85; 5.44] | 0.60 [0.29; 1.23] | 1.28 [0.38; 4.36] | 1.14 [0.75; 1.73] | laquinimod (1.2mg) | . | . | . | 1.16 [0.73; 1.86] | . | . | . | . | . | . | . | . | . |
| 1.28 [0.25; 6.47] | 1.13 [0.22; 5.84] | 0.95 [0.18; 5.04] | 1.55 [0.41; 5.79] | 1.96 [0.52; 7.29] | 3.96 [0.86; 18.20] | 1.11 [0.27; 4.51] | 2.36 [0.42; 13.20] | 2.10 [0.59; 7.52] | 1.85 [0.49; 6.97] | laquinimod (1.5mg) | . | . | 0.73 [0.18; 3.03] | . | . | . | . | . | . | . | . | . |
| 0.95 [0.28; 3.26] | 0.84 [0.24; 2.96] | 0.70 [0.19; 2.58] | 1.15 [0.57; 2.31] | 1.45 [0.73; 2.90] | 2.94 [1.01; 8.53] | 0.82 [0.52; 1.31] | 1.75 [0.44; 6.94] | 1.56 [0.71; 3.45] | 1.37 [0.58; 3.20] | 0.74 [0.17; 3.25] | ozanimod (0.5mg) | 1.14 [0.72; 1.81] | . | . | . | . | . | . | . | . | . | . |
| 1.09 [0.31; 3.75] | 0.96 [0.27; 3.41] | 0.81 [0.22; 2.97] | 1.31 [0.65; 2.67] | 1.66 [0.82; 3.35] | 3.37 [1.15; 9.83] | 0.94 [0.58; 1.52] | 2.01 [0.50; 7.99] | 1.79 [0.80; 3.98] | 1.57 [0.66; 3.70] | 0.85 [0.19; 3.74] | 1.15 [0.73; 1.81] | ozanimod (1mg) | . | . | . | . | . | . | . | . | . | . |
| 0.77 [0.28; 2.07] | 0.68 [0.24; 1.89] | 0.57 [0.20; 1.66] | 0.93 [0.68; 1.27] | 1.17 [0.87; 1.58] | 2.38 [1.04; 5.43] | 0.66 [0.38; 1.18] | 1.42 [0.44; 4.54] | 1.26 [0.94; 1.70] | 1.11 [0.72; 1.70] | 0.60 [0.17; 2.16] | 0.81 [0.39; 1.69] | 0.71 [0.34; 1.49] | placebo | 0.62 [0.19; 2.06] | 0.66 [0.20; 2.18] | 1.67 [0.38; 7.24] | 0.84 [0.02; 43.17] | 0.03 [0.00; 0.61] | 0.13 [0.01; 2.83] | 0.11 [0.01; 2.20] | 0.80 [0.56; 1.12] | . |
| 0.48 [0.10; 2.26] | 0.42 [0.09; 2.04] | 0.35 [0.07; 1.76] | 0.58 [0.17; 1.99] | 0.73 [0.21; 2.50] | 1.48 [0.35; 6.32] | 0.41 [0.11; 1.56] | 0.88 [0.17; 4.68] | 0.78 [0.23; 2.69] | 0.69 [0.19; 2.45] | 0.37 [0.06; 2.15] | 0.50 [0.12; 2.05] | 0.44 [0.11; 1.80] | 0.62 [0.19; 2.06] | ponesimod (10mg) | 1.06 [0.35; 3.19] | 2.68 [0.67; 10.80] | . | . | . | . | . | . |
| 0.51 [0.11; 2.39] | 0.45 [0.09; 2.16] | 0.38 [0.08; 1.87] | 0.61 [0.18; 2.10] | 0.77 [0.23; 2.65] | 1.57 [0.37; 6.69] | 0.44 [0.12; 1.65] | 0.93 [0.18; 4.96] | 0.83 [0.24; 2.85] | 0.73 [0.21; 2.60] | 0.40 [0.07; 2.28] | 0.53 [0.13; 2.17] | 0.47 [0.11; 1.90] | 0.66 [0.20; 2.18] | 1.06 [0.35; 3.19] | ponesimod (20mg) | 2.53 [0.63; 10.18] | . | . | . | . | . | 1.07 [0.67; 1.71] |
| 1.28 [0.22; 7.53] | 1.13 [0.19; 6.79] | 0.95 [0.15; 5.84] | 1.55 [0.35; 6.95] | 1.96 [0.44; 8.75] | 3.96 [0.74; 21.36] | 1.11 [0.23; 5.36] | 2.36 [0.36; 15.40] | 2.10 [0.47; 9.41] | 1.85 [0.40; 8.52] | 1.00 [0.14; 7.02] | 1.35 [0.26; 6.97] | 1.18 [0.23; 6.11] | 1.67 [0.38; 7.24] | 2.68 [0.67; 10.80] | 2.53 [0.63; 10.18] | ponesimod (40mg) | . | . | . | . | . | . |
| 4.84 [0.21; 109.31] | 4.28 [0.19; 97.72] | 3.60 [0.16; 83.24] | 5.87 [0.30; 114.37] | 7.41 [0.38; 144.28] | 15.02 [0.70; 322.54] | 4.20 [0.21; 85.05] | 8.96 [0.37; 214.30] | 7.97 [0.41; 155.19] | 6.99 [0.35; 138.36] | 3.79 [0.15; 94.83] | 5.11 [0.24; 107.24] | 4.46 [0.21; 93.83] | 6.31 [0.33; 121.13] | 10.15 [0.42; 245.84] | 9.58 [0.40; 231.97] | 3.79 [0.14; 102.61] | siponimod (0.25mg) | 0.04 [0.00; 0.73] | 0.16 [0.01; 3.39] | 0.13 [0.01; 2.64] | 0.10 [0.01; 1.89] | . |
| 0.20 [0.04; 0.96] | 0.17 [0.03; 0.87] | 0.15 [0.03; 0.75] | 0.24 [0.07; 0.85] | 0.30 [0.08; 1.07] | 0.61 [0.14; 2.69] | 0.17 [0.04; 0.66] | 0.36 [0.07; 1.98] | 0.32 [0.09; 1.15] | 0.28 [0.08; 1.05] | 0.15 [0.03; 0.91] | 0.21 [0.05; 0.87] | 0.18 [0.04; 0.77] | 0.26 [0.07; 0.88] | 0.41 [0.07; 2.30] | 0.39 [0.07; 2.17] | 0.15 [0.02; 1.05] | 0.04 [0.00; 0.73] | siponimod (0.5mg) | 3.88 [0.87; 17.29] | 3.25 [0.86; 12.34] | 2.42 [0.70; 8.37] | . |
| 0.76 [0.11; 5.06] | 0.67 [0.10; 4.56] | 0.57 [0.08; 3.92] | 0.92 [0.18; 4.77] | 1.17 [0.23; 6.01] | 2.36 [0.39; 14.45] | 0.66 [0.12; 3.65] | 1.41 [0.19; 10.29] | 1.25 [0.24; 6.46] | 1.10 [0.21; 5.83] | 0.60 [0.08; 4.67] | 0.80 [0.14; 4.73] | 0.70 [0.12; 4.14] | 0.99 [0.20; 4.98] | 1.60 [0.21; 11.89] | 1.51 [0.20; 11.22] | 0.60 [0.07; 5.28] | 0.16 [0.01; 3.39] | 3.88 [0.87; 17.29] | siponimod (1.25mg) | 0.84 [0.15; 4.53] | 0.62 [0.12; 3.14] | . |
| 0.64 [0.11; 3.75] | 0.56 [0.09; 3.38] | 0.47 [0.08; 2.91] | 0.77 [0.17; 3.46] | 0.98 [0.22; 4.35] | 1.98 [0.37; 10.63] | 0.55 [0.11; 2.66] | 1.18 [0.18; 7.67] | 1.05 [0.24; 4.68] | 0.92 [0.20; 4.24] | 0.50 [0.07; 3.50] | 0.67 [0.13; 3.47] | 0.59 [0.11; 3.04] | 0.83 [0.19; 3.60] | 1.34 [0.20; 8.87] | 1.26 [0.19; 8.36] | 0.50 [0.06; 3.97] | 0.13 [0.01; 2.64] | 3.25 [0.86; 12.34] | 0.84 [0.15; 4.53] | siponimod (10mg) | 0.75 [0.17; 3.24] | . |
| s | 0.74 [0.46; 1.17] | 0.93 [0.59; 1.47] | 1.89 [0.77; 4.63] | 0.53 [0.27; 1.03] | 1.13 [0.34; 3.80] | 1.00 [0.64; 1.58] | 0.88 [0.51; 1.53] | 0.48 [0.13; 1.80] | 0.64 [0.29; 1.45] | 0.56 [0.25; 1.27] | 0.80 [0.56; 1.12] | 1.28 [0.37; 4.44] | 1.21 [0.35; 4.19] | 0.48 [0.11; 2.16] | 0.13 [0.01; 2.38] | 3.11 [0.94; 10.30] | 0.80 [0.16; 3.90] | 0.96 [0.23; 4.01] | siponimod (2mg) | . | 0.45 [0.15; 1.39] | 0.74 [0.46; 1.17] |
| 0.40 [0.08; 2.14] | 0.66 [0.18; 2.46] | 0.83 [0.22; 3.10] | 1.68 [0.37; 7.73] | 0.47 [0.12; 1.91] | 1.00 [0.18; 5.67] | 0.89 [0.24; 3.33] | 0.78 [0.20; 3.03] | 0.42 [0.07; 2.60] | 0.57 [0.13; 2.51] | 0.50 [0.11; 2.20] | 0.71 [0.20; 2.55] | 1.14 [0.34; 3.76] | 1.07 [0.67; 1.71] | 0.42 [0.10; 1.84] | 0.11 [0.00; 2.80] | 2.76 [0.46; 16.41] | 0.71 [0.09; 5.59] | 0.85 [0.12; 5.96] | 0.89 [0.24; 3.36] | teriflunomide (14mg) | 0.40 [0.08; 2.14] | 0.66 [0.18; 2.46] |

Supplementary Table 8: League Table for SAEs


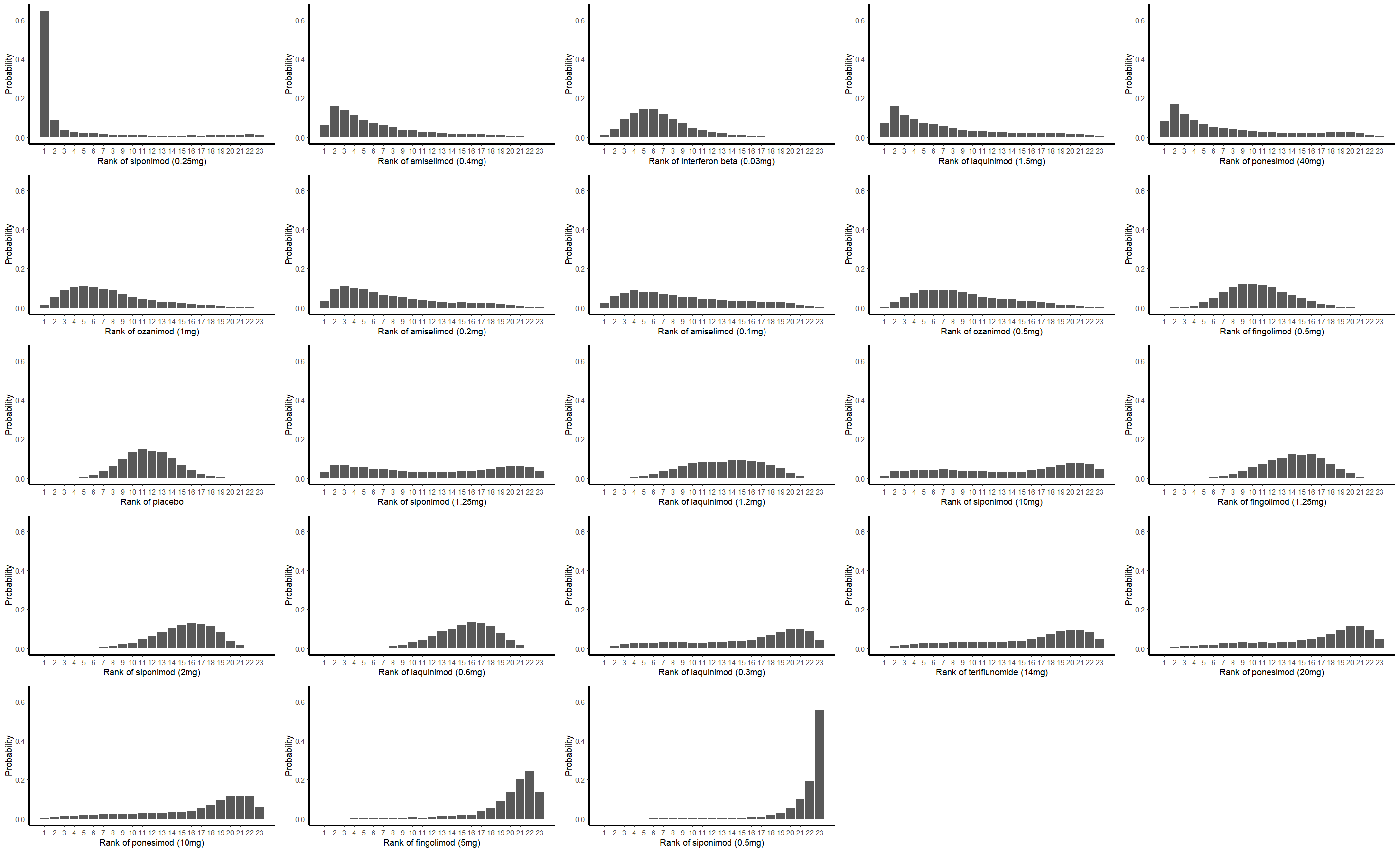


Supplementary Figure 19: Rank plot for SAEs


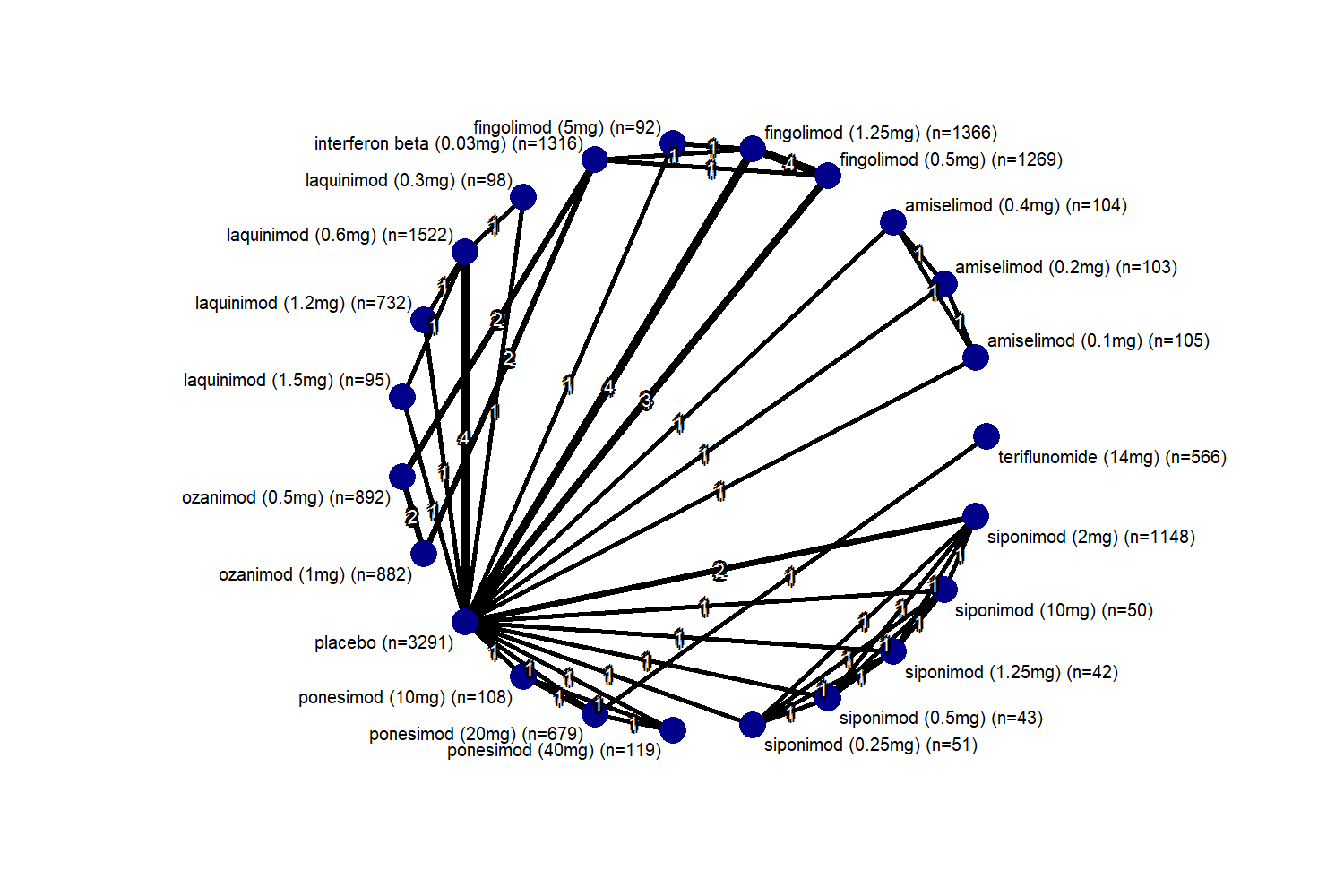


Supplementary Figure 20: Network plot for SAEs


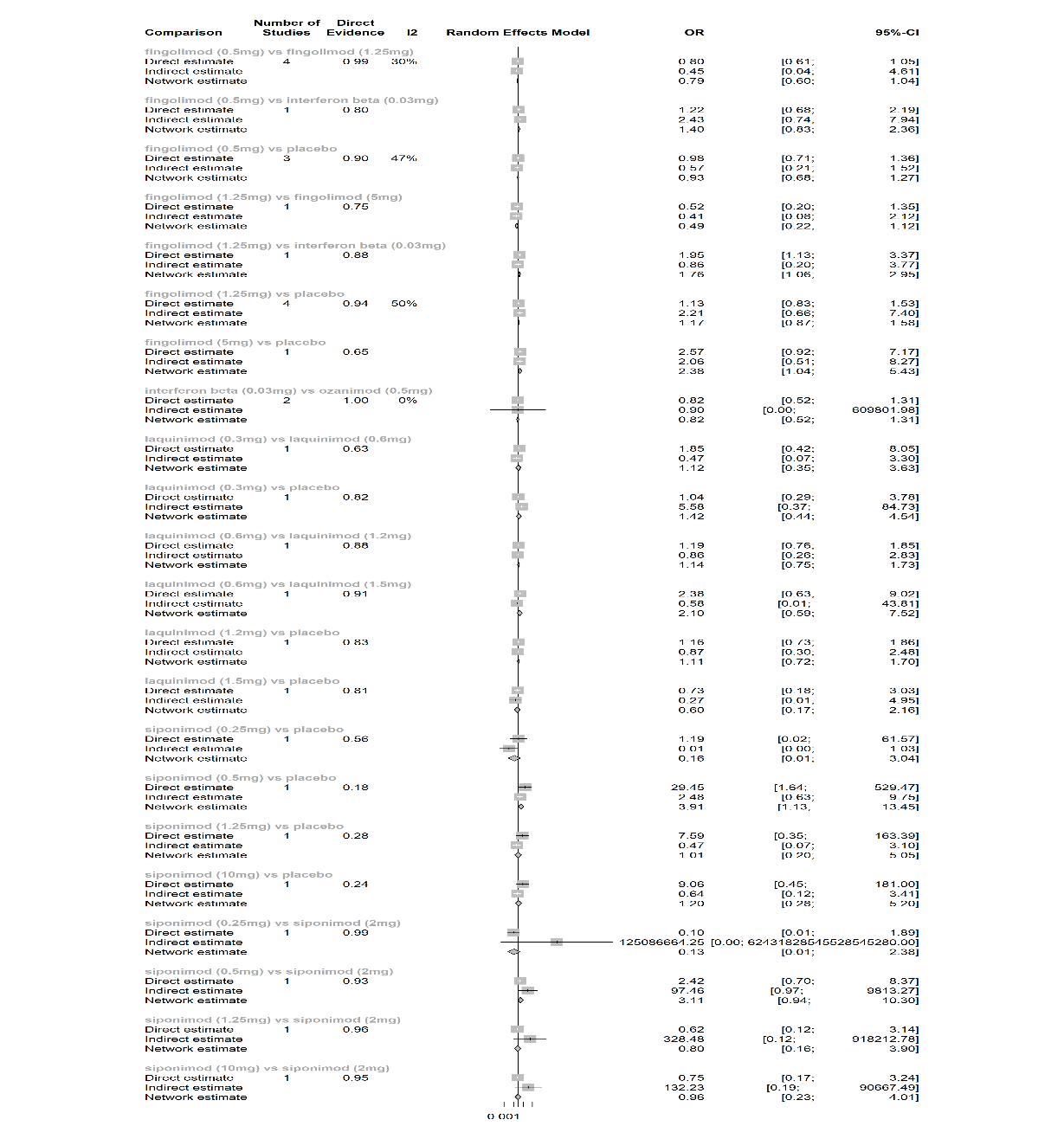


Supplementary Figure 21: Network split plot SAEs

| amiselimod (0.2mg) | 0.56 [0.13; 2.38] | . | . | . | . | . | . | . | . | . | . | 1.00 [0.21; 4.87] | . | . | . | . | . | . | . | . | . |
| --- | --- | --- | --- | --- | --- | --- | --- | --- | --- | --- | --- | --- | --- | --- | --- | --- | --- | --- | --- | --- | --- |
| 0.56 [0.13; 2.38] | amiselimod (0.4mg) | . | . | . | . | . | . | . | . | . | . | 1.79 [0.42; 7.59] | . | . | . | . | . | . | . | . | . |
| 0.54 [0.10; 2.83] | 0.96 [0.21; 4.45] | fingolimod (0.5mg) | 1.01 [0.64; 1.60] | . | 1.54 [0.59; 4.02] | . | . | . | . | . | . | 1.99 [1.14; 3.46] | . | . | . | . | . | . | . | . | . |
| 0.55 [0.10; 2.88] | 0.98 [0.21; 4.53] | 1.02 [0.65; 1.61] | fingolimod (1.25mg) | 0.60 [0.15; 2.32] | 2.88 [1.14; 7.27] | . | . | . | . | . | . | 1.50 [0.88; 2.54] | . | . | . | . | . | . | . | . | . |
| 0.39 [0.05; 2.83] | 0.70 [0.11; 4.54] | 0.72 [0.21; 2.49] | 0.71 [0.22; 2.32] | fingolimod (5mg) | . | . | . | . | . | . | . | 2.10 [0.50; 8.72] | . | . | . | . | . | . | . | . | . |
| 0.92 [0.16; 5.35] | 1.65 [0.32; 8.46] | 1.72 [0.81; 3.64] | 1.68 [0.80; 3.52] | 2.37 [0.61; 9.26] | interferon beta (0.03mg) | . | 0.97 [0.25; 3.72] | . | . | 1.69 [0.79; 3.59] | 1.31 [0.64; 2.71] | 2.03 [0.42; 9.68] | . | . | . | . | . | . | . | . | . |
| 1.50 [0.14; 15.53] | 2.67 [0.28; 25.37] | 2.79 [0.46; 16.72] | 2.72 [0.46; 16.29] | 3.84 [0.47; 31.22] | 1.62 [0.25; 10.53] | laquinimod (0.3mg) | 1.08 [0.13; 8.87] | . | . | . | . | 0.40 [0.07; 2.47] | . | . | . | . | . | . | . | . | . |
| 0.57 [0.10; 3.10] | 1.02 [0.21; 4.89] | 1.06 [0.49; 2.28] | 1.04 [0.49; 2.20] | 1.46 [0.39; 5.54] | 0.62 [0.26; 1.49] | 0.38 [0.07; 2.21] | laquinimod (0.6mg) | 1.46 [0.48; 4.45] | 1.39 [0.34; 5.75] | . | . | 1.91 [1.02; 3.58] | . | . | . | . | . | . | . | . | . |
| 0.85 [0.13; 5.72] | 1.52 [0.25; 9.14] | 1.59 [0.50; 5.08] | 1.55 [0.49; 4.93] | 2.19 [0.45; 10.75] | 0.92 [0.26; 3.28] | 0.57 [0.08; 4.16] | 1.50 [0.53; 4.20] | laquinimod (1.2mg) | . | . | . | 1.14 [0.35; 3.75] | . | . | . | . | . | . | . | . | . |
| 0.63 [0.08; 5.31] | 1.13 [0.15; 8.59] | 1.18 [0.26; 5.27] | 1.15 [0.26; 5.13] | 1.62 [0.25; 10.35] | 0.69 [0.14; 3.30] | 0.42 [0.05; 3.79] | 1.11 [0.29; 4.31] | 0.74 [0.14; 3.99] | laquinimod (1.5mg) | . | . | 3.03 [0.47; 19.46] | . | . | . | . | . | . | . | . | . |
| 1.54 [0.23; 10.42] | 2.75 [0.45; 16.66] | 2.86 [0.99; 8.31] | 2.80 [0.97; 8.06] | 3.95 [0.83; 18.77] | 1.67 [0.78; 3.55] | 1.03 [0.14; 7.72] | 2.70 [0.85; 8.61] | 1.81 [0.41; 7.89] | 2.44 [0.43; 13.95] | ozanimod (0.5mg) | 0.78 [0.36; 1.70] | . | . | . | . | . | . | . | . | . | . |
| 1.21 [0.18; 8.08] | 2.16 [0.36; 12.92] | 2.25 [0.79; 6.38] | 2.20 [0.78; 6.19] | 3.10 [0.66; 14.52] | 1.31 [0.64; 2.70] | 0.81 [0.11; 5.99] | 2.12 [0.68; 6.63] | 1.42 [0.33; 6.10] | 1.91 [0.34; 10.81] | 0.79 [0.36; 1.71] | ozanimod (1mg) | . | . | . | . | . | . | . | . | . | . |
| 1.00 [0.21; 4.87] | 1.79 [0.42; 7.59] | 1.86 [1.12; 3.09] | 1.82 [1.11; 2.98] | 2.57 [0.78; 8.47] | 1.08 [0.50; 2.33] | 0.67 [0.12; 3.74] | 1.76 [0.96; 3.22] | 1.17 [0.41; 3.38] | 1.58 [0.38; 6.56] | 0.65 [0.22; 1.90] | 0.83 [0.29; 2.37] | placebo | 0.20 [0.05; 0.89] | 0.46 [0.09; 2.22] | 0.16 [0.04; 0.70] | 1.69 [0.13; 21.31] | 0.26 [0.04; 1.61] | 1.39 [0.11; 17.53] | 0.14 [0.02; 0.76] | 0.55 [0.26; 1.17] | . |
| 0.20 [0.02; 1.77] | 0.36 [0.05; 2.87] | 0.38 [0.08; 1.80] | 0.37 [0.08; 1.75] | 0.52 [0.08; 3.49] | 0.22 [0.04; 1.16] | 0.14 [0.01; 1.31] | 0.36 [0.07; 1.76] | 0.24 [0.04; 1.47] | 0.32 [0.04; 2.50] | 0.13 [0.02; 0.82] | 0.17 [0.03; 1.03] | 0.20 [0.05; 0.89] | ponesimod (10mg) | 2.25 [0.65; 7.79] | 0.80 [0.28; 2.34] | . | . | . | . | . | . |
| 0.46 [0.05; 4.28] | 0.82 [0.10; 6.96] | 0.85 [0.16; 4.47] | 0.83 [0.16; 4.35] | 1.17 [0.16; 8.51] | 0.50 [0.09; 2.87] | 0.31 [0.03; 3.16] | 0.80 [0.15; 4.36] | 0.54 [0.08; 3.59] | 0.72 [0.09; 6.07] | 0.30 [0.04; 2.01] | 0.38 [0.06; 2.52] | 0.46 [0.09; 2.22] | 2.25 [0.65; 7.79] | ponesimod (20mg) | 0.36 [0.11; 1.20] | . | . | . | . | . | 1.49 [0.64; 3.45] |
| 0.16 [0.02; 1.40] | 0.29 [0.04; 2.26] | 0.30 [0.07; 1.41] | 0.30 [0.06; 1.37] | 0.42 [0.06; 2.74] | 0.18 [0.03; 0.91] | 0.11 [0.01; 1.04] | 0.29 [0.06; 1.38] | 0.19 [0.03; 1.15] | 0.26 [0.03; 1.97] | 0.11 [0.02; 0.64] | 0.14 [0.02; 0.81] | 0.16 [0.04; 0.70] | 0.80 [0.28; 2.34] | 0.36 [0.11; 1.20] | ponesimod (40mg) | . | . | . | . | . | . |
| 2.98 [0.19; 47.25] | 5.33 [0.36; 78.26] | 5.55 [0.54; 56.51] | 5.42 [0.53; 55.10] | 7.65 [0.59; 99.07] | 3.23 [0.30; 35.29] | 1.99 [0.12; 34.28] | 5.23 [0.50; 54.59] | 3.50 [0.29; 42.64] | 4.72 [0.33; 68.46] | 1.94 [0.16; 23.76] | 2.47 [0.20; 29.97] | 2.98 [0.31; 28.72] | 14.66 [0.98; 218.89] | 6.52 [0.41; 103.07] | 18.22 [1.24; 267.88] | siponimod (0.25mg) | 0.15 [0.02; 1.52] | 0.82 [0.05; 14.77] | 0.08 [0.01; 0.73] | 0.14 [0.01; 1.39] | . |
| 0.45 [0.05; 3.86] | 0.81 [0.10; 6.24] | 0.84 [0.18; 3.89] | 0.82 [0.18; 3.79] | 1.16 [0.18; 7.57] | 0.49 [0.10; 2.51] | 0.30 [0.03; 2.86] | 0.80 [0.17; 3.80] | 0.53 [0.09; 3.18] | 0.72 [0.09; 5.44] | 0.29 [0.05; 1.78] | 0.37 [0.06; 2.24] | 0.45 [0.11; 1.92] | 2.23 [0.28; 17.55] | 0.99 [0.12; 8.41] | 2.77 [0.36; 21.38] | 0.15 [0.02; 1.52] | siponimod (0.5mg) | 5.39 [0.54; 54.04] | 0.53 [0.13; 2.05] | 0.94 [0.22; 4.02] | . |
| 2.44 [0.15; 38.86] | 4.37 [0.30; 64.38] | 4.55 [0.44; 46.50] | 4.45 [0.44; 45.34] | 6.28 [0.48; 81.51] | 2.65 [0.24; 29.04] | 1.63 [0.09; 28.19] | 4.29 [0.41; 44.93] | 2.87 [0.23; 35.08] | 3.87 [0.27; 56.31] | 1.59 [0.13; 19.55] | 2.02 [0.17; 24.66] | 2.44 [0.25; 23.64] | 12.02 [0.80; 180.05] | 5.34 [0.34; 84.78] | 14.94 [1.01; 220.35] | 0.82 [0.05; 14.77] | 5.39 [0.54; 54.04] | siponimod (1.25mg) | 0.10 [0.01; 0.90] | 0.17 [0.02; 1.70] | . |
| 0.24 [0.03; 1.85] | 0.43 [0.06; 2.98] | 0.44 [0.11; 1.79] | 0.43 [0.11; 1.74] | 0.61 [0.10; 3.58] | 0.26 [0.06; 1.17] | 0.16 [0.02; 1.38] | 0.42 [0.10; 1.76] | 0.28 [0.05; 1.50] | 0.38 [0.05; 2.59] | 0.15 [0.03; 0.84] | 0.20 [0.04; 1.05] | 0.24 [0.06; 0.88] | 1.17 [0.16; 8.39] | 0.52 [0.07; 4.03] | 1.46 [0.21; 10.21] | 0.08 [0.01; 0.73] | 0.53 [0.13; 2.05] | 0.10 [0.01; 0.90] | siponimod (10mg) | 1.79 [0.48; 6.64] | . |
| 0.55 [0.09; 3.16] | 0.98 [0.19; 5.01] | 1.02 [0.41; 2.54] | 1.00 [0.40; 2.46] | 1.41 [0.34; 5.78] | 0.59 [0.20; 1.74] | 0.37 [0.06; 2.40] | 0.96 [0.36; 2.54] | 0.64 [0.17; 2.36] | 0.87 [0.17; 4.34] | 0.36 [0.10; 1.32] | 0.45 [0.12; 1.66] | 0.55 [0.26; 1.17] | 2.69 [0.51; 14.14] | 1.20 [0.21; 6.90] | 3.35 [0.65; 17.14] | 0.18 [0.02; 1.68] | 1.21 [0.31; 4.72] | 0.22 [0.02; 2.06] | 2.30 [0.68; 7.71] | siponimod (2mg) | . |
| 0.68 [0.06; 7.41] | 1.21 [0.12; 12.13] | 1.26 [0.20; 8.13] | 1.24 [0.19; 7.92] | 1.75 [0.20; 15.01] | 0.74 [0.11; 5.16] | 0.45 [0.04; 5.45] | 1.19 [0.18; 7.90] | 0.80 [0.10; 6.39] | 1.08 [0.11; 10.59] | 0.44 [0.05; 3.56] | 0.56 [0.07; 4.49] | 0.68 [0.11; 4.07] | 3.34 [0.75; 14.99] | 1.49 [0.64; 3.45] | 4.15 [0.95; 18.12] | 0.23 [0.01; 4.09] | 1.50 [0.15; 14.95] | 0.28 [0.02; 5.00] | 2.85 [0.31; 26.07] | 1.24 [0.18; 8.68] | teriflunomide (14mg) |

Supplementary Table 9: League table for DCAE


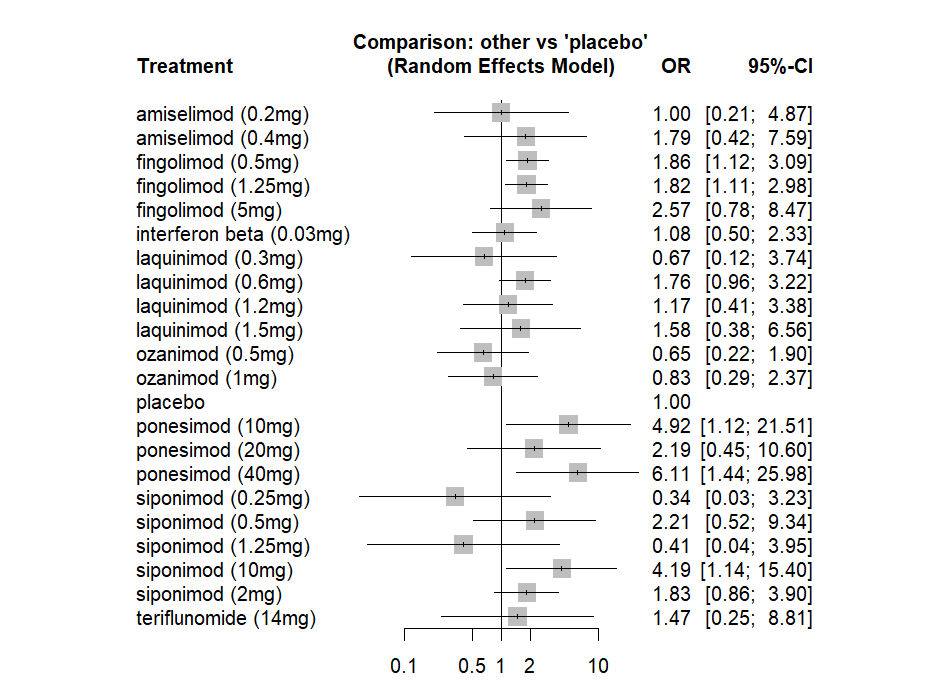


Supplementary Figure 22: Forest plot for DCAE


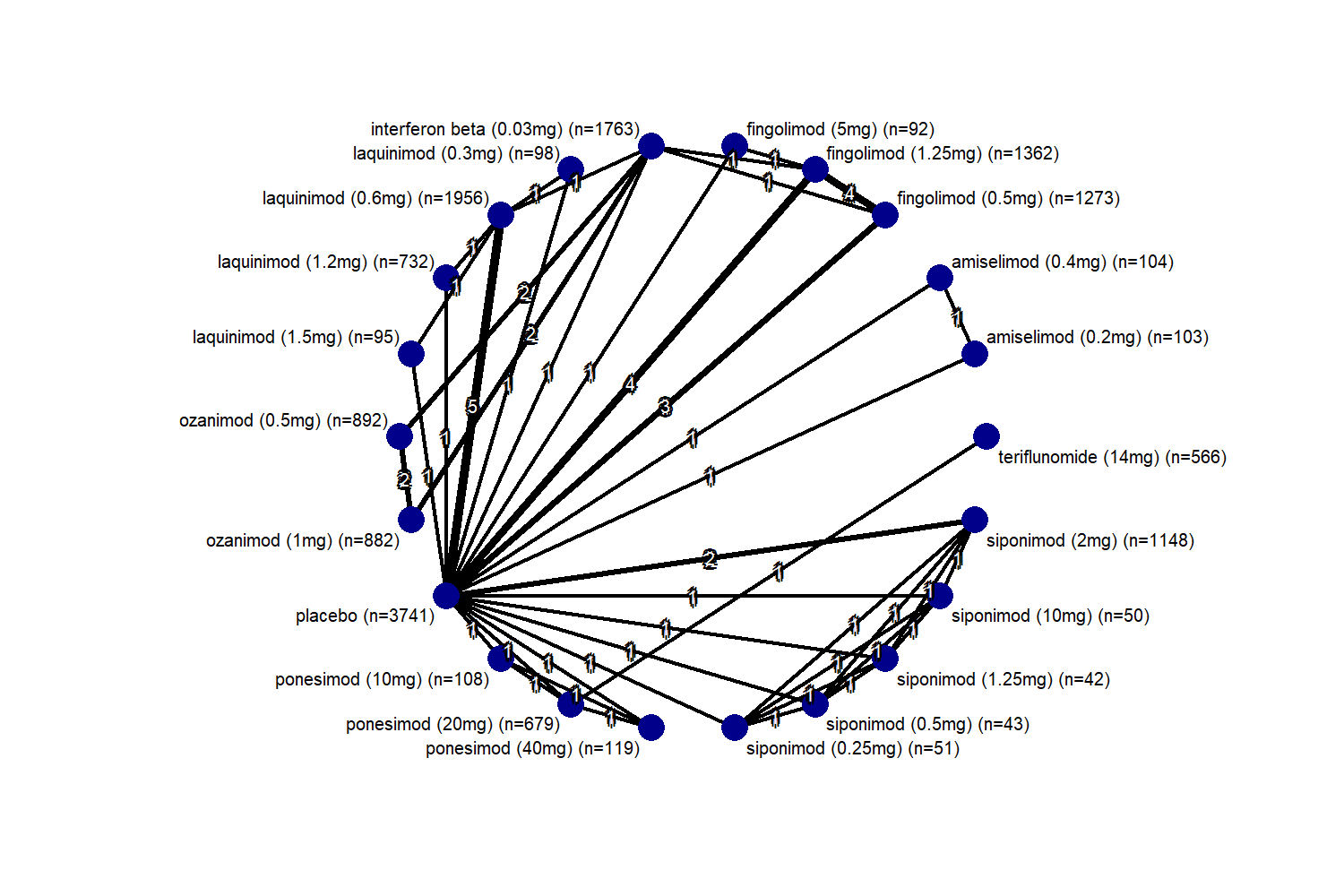


Supplementary Figure 23: Network plot for DCAE


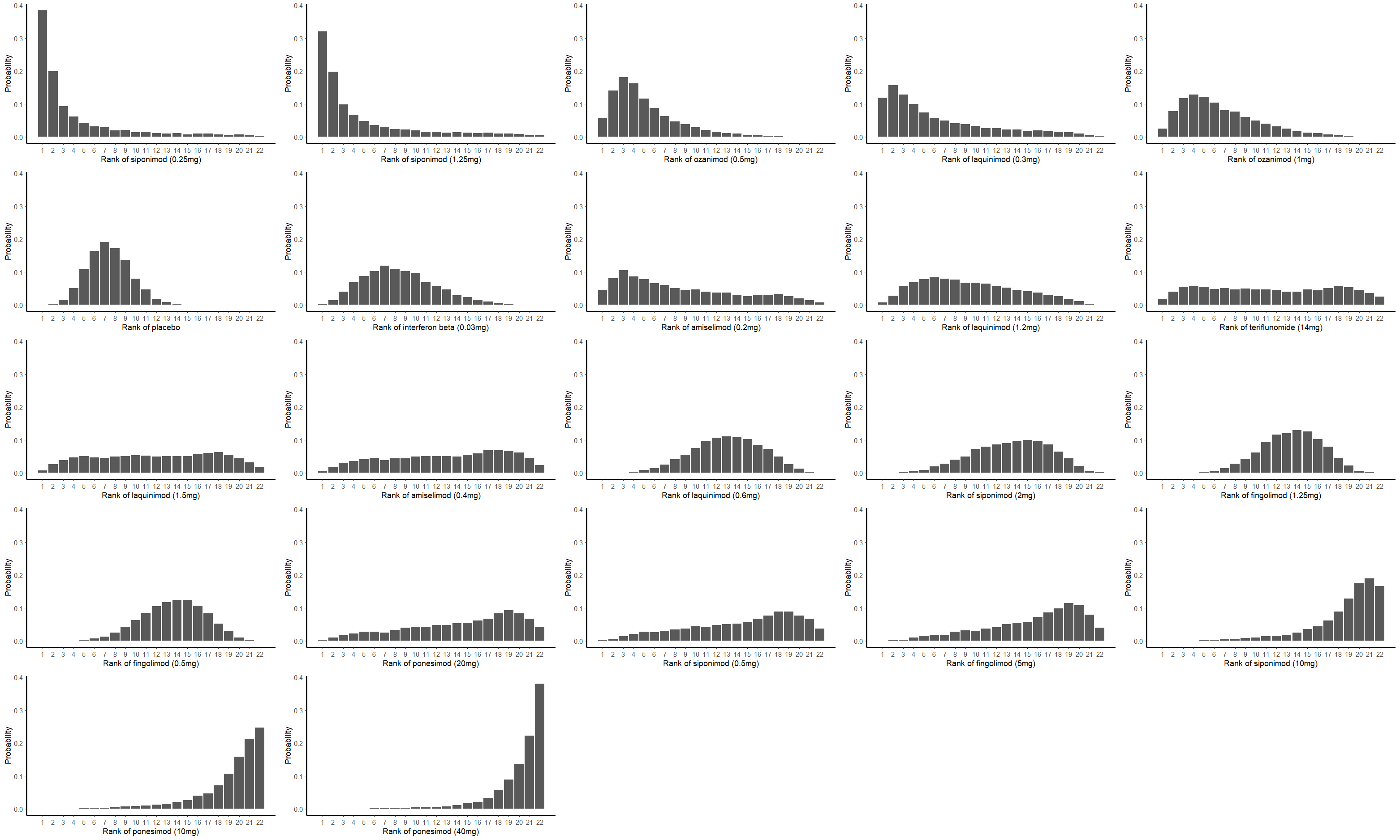


Supplementary Figure 24: Rank plot for DCAEs


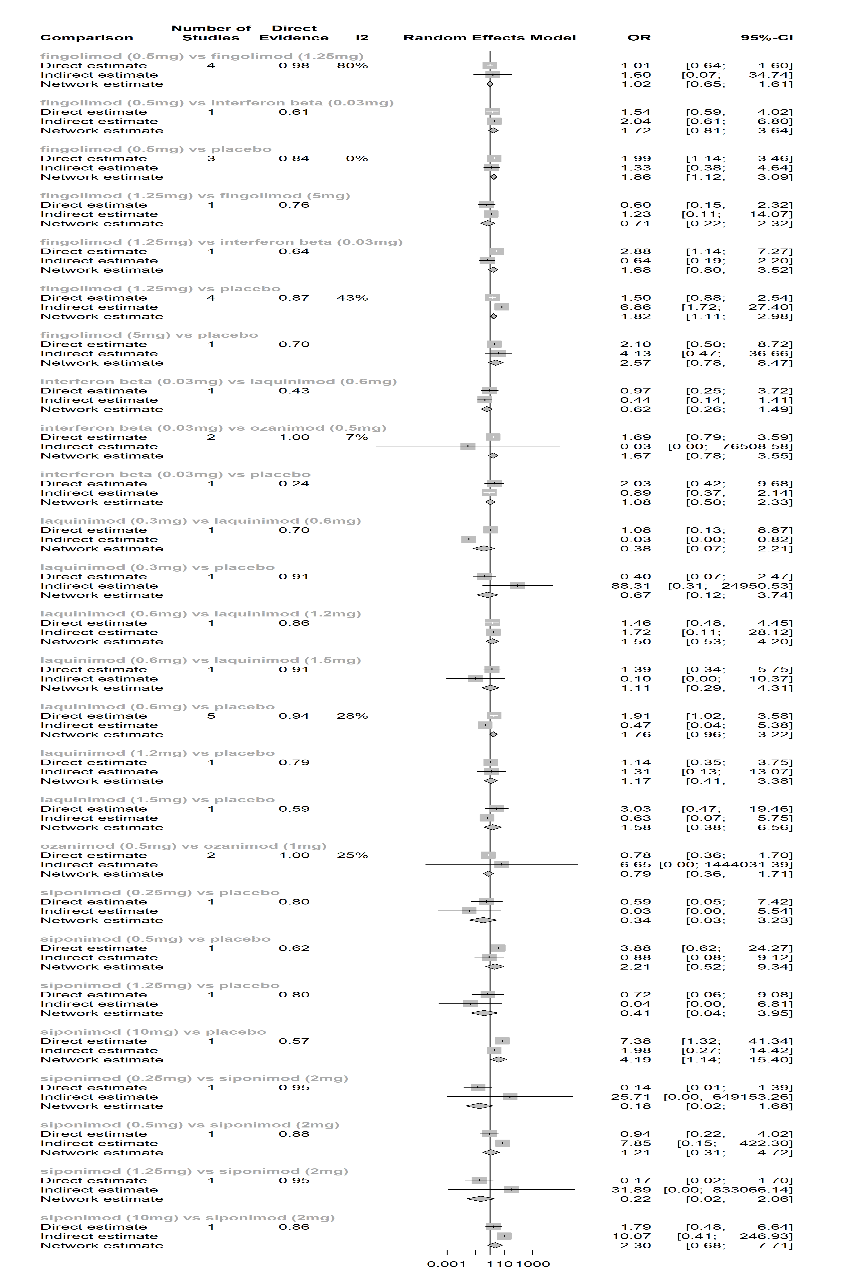


Supplementary Figure 25: Network split plot DCAEs


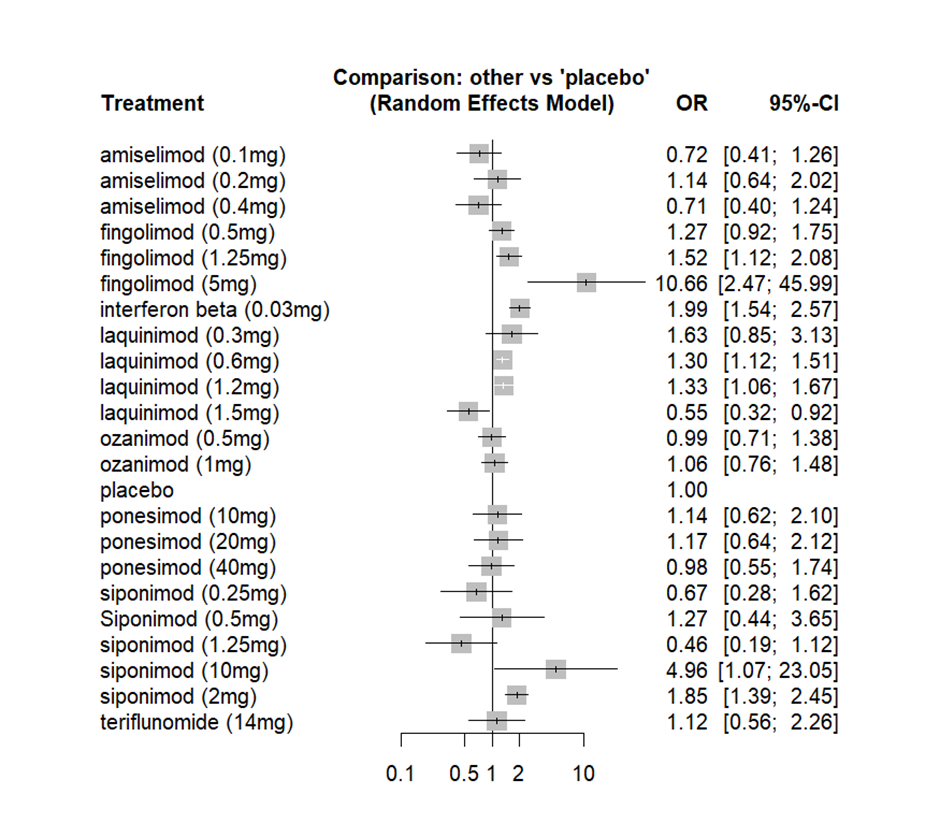


Supplementary Figure 26: Forest plot for Adverse Events


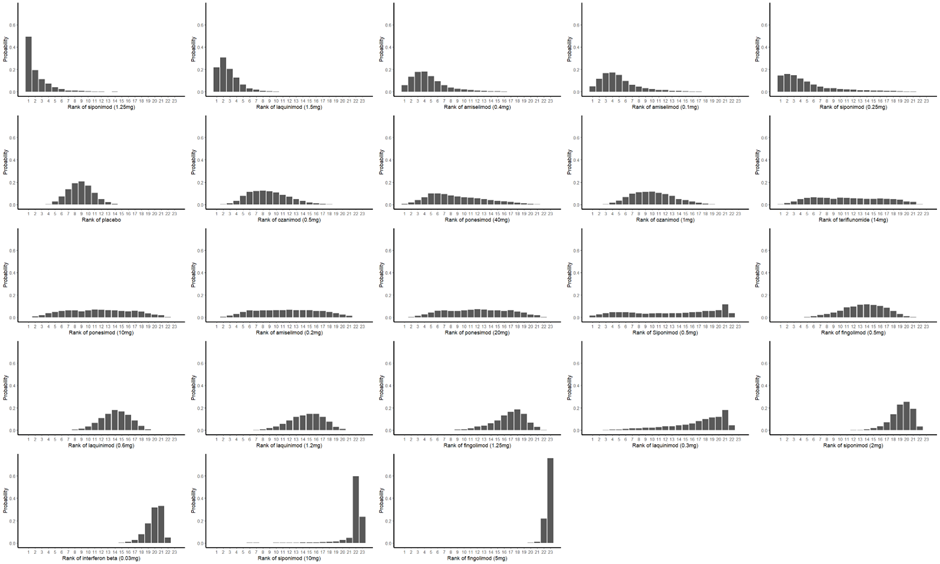


Supplementary Figure 27: Rank plot for Adverse Events


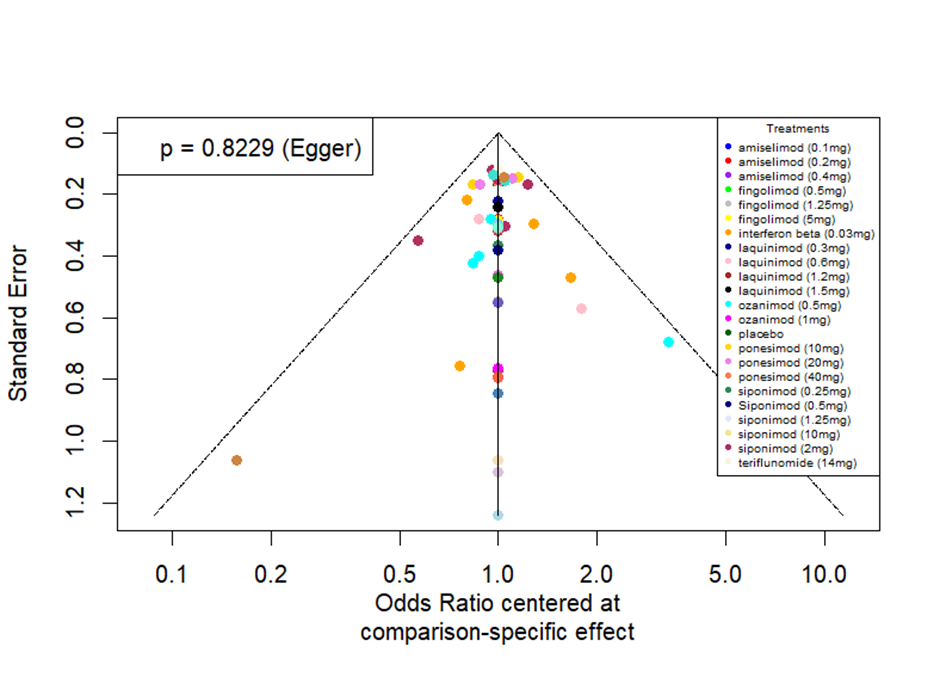


Supplementary Figure 28: Funnel plot


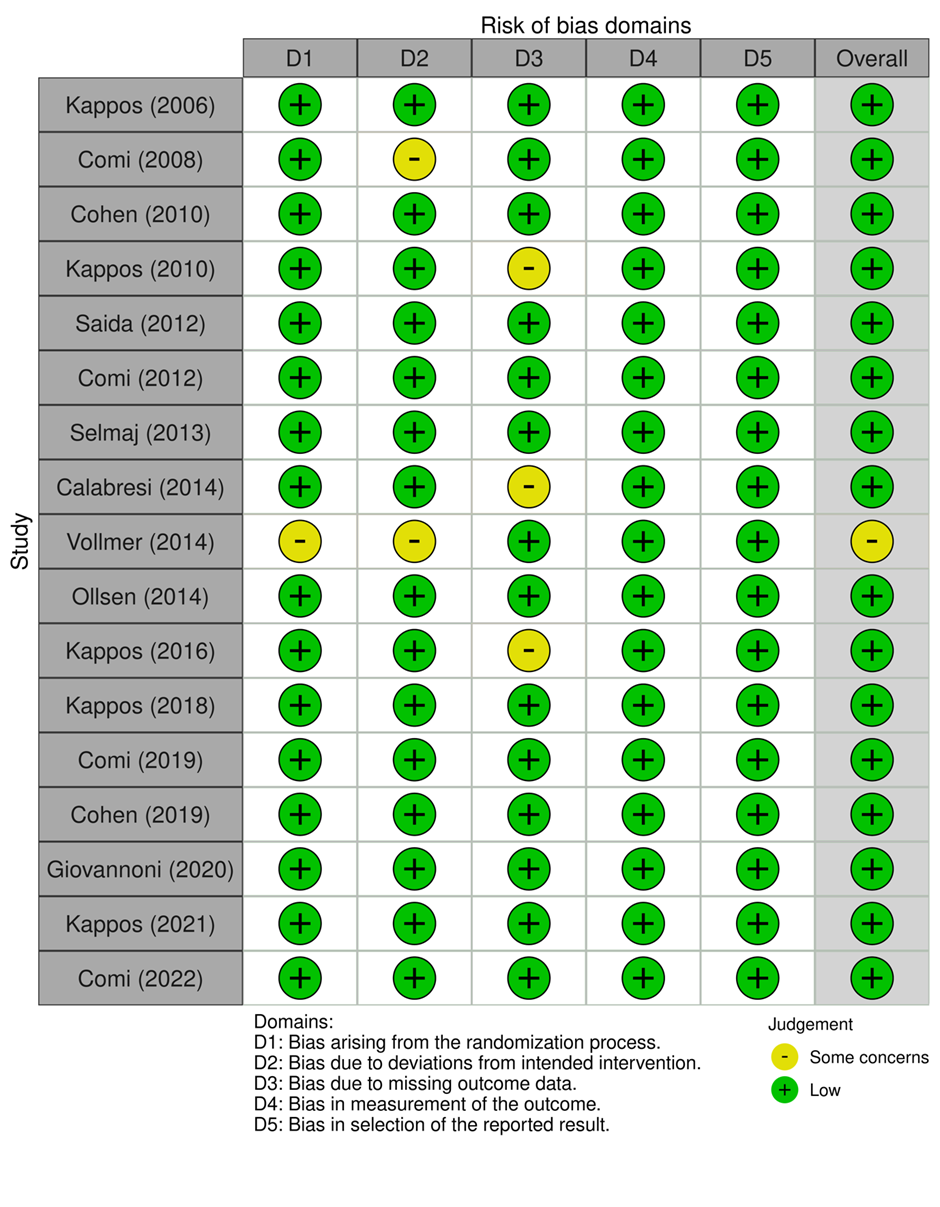


Supplementary Figure 29: Traffic plot for risk of bias
